# Universal Target Capture of HIV Sequences From NGS Libraries

**DOI:** 10.3389/fmicb.2018.02150

**Published:** 2018-09-13

**Authors:** Julie Yamaguchi, Ana Olivo, Oliver Laeyendecker, Kenn Forberg, Nicaise Ndembi, Dora Mbanya, Lazare Kaptue, Thomas C. Quinn, Gavin A. Cloherty, Mary A. Rodgers, Michael G. Berg

**Affiliations:** ^1^Infectious Diseases Research, Abbott Diagnostics, Chicago, IL, United States; ^2^National Institute of Allergy and Infectious Diseases, NIH, Baltimore, MD, United States; ^3^Institute of Human Virology, Abuja, Nigeria; ^4^Université de Yaoundé 1, Yaoundé, Cameroon; ^5^University of Bamenda, Bamenda, Cameroon; ^6^Université des Montagnes, Bangangté, Cameroon

**Keywords:** next-generation sequencing, HIV, HIV diversity, target enrichment, xGen, virus surveillance

## Abstract

**Background:** Global surveillance of viral sequence diversity is needed to keep pace with the constant evolution of HIV. Recent next generation sequencing (NGS) methods have realized the goal of sequencing circulating virus directly from patient specimens. Yet, a simple, universal approach that maximizes sensitivity and sequencing capacity remains elusive. Here we present a novel HIV enrichment strategy to yield near complete genomes from low viral load specimens.

**Methodology:** A non-redundant biotin-labeled probe set (HIV-xGen; *n* = 652) was synthesized to tile all HIV-1 (groups M, N, O, and P) and HIV-2 (A and B) strains. Illumina Nextera barcoded libraries of either gene-specific or randomly primed cDNA derived from infected plasma were hybridized to probes in a single pool and unbound sequences were washed away. Captured viral cDNA was amplified by Illumina adaptor primers, sequenced on a MiSeq, and NGS reads were demultiplexed for alignment with CLC Bio software.

**Results:** HIV-xGen probes selectively captured and amplified reads spanning the entirety of the HIV phylogenetic tree. HIV sequences clearly present in unenriched libraries of specimens but previously not observed due to high host background levels, insufficient sequencing depth or the extent of multiplexing, were now enriched by >1,000-fold. Thus, xGen selection not only substantially increased the depth of existing sequence, but also extended overall genome coverage by an average of 40%. We characterized 50 new, diverse HIV strains from clinical specimens and demonstrated a viral load cutoff of approximately log 3.5 copies/ml for full length coverage. Genome coverage was <20% for 5/10 samples with viral loads <log 3.5 copies/ml and >90% for 35/40 samples with higher viral loads.

**Conclusions:** Characterization of >20 complete genomes at a time is now possible from a single probe hybridization and MiSeq run. With the versatility to capture all HIV strains and the sensitivity to detect low titer specimens, HIV-xGen will serve as an important tool for monitoring HIV sequence diversity.

## Introduction

As the HIV epidemic continues, surveillance of HIV diversity is essential to monitor the emergence of new subtypes and the presence of new strains (Hemelaar, 2013). Knowing which subtypes or recombinants historically predominate in a geographic region and whether the identity and proportion of new infections reflect a static or changing landscape will help inform appropriate interventions. For example, in Europe where the majority were once subtype B, 50% of new infections are non-B or recombinants (Semaille et al., 2007). The number of non-B infections in the US has also been on the rise (Pyne et al., 2013; Oster et al., 2017). Of equal concern is knowing whether mutations have arisen in current strains that could lead to misdiagnosis of HIV status, under-quantification of patients on antiretroviral therapy, or inadequate screening of the blood supply (Brennan et al., 2006).

Next generation sequencing (NGS) has been applied to the fight against HIV in several capacities, including monitoring for drug resistance, cell tropism determination, superinfection studies, network analysis and transmission patterns, and intra-patient quasi-species detection (Bimber et al., 2010; Archer et al., 2012; Redd et al., 2012; Swenson et al., 2012; Giallonardo et al., 2014). These typically involve high-throughput amplicon sequencing of defined sub-genomic regions such as *pol* or *env*. By contrast, metagenomic approaches using random priming permit an assessment of the full extent of sequence diversity in strains and can be applied to surveillance. Complete genomes of HIV and co-infecting viruses can be obtained directly from patient specimens, provided viral loads are high enough (Luk et al., 2015; Yamaguchi et al., 2017). To increase sensitivity, the HIV-SMART method utilizes reverse primers in conserved sequences of HIV fused to a tag to facilitate combined virus-specific priming and amplification (Berg et al., 2016; Rodgers et al., 2017a). In an alternate HIV-specific approach, individual long fragments (e.g., >2 kb) amplified with HIV primer pairs can be converted to NGS libraries to achieve detection limits nearing that of PCR, although the process is more labor intensive (Gall et al., 2012). What remains elusive is a simple, universal NGS approach for surveillance that balances sensitivity and cost, allowing numerous specimens of any titer and from any HIV group to be sequenced simultaneously while also maximizing NGS capacity and minimizing the use of resources.

To increase the sensitivity of microbial metagenomics from patient specimens, numerous pre-treatment approaches have been applied to reduce host background, including nuclease pre-treatment, rRNA depletion, filtration, centrifugation, etc. (Hall et al., 2014; Conceição-Neto et al., 2015). An alternate means of enriching for viral reads after NGS library preparation has shown great promise. Briese, et al synthesized nearly 2 million biotin-labeled probes to cover all coding regions of vertebrate viruses, hybridized them to conventional high throughput sequencing libraries containing spiked in viral nucleic acid, and amplified sequences captured on streptavidin beads. The VirCapSeq-VERT method yielded a 100–10,000-fold increase in viral reads from patient specimens (Briese et al., 2015). An analogous approach tailored specifically for hepatitis C virus (HCV) has been equally successful, tiling all seven genotypes with just 1,100 probes (Bonsall et al., 2015). HIV probes tiling only subtype B showed in principle that genomes of integrated provirus can be selectively amplified from host DNA (Miyazato et al., 2016). As with HCV, HIV-1 and HIV-2 exhibit substantial diversity and therefore probes inclusive of all subtypes and groups are needed for surveillance. Here we developed a comprehensive probe set to accommodate the full spectrum of HIV strains encountered. We demonstrate that the HIV-xGen method is highly effective, particularly for low titer patient specimens, moving the field closer toward a universal, sensitive, high volume NGS solution.

## Materials and methods

### Specimens

Specimens were collected by the Abbott Global Surveillance Program through collaborations in Cameroon, Uganda, South Africa, Senegal, and Thailand. All specimens were de-identified and obtained according to local regulations in each country at the time of collection between 1998 and 2016, including local IRB approval when required. Specimens were identified as HIV-1 positive by rapid diagnostic tests done in source countries before sequence analysis and testing at Abbott Laboratories (Swanson et al., 2000; Brennan et al., 2008; Rodgers et al., 2017b). De-identified specimens from the Democratic Republic of Congo were obtained in 1987 as part of Project SIDA by the US National Institute of Allergy and Infectious Diseases (Cohen, 1997).

### Specimen pretreatment and extraction

Plasma specimens were pre-treated with Ultra-pure benzonase (Sigma, St. Louis MO) for 3 h at 37°C and extracted on the *m*2000*sp* (Abbott Laboratories, Des Plaines IL) with the RNA protocol (500 μl input/70 μl elute) as described (Berg et al., 2016).

### HIV viral loads

Viral loads were determined by the HIV-1 RealTi*m*e (Abbott Laboratories, Des Plaines IL) assay as described (Berg et al., 2016).

### cDNA synthesis and nextera XT library production

Viral RNA was concentrated to 10 μl with RNA Clean and Concentrator-5 spin columns (Zymo Research, CA) as described (Rodgers et al., 2017a). For gene-specific reverse transcription, cDNA was generated with HIV-SMART essentially as described (Berg et al., 2016; Rodgers et al., 2017a). Minor modifications described here were made to primer concentrations, cDNA input and PCR cycling conditions to reduce loss and boost sensitivity. The pool of six HIV-SMART primers used was 300 nM final for each (formerly 1 μM), the entire 10 μl of SMART cDNA (without dilution) was used as input for SMART amplification, and 18 rounds (formerly 35) of PCR were performed. HIV-SMART libraries were then purified with 1.4X vol of AMP-pure beads, eluted in 12 μl of EB buffer, and 5 μl (undiluted) was used as template for Nextera XT reactions. For reverse transcription by random primers, metagenomic libraries were prepared by using Superscript III (SSRTIII) 1st Strand reagents (Life Technologies) followed by 2nd strand synthesis with Sequenase V2.0 T7 DNA pol (Affymetrix). Double stranded cDNA was recovered with DNA clean and concentrator spin columns (Zymo Research) and eluted in 7 μl. HIV-SMART and SSRTIII libraries were tagmented by Nextera XT and amplified for 16 and 24 cycles, respectively, with Set A indices lacking 5′ biotin tags (see below), according to manufacturer instructions (Illumina, Carlsbad CA). Nextera libraries were purified with Agencourt AMPpure XP beads (Beckman Coulter), visualized on a BioAnalyzer TapeStation (Agilent), and quantified on a Qubit instrument (Life Technologies) with dsDNA broad range reagents (Molecular Diagnostics).

### Design of HIV xGen probe set

For HIV-1 group M, approximately 10 complete reference sequences from each subtype (A-K, plus CRF02) and representing a diversity of countries of origin were selected from the Los Alamos National Lab 2015 alignment. HIV-xGen probes (*n* = 82) were first designed against the consensus sequence derived from this reduced alignment. All degenerate base codes (e.g., R, Y) were replaced with specific, majority base call designations to permit probe synthesis. Next, the consensus was compared to individual subtypes by scanning each sequence in 100 bp windows to identify regions with <80% identity. Where diversity exceeded this cutoff and substitutions were found to be common across several subtypes, additional probes (*n* = 101) tiling 122–366 bp segments were designed to ensure complete capture of these regions. In order to adequately tile stretches with >10% diversity (e.g., *env*), probes were either designed against the entire individual sequence or to those sequences flanking the variable region. Consensus sequences were similarly obtained to design probe sets for HIV-1 groups N (*N* = 78), O (*N* = 83) and P (*N* = 83), and HIV-2 groups A (*N* = 87) and B (*N* = 86), each supplemented with additional probes (*N* = 11, 31, 6, 3, and 0, respectively) covering regions of increased diversity.

### Synthesis of HIV xGen probes, nextera barcoding primers, and blocking oligos

120 nt probes encoding the sense strand of HIV with 1 nt overlap and modified with a 5′ biotin tag were synthesized at Integrated DNA Technologies (IDT, Coralville IA). Mini-pools made at 3 pmol/probe were resuspended in 15 μl of TE to a storage concentration of 0.2 pmol/probe/μl.

Custom DNA Ultramers (4 nmol) of Nextera XT Set A indices (N701-N715 and S502-S511) lacking a biotin label and containing two 3′ phosphothioate modifications were synthesized at IDT and working stocks were each diluted to 5 μM. Blocking oligos complementary to Nextera Set A i5 and i7 index primers (e.g., P5/P7 adaptors_8 nt barcodes_R1/R2 sequencing primers) were also synthesized at IDT at a 1 μl/reaction working concentration.

### xGen hybridization, washes and library amplification

The enrichment for HIV reads was performed essentially as described in the *Hybridization capture of DNA libraries using xGen*® *Lockdown*® *probes and reagents* protocol from IDT. Individually barcoded Nextera libraries (*n* = 6–26) were pooled to have a minimum 500 ng of cDNA and then combined with 5 μg of Cot-1 DNA carrier and 1 μl of each blocking oligo. Samples were dried for >15 min in a SpeedVac set at 45°C. Pelleted material was resuspended in 8.5 μl of 2X hybridization buffer, 2.7 μl of Hybridization Buffer Enhancer, and 1.8 μl of nuclease-free water. After a 10 min denaturation at 95°C, 4 μl of a working probe stock (100 attomoles/probe/μl) was added and mixed to bring the final volume to 17 μl. Hybridizations were incubated for 4 h at 65°C. M-270 Streptavidin beads (100 μl per capture) were equilibrated in Bead Wash buffer, pelleted by magnet, and mixed with hybridizations for another 45-min incubation at 65°C, vortexing every 12 min to ensure beads remained in solution. Washes were performed as recommended and beads were resuspended in 20 μl of nuclease-free water for an initial 12 cycles of amplification with the KAPA PCR mix. Agencourt AMPpure beads (1.5X volume/75 μl) were added to PCR reactions and captured/washed material was eluted in 20 μl. A repeat KAPA amplification of 10 cycles followed by AMP-Pure clean-up was performed and libraries were visualized on an Agilent 2200 TapeStation and quantified with a Qubit fluorometer using the dsDNA high-sensitivity kit.

### NGS analysis

Analysis was performed as described (Berg et al., 2016; Rodgers et al., 2017a). Barcodes were parsed on the MiSeq instrument and filtered for Q-scores above 30. Fastq files were imported into CLC Genomics Workbench 9.0 software (CLC bio/Qiagen, Aarhus Denmark, version 9.0) where reads below 70 nt were discarded and the SMART adaptor was removed separately from both strands. When no preliminary Sanger sequence was available, raw data was simultaneously mapped to multiple HIV reference sequences to determine the subtype/group with the greatest identity. The following alignment settings were applied: match = 1, mismatch = 2, insertion = 3, deletion = 3, length fraction = 0.7, and similarity fraction = 0.8. An iterative approach was used to derived the final sequence, whereby the initial consensus served as the reference to refine the consensus in a second round of alignment. The raw NGS data (see below) was realigned to the final consensus sequence to generate NGS run statistics found in Tables 1, 2.

**Table 1:** NGS data for samples sequenced ± enrichment with HIV xGen.

**Specimen ID**	**Country**	**Group or M subtype**	**Viral Load**	**Input copy no**.	**Library type**	**Total reads**	**HIV reads**	**% HIV reads**	**Genome coverage%**	**Avg. coverage depth**	**Std. Dev**.
O-LA34	Cameroon	HIV-1 O	8.06	8019	^*^HIV-SMART	1,336,488	50,865	3.80	100	986	589
–	–	–	–	20667	SSIII-xGen	1,477,954	1,436,992	97.23	100	14,576	11,026
N-LA28	Cameroon	HIV-1 N	7.61	nt	^*^HIV-SMART	1,564,286	155,741	10.00	100	3,422	1,604
–	–	–	–	7333	SSIII-xGen	2,033,250	2,020,867	99.39	100	22,538	17,020
2A-LA38	Senegal	HIV-2A	6.95		^*^Ovation single cell	3,029,490	40,402	1.33	100	573.43	445.55
–	–	–	–	28355	SSIII	37,384	152	0.41	72	2	2
–	–	–	–	–	SSIII-xGen	342,284	331,623	96.89	100	3,400	3,344
C-8119636	South Africa	HIV-1 C	5.62	8870	^*^HIV-SMART	5,446,060	367,224	6.74	99	6,481	4,483
–	–	–	–	85260	SSIII	586,936	15800	2.69	98	156	196
–	–	–	–	–	SSIII-xGen	400,458	393,350	98.23	100	4,042	5,648
02-421-10	Cameroon	HIV-1 CRF02	4.48	643	^*^HIV-SMART	1,418,404	42,153	2.97	61	1,201	1,127
–	–	–	–	6177	SSIII	412,134	80	0.02	37	1	1
–	–	–	–	–	SSIII-xGen	9,776	7,131	72.94	82	78	231
PBS1342^†^	DRC	HIV-1 URF	4.59	3830	HIV-SMART	662,360	3,511	0.53	86	19	94
–	–	–	–	–	HIV-SMART-xGen	691,384	669,074	96.77	95	8,673	14,978
–	–	–	–	–	SSIII	3,432,778	1,095	0.03	99	11	8
–	–	–	–	–	SSIII-xGen	786,548	747,840	95.07	97	8,746	13,219
PBS1191	DRC	HIV-1 G	3.86	1426	HIV-SMART	411,612	2,067	0.50	51	7	94
–	–	–	–	–	HIV-SMART-xGen	2,156,988	1,504,919	69.77	100	17,823	21,968
–	–	–	–	–	SSIII	1,276,656	306	0.02	45	2	4
–	–	–	–	–	SSIII-xGen	3,968,286	3,313,601	83.50	96	36,912	126,521
PBS888	DRC	HIV-1 A	3.47	581	HIV-SMART	1,037,832	208	0.02	56	2	4
–	–	–	–	–	HIV-SMART-xGen	2,172,982	1,880,055	86.52	100	25,171	53,285
–	–	–	–	–	SSIII	4,532,554	257	0.01	54	2	2
–	–	–	–	–	SSIII-xGen	1,699,092	925,860	54.49	96	10,689	23,552
70641	DRC	HIV-1 A1	5.78		HIV-SMART	1,361,655	51,646	3.79	99	947	1,385
	–	–	–	–	HIV-SMART-xGen	9,759,614	9,158,341	93.84	100	193,337	648,377
PBS5635^†^	DRC	HIV-1 D	5.31		HIV-SMART	1,255,489	12,653	1.01	95	259	320
	–	–	–	–	HIV-SMART-xGen	574,273	522,043	90.91	99	12,321	30,529
P406	DRC	HIV-1 G	5.21		HIV-SMART	2,206,004	4,676	0.20	98	76	98
	–	–	–	–	HIV-SMART-xGen	1,238,978	629,912	50.84	100	14,903	40,689
PBS1189^‡^	DRC	HIV-1 F1	4.98		HIV-SMART	1,978,086	2,260	0.11	82	41	76
	–	–	–	–	HIV-SMART-xGen	522,902	463,644	88.67	86	9,236	41,558
50^†^	DRC	HIV-1 A1 basal	4.96		HIV-SMART	1,662,501	1,731	0.10	83	29	38
	–	–	–	–	HIV-SMART-xGen	237,883	218,072	91.67	83	4,899	15,280
87-2580^†^	DRC	HIV-1 G	4.85		HIV-SMART	2,200,708	5,741	0.26	96	112	167
	–	–	–	–	HIV-SMART-xGen	529,218	221,335	41.82	96	5,088	10,026
PBS6126	DRC	HIV-1 A1	4.67		HIV-SMART	149,709	115	0.08	79	2.6	2.4
	–	–	–	–	HIV-SMART-xGen	28,606	11,644	40.70	100	4,034	276
2049^‡^	DRC	HIV-1 CRF37 basal	4.31		HIV-SMART	2,720,263	1,677	0.06	73	33	46
	–	–	–	–	HIV-SMART-xGen	10,689,440	9,321,568	87.20	90	201,793	340,069
PBS0724^‡^	DRC	HIV-1A1	4.02		HIV-SMART	107,044	1	0.00	2	0.02	0.16
	–	–	–	–	HIV-SMART-xGen	2,010,039	1,061,113	52.79	100	21,120	38,865
PBS1195	DRC	HIV-1 A2	3.77		HIV-SMART	605,867	51	0.01	54	1.2	1.4
	–	–	–	–	HIV-SMART-xGen	85,628	45,233	52.83	100	1,039	1,847
2106	DRC	HIV-1 A1	3.76		HIV-SMART	1,664,855	598	0.04	80	13	14
	–	–	–	–	HIV-SMART-xGen	1,355,794	1,054,751	77.80	100	24,313	57,096
P4039	DRC	HIV-1 A1	3.58		HIV-SMART	419,163	132	0.03	71	2.8	3
	–	–	–	–	HIV-SMART-xGen	398,324	228,302	57.32	100	5,084	6,066
P3844	DRC	HIV-1 K	4.67		HIV-SMART	37,076	7	0.02	8	1.27	0.59
	–	–	–	–	HIV-SMART-xGen	1,883,032	992,447	52.7	97	23,300	57,444

* Previously published data reported in Berg et al. (2016).

† Genome completed (100%) with Sanger.

‡ *Genome coverage increased with Sanger but still incomplete*.

**Table 2:** NGS data for samples sequenced only by HIV xGen.

**Specimen**	**Country**	**HIV group or M subtype**	**Viral load (Log_10_)**	**Library input (copies)**	**Library type**	**Total reads**	**HIV reads**	**% HIV reads**	**Genome coverage%**	**Avg coverage depth**	**Standard deviation**
459-16	Cameroon	HIV-1 CRF02	5.31	41,345	SSIII-xGen	8,303,330	8,043,055	96.87	100	108,492.77	214,888.09
A1699	Cameroon	HIV-1 F2	5.04	21,587	HIV SMART-xGen	5,380,619	5,336,115	99.17	100	70,586.38	117,687.56
A1786	Cameroon	HIV-1 G	4.73	10,573	HIV SMART-xGen	108,242	93,856	86.71	83	1,247.45	2,496.38
8128965^‡^	South Africa	HIV-1 C	4.50	6,226	HIV SMART-xGen	243,571	232,511	95.46	95	3,017.97	8,065.69
144-26^†^	Cameroon	HIV-1 G	4.03	2,110	HIV SMART-xGen	247,845	219,844	88.70	93	2,941.81	4,593.91
A1185	Cameroon	HIV-1 CRF01	3.87	1,459	HIV SMART-xGen	96,329	69,459	72.11	79	940.92	2,784.90
5056135	Senegal	HIV-1 C	3.67	921	HIV SMART-xGen	517,740	454,502	87.79	100	6,237.01	9,850.11
577-27^‡^	Cameroon	HIV-1 A	3.67	921	HIV SMART-xGen	111,404	94,003	84.38	79	1,278.21	3,069.71
42-877	Uganda	HIV-1 D	3.66	900	HIV SMART-xGen	313,662	279,983	89.26	100	3,829.33	5,495.33
112-11^‡^	Cameroon	HIV-1 F2	3.63	840	HIV SMART-xGen	204,740	158,815	77.57	91	2,236.33	3,634.36
P4142	DRC	HIV-1 A1	3.29	383	HIV SMART-xGen	814501	24	0.29	34	0.34	0.70
2528	DRC	HIV-1 A1	3.26	358	HIV SMART-xGen	553,492	0	0	0	0	0
PBS70-233	DRC	HIV-1 A1	3.24	342	HIV SMART-xGen	1,322	0	0	0	0	0
PBS369-87	DRC	HIV-1 A1	3.18	297	HIV SMART-xGen	947,145	30	0.31	14	0.42	1.13
475-17	Cameroon	HIV-1 A	3.00	262	HIV SMART-xGen	238,092	1,753	0.74	20	21.24	73.91
669-17	Cameroon	HIV-1 CRF22	2.93	172	SSIII-xGen	119,116	38,654	32.45	51	509.50	1584.65
129-26^‡^	Cameroon	HIV-1 D	2.82	173	HIV SMART-xGen	441,846	40,680	9.21	53	579.49	2,232.28
844-55	Cameroon	HIV-1 B/02	2.77	155	HIV SMART-xGen	224,916	2,542	1.13	18	28.16	121.64
5020-19	Cameroon	HIV-1 C	2.76	151	HIV SMART-xGen	602,884	1,444	0.24	12	6.80	60.30
4188-11	Cameroon	HIV-1 CRF11	2.75	148	HIV SMART-xGen	702,804	516	0.07	12	5.58	22.09
10047105267	Thailand	HIV-1 CRF01	2.72	138	HIV SMART-xGen	893,628	149,379	16.72	42	2,126.61	10,928.35
193-6	Cameroon	HIV-1 A	2.65	117	HIV SMART-xGen	370,450	496	0.13	14	3.37	14.69
961-09^‡^	Cameroon	HIV-1 CRF37	2.43	71	HIV SMART-xGen	229,794	197	0.09	10	1.45	9.31
363-24^†^	Cameroon	HIV-1 CRF13	nd	n/a	HIV SMART-xGen	133,832	117,148	87.53	85	1,478.12	4,140.94
1173-31	Cameroon	HIV-1 CRF37	nd	n/a	HIV SMART-xGen	51,448	38,288	74.42	77	524.27	1,835.32
9505343^‡^	Senegal	HIV-1 CRF06	nd	n/a	HIV SMART-xGen	99,834	37,446	37.51	91	484.82	945.49
814-43^†^	Cameroon	HIV-1 URF	nd	n/a	HIV SMART-xGen	485,500	438,989	90.42	93	5,872.10	11,973.02
770-8	Cameroon	HIV-1 URF	nd	n/a	HIV SMART-xGen	5,649,660	5,605,300	99.21	100	74,539.08	135,007.96
AB260^†^	Cameroon	HIV-1 group O	nd	n/a	HIV SMART-xGen	8,587,083	7,390,731	86.07	99	80,634.90	117,442.21
296	Cameroon	HIV-1 group O	nd	n/a	SSIII-xGen	17,777,038	17,243,456	97.00	100	220,976.08	354,818.32
108-08	Cameroon	HIV-1 group O	nd	n/a	SSIII-xGen	115,400	8,312	7.20	22	119.92	422.06
1095-04	Cameroon	HIV-1 group O	nd	n/a	SSIII-xGen	255,480	77,095	30.18	52	972.67	4,214.80
1225-51	Cameroon	HIV-1 group O	nd	n/a	SSIII-xGen	1,067,940	805,255	75.40	100	9,803.67	15,258.08
126-12	Cameroon	HIV-1 group O	nd	n/a	SSIII-xGen	284,826	46,947	16.48	56	463.94	1,313.88
20-02	Cameroon	HIV-1 group O	nd	n/a	SSIII-xGen	308,694	174,250	56.45	94	2,183.30	3,452.81
136-16	Cameroon	HIV-2 group B	nd	n/a	SSIII-xGen	6,068	2,922	48.15	75	36.03	47.93

† Genome completed (100%) with Sanger.

‡ *Genome coverage increased with Sanger but still incomplete*.

### Removal of contaminating sequences

To detect possible contaminating reads originating from a different set of barcodes, raw data from one sample was individually mapped to the consensus sequences of samples sequenced in the same run, requiring a similarity fraction of 0.99 (e.g., ≥99% identical). Mapped reads were removed from the fasta file. Unmapped reads (e.g., unique to the sample of interest) were collected and realigned to an appropriate reference to generate a consensus.

### Sequence gap closure

RT-PCR and Sanger (population) sequencing were performed as described to fill gaps in NGS sequence data (Rodgers et al., 2017a). Primer pairs used and their sequences are listed in the Supplemental Information. Complete genomes were obtained for 144-26, 363-24, PBS1342, AB260, and 814-43 by combining Sanger with NGS data. Additional sequence covering gaps was obtained for 9505343, 129-26, 961-09, 577-27, 112-11, and 8128965.

### Phylogenetic analysis

Alignments, neighbor-joining trees, and recombination analysis by SIMPLOT were all performed as described (Berg et al., 2016).

### Nucleotide sequence accession numbers

Open reading frames for all 28 full and 3 near complete genomes were verified and annotated using SeqBuilder (DNASTAR Lasergene v11.2) and submitted to GenBank as project 2135293 under the following accessions: PBS888 (MH705133), PBS1191 (MH705134), PBS1342 (MH705135), 459-16 (MH705136), 5056135 (MH705137), 8128965 (MH705138), 9505343 (MH705139), 814-43 (MH705140), 363-24 (MH705141), 770-8 (MH705142), 42-877 (MH705143), A1699 (MH705144), 144-26 (MH705145), 112-11 (MH705146), AB260 (MH705147), 296 (MH705148), O-1225-51 (MH705149), 20-02 (MH705150), 70641 (MH705151), PBS5635 (MH705152), PBS6126 (MH705153), 2049 (MH705154), P406 (MH705155), P3844 (MH705156), P4039 (MH705157), 2106 (MH705158), PBS0724 (MH705159), PBS1189 (MH705160), 50 (MH705161), 87-2580 (MH705162), PBS1195 (MH705163). Raw data depleted of human sequences can be found in the SRA database under BioProject ID: PJRNA486839.

## Results

### HIV-xGen target enrichment strategy

**Figure 1:**
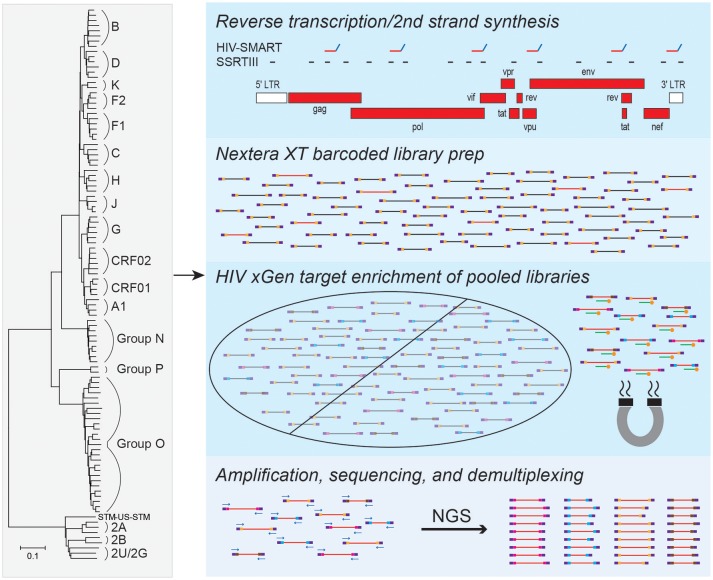
HIV-xGen strategy. 651 probes were selected to tile all HIV-1 and HIV-2 strains present in the phylogenetic tree at 1X coverage. Reverse transcription and second strand synthesis were performed by random priming with Superscript/Sequenase or by the HIV-SMART method. Nextera XT was used to convert cDNA to barcoded Illumina libraries consisting of both HIV (red inserts) and background (black) reads. Pooled libraries were hybridized to xGen probes (green) with 5′-biotin tags (gold) for a single capture and selected by magnetic bead separation. Multiplexed libraries were amplified by universal KAPA primers, sequenced on a MiSeq, and reads were parsed by barcode.

To enable full genome characterization of all HIV strains, we designed xGen probes to selectively capture and amplify viral sequences from cDNA libraries. Individual alignments were compiled for HIV-1 group M (subtypes A-K and circulating recombinant form [CRF] 02), groups N, O and P, as well as HIV-2 groups A and B. A minimum 80% identity has been shown to be required for effective xGen probe hybridization of viral sequences (Bonsall et al., 2015). Therefore, to eliminate redundancy and the synthesis of a prohibitively expensive number of probes, a consensus sequence from each group was generated from which an initial set of 120 nt single-stranded DNA probes tiling the genome at 1X coverage was derived. Alignments were then scanned in 100 nt windows to identify regions of strong nucleotide conservation and those of considerable heterogeneity. The minimum number of probes were selected to tile the former (e.g., >80% identity in *pol*), whereas those for heterogeneous regions (e.g., <80% identity in *env*) found in individual subtypes and strains were added as needed. Using this approach, only 183 probes were required for HIV-1 group M, compared to several thousand that would have been needed if probes were designed against the entire genomic sequence of individual strains. A total of 651 probes covered all HIV-1 and HIV-2 strains (Figure 1).

cDNA from HIV-infected plasma can be synthesized by either random (Superscript RTIII; SSRTIII) or virus sequence-specific priming (HIV-SMART; Figure 1), followed by topoisomerase-mediated fragmentation, adaptor tagging and amplification with Nextera XT (Berg et al., 2015, 2016). Previously, despite a 17–20-fold increase in sensitivity over metagenomic (random primed) libraries, together with additional optimization of the HIV-SMART protocol described here in section Materials and Methods to now consistently obtain full genomes from ≥log4 copies/ml samples, both library approaches on their own still yield a minority of viral sequences (1–5%; red inserts in Figure 1) relative to host and reagent background (black inserts in Figure 1; Luk et al., 2015; Berg et al., 2016). Using the probes described above, we explored whether target capture of HIV reads from these conventional libraries could boost NGS sensitivity for viral sequences present in low abundance (Figure 1).

Since xGen probes are modified with a 5′-biotin tag, Nextera XT adaptors lacking biotin needed to be synthesized to avoid streptavidin-mediated capture of all input sequences. Individually barcoded libraries were pooled together for a single capture, hybridized to HIV xGen probes on magnetic streptavidin beads, and washed to eliminate background (non-HIV) sequences. After PCR amplification, we note that the size range of resulting xGen libraries was often noticeably larger than their unenriched precursors (350–500 vs. 200–300 nt; data not shown). MiSeq runs were performed on an HIV-xGen super-library that typically multiplexed 6–26 samples, all with unique dual barcodes to permit parsing of data (Figure 1).

### Diverse sequences are captured and enriched in HIV-xGen libraries

**Figure 2:**
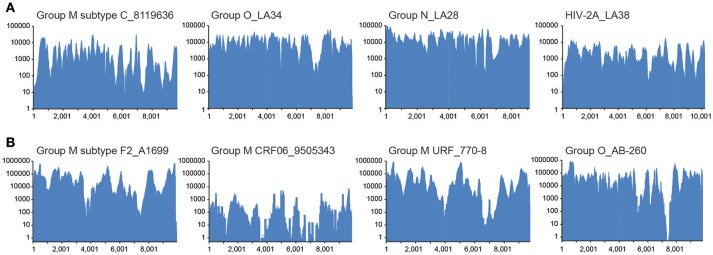
Diverse sequences are captured and enriched in HIV-xGen libraries. **(A)** Coverage plots of SSRTIII libraries enriched by HIV-xGen that were sequenced previously: HIV-1 group M subtype C (8119636), group O (LA34), group N (LA28), and HIV-2A (LA38). **(B)** Coverage plots of new HIV-1 strains libraries generated by HIV-SMART and followed by enrichment with HIV-xGen: HIV-1 group M subtype F2 (A1699), CRF06 (9505343), URF (770-8), and group O (AB260).

To establish that the expected range of diversity was indeed captured by this method and determine whether the yield of viral reads increased, HIV xGen was applied to a variety of HIV-1 and HIV-2 strains. We began with high-titer specimens or virus isolates we previously sequenced to assess the fidelity of probes and exclude the possibility that gaps in xGen-generated coverage were a result of reads missing from the Nextera starting material. Also, by remaking libraries and arriving at the same consensus, we could demonstrate that HIV-xGen was not introducing artifacts or sequence bias. For HIV-1 group M, a high titer subtype C strain from South Africa (8119636; log 5.62 copies/ml) previously sequenced by HIV-SMART was remade this time by random priming (SSRTIII) and once again yielded 98% coverage (Berg et al., 2016). Following a post-Nextera HIV-xGen selection, 100% coverage was obtained, implying that sufficiently complementary sequences were present among the probes (Figure 2; Table 1). Notably, whereas only 2.69% of metagenomics reads mapped to HIV, this improved dramatically to 98.23% with HIV-xGen selection. The resulting SSIII-derived HIV-xGen consensus sequence was 99.99% identical to the HIV-SMART sequence. We continued to evaluate the probe set for the ability to capture HIV-1 groups O and N and HIV-2A sequences from virus isolate-generated libraries. Complete coverage was previously obtained in each case, with HIV-SMART libraries comprised of 3.8% (LA34; group O) and 10.0% (LA28; group N) HIV reads and randomly primed Ovation Single Cell libraries with 1.33% (LA38; HIV-2A) HIV reads (Berg et al., 2016; Yamaguchi et al., 2017). Here, cDNA libraries of each isolate were remade by random priming in this study and once again, 100% coverage was achieved for all three strains with a post-Nextera HIV-xGen enrichment, confirming that probes adequately covering these diverse groups were present and functional. The percentage of HIV reads once again increased to 97.23% for LA34 (group O), 99.39% for LA28 (group N) and 96.89% for LA38 (HIV-2A; Figure 2A). Likewise, the consensus sequences derived from HIV-xGen were 100%, 99.99%, and 100% identical to prior sequences.

The HIV-xGen method was then applied to additional clinical samples from Cameroon and Senegal with either viral loads ≥log5 copies/ml or those with unknown titers, this time with HIV-SMART libraries as the starting cDNA. For HIV-1 group M, a subtype F2, a CRF06, and a unique recombinant (URF) were sequenced and 100%, 91%, and 100% coverage was obtained for each, respectively (Figure 2B; Table 2). An HIV-1 group O strain (O-AB260) also yielded 99% coverage. The percentages of HIV reads in the total ranged from 86 to 99%. Thus, a diverse set of high titer specimens were fully sequenced by HIV-xGen selection regardless of which cDNA synthesis method was deployed.

### HIV-xGen dramatically increases sensitivity for low titer specimens

**Figure 3:**
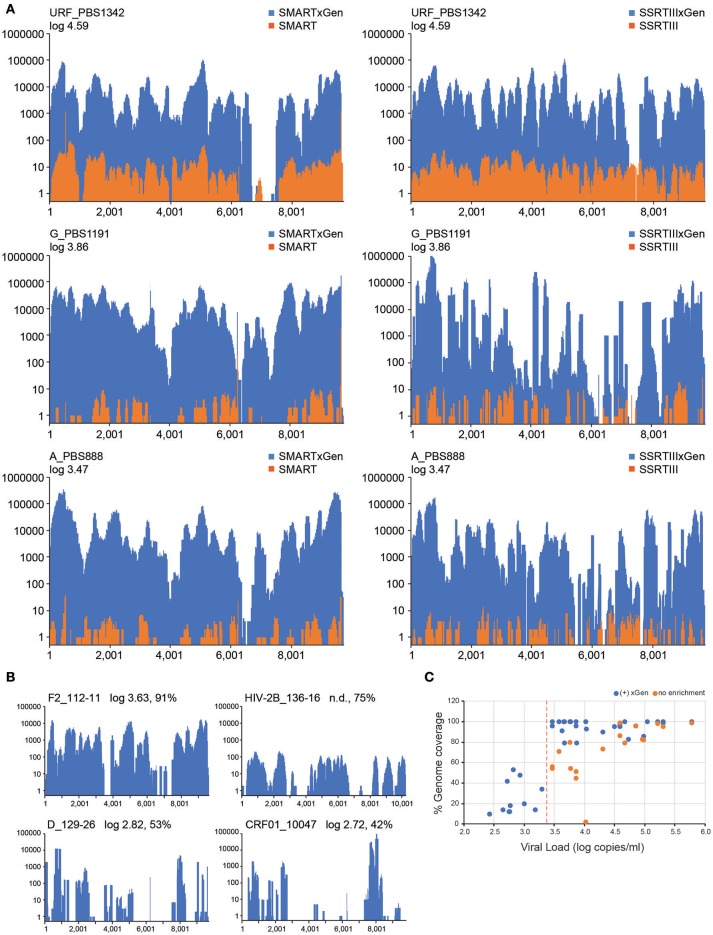
A HIV-xGen dramatically increases sensitivity for low titer specimens. **(A)** Coverage plots are shown for HIV-SMART libraries (top panels) without (orange) and with (blue) HIV-xGen as well as Superscript libraries without (orange) and with (blue) HIV-xGen. PBS1342 is a URF, PBS1191 is subtype G and PBS888 is subtype A. **(B)** Coverage plots for strains with titers ranging from log 2.7–3.6 copies/ml including a subtype F2 (112-11), a subtype D (129-26), a CRF01 (10047105267) and an HIV-2 strain (136-16) of unknown viral load. **(C)** Plot of viral load vs. genome coverage for all new strains sequenced with a known titer. Red dashed line = log 3.4 copies/ml; orange dots = no enrichment; blue dots = xGen enrichment.

The value of HIV-xGen will reside in its ability to fully sequence low titer specimens while multiplexing to the same or greater extent. Representative results from strains with viral loads of log 4.59 copies/ml (PBS1342; URF), log 3.86 copies/ml (PBS1191; subtype G) and log 3.47 copies/ml (PBS888; subtype A) demonstrate the dramatic improvements in coverage with enrichment compared to without (Figure 3A). For HIV SMART, genome coverage increases from 86, 51, and 56% without xGen to 95%, 100%, and 100% with xGen, respectively. Similarly, genome coverage with Superscript (SSRTIII) changes from 99, 45, and 54% without xGen to 97, 96, and 96% with xGen, respectively. Here again, HIV-xGen libraries were comprised almost entirely (90–99%) of HIV sequence regardless of the cDNA synthesis method chosen (SMART or SSRTIII) and resulting consensus sequences were 99.51, 99.04, and 99.76% identical, respectively.

Thirteen additional samples from the Democratic Republic of Congo, ranging in titers of log 3.58 to 5.78 copies/ml, were sequenced here by the HIV-SMART ± xGen method (Table 1). These specimens together with the examples above reveal a median 1,147x (range 24.7–56, 509x) boost in HIV read yield upon xGen enrichment. For high titer specimens, percent genome coverage is largely unaffected (see below Figure 3C), although the depth of coverage is substantially increased as the same reads are re-sequenced. However, specimens <log 4.5 copies/ml saw both a significant increase in depth and overall genome coverage, indicating that additional HIV reads are present in libraries which have not been sequenced without enrichment (Table 1, Figure 3C). In a few instances, fewer than 10 reads were initially mapped, which following xGen selection, resulted in 97–100% coverage (subtype K, P3844; subtype A1, PBS0724). The average increase in percent genome coverage for xGen-enriched compared to unenriched samples was 40.5%. Indeed, all samples >log 3.5 copies/ml had ≥79% coverage, with the majority (72%) of these having >95% genome coverage. Once again, strain consensus sequences were virtually identical independent of xGen, as well as when PCR duplicate reads were removed during mapping (Supplemental Table S2A). Similarly, the total number of minor variants (MV; 10–50%) detected was consistent between methods. However, while PCR duplicate removal had minimal effect on the extent to which the exact same MVs were detected (70–100% overlap), this overlap was reduced when comparing (-)xGen to (+)xGen datasets (28–84% overlap; Supplemental Table S2B).

We next explored strains with viral loads ranging from log 2.6–3.6 copies/ml, continuing with the HIV-SMART + HIV xGen approach (Table 2). Coverage plots for xGen-enriched libraries in Figure 3B illustrate that while full genomes are not possible with this method in this titer range, the partial coverage obtained for some can still be substantial (e.g., 40–80%). Once again, sequences from different geographies were successfully captured, including a subtype F2 (112-11, Cameroon), D (129-26; Cameroon), and CRF01 (10047105267; Thailand). As testament to the method sensitivity, an HIV-2 strain (136-16; Cameroon), which typically replicates at low titers, was extracted from diluted patient plasma and attained 75% genome coverage (Figure 3B). The average percent genome sequenced for those with ≤log 3 copies/ml is 26 ± 18%. For two samples at log 3.24 and 3.26 copies/ml, zero HIV reads were obtained. It is noteworthy that input for cDNA synthesis in this range is <200 copies of virus and that for many of these samples, simply obtaining a product by RT-PCR was also a challenge.

To summarize the results for all samples attempted in which a titer was known, genome coverage was plotted against viral load. Most samples >log 3.5 copies/ml achieved near-complete coverage and those below this threshold yielded a partial genome (Figure 3C). Different colors (±xGen) for the same samples (identical viral loads) illustrate the significant jumps in coverage following enrichment. There were several strains for which low sample volume precluded viral load testing, particularly for HIV-1 group O and rare circulating recombinants (Table 2). For more than half of these we succeeded in obtaining >90% of the genome.

### HIV xGen facilitates classification and characterization of diverse HIV strains

**Figure 4:**
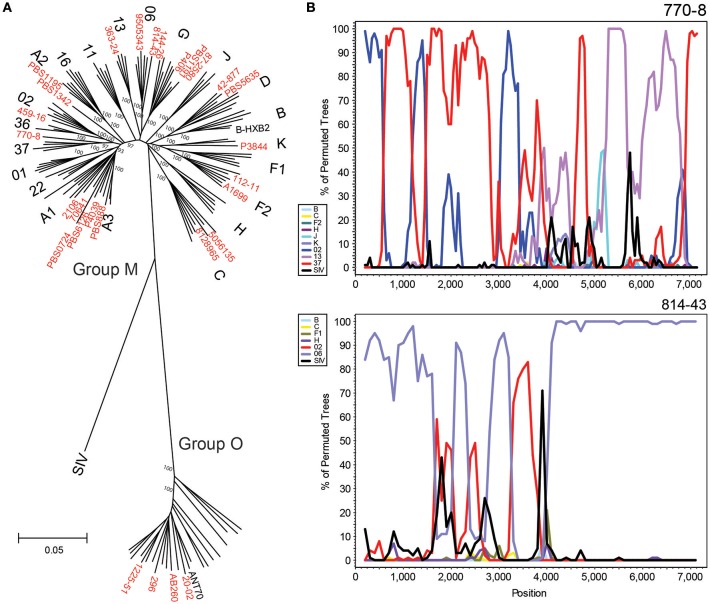
HIV-xGen enables full genome characterization of rare and diverse strains. **(A)** Neighbor joining phylogenetic tree of 28 new full genomes (red) generated from a 6,252 nt gap-stripped alignment. Specimens sequenced in this study are shown in red. **(B)** Boot-scanning analysis of unique recombinant forms 770-80 and 814-43.

A total of 50 new strains originating from the Democratic Republic of Congo, Cameroon, Thailand, South Africa, Senegal, and Uganda were sequenced by HIV-xGen. Phylogenetic classification of 28 complete or near-complete (>90%) genomes determined in this study are shown using a 6,252 nt gap-stripped alignment (Figure 4A). Many of the major subtypes and CRFs are represented here, as well as four group O strains, illustrating the breadth of viral diversity captured by this method. Phylogenetic classifications of partial genome sequences with 75–90% (Supplemental Figure S1) and <75% (Supplemental Figure S2) coverage are found in the Supplemental Information.

Strains branching basal to Group M subtype nodes were investigated further by Simplot and boot-scanning to reveal evidence of recombination. For each initial Simplot analysis preceding the final bootscan shown, appropriate references were included to verify that the strain in question was more similar to the recombinant sequence than to the pure reference sequence of the same subtype (e.g., A, G, etc.). Sub-genomic RT-PCR of *env* immunodominant region (IDR) originally categorized 770-8 as a CRF13. While NGS confirmed the classification of this portion of the sequence, we were able to determine with the full genome that it was actually a unique recombinant form consisting of CRF02, CRF37 and CRF13 sequences. Similarly, full genome characterization of 814-43 demonstrated it was not simply a CRF06, but rather a URF consisting of CRF06 and CRF02 sequence (Figure 4B). Plotting consensus base call percentages at each position ruled out dual or super-infections, since aside from the occasional minor variant, values approached 100% throughout the genome. No continuous stretches of lower consensus base call percentages were observed, indicating only one major recombinant species was present (data not shown).

## Discussion

HIV-xGen is a universal, robust, and cost-effective back-end to any cDNA method deployed for next generation sequencing of HIV-1 and HIV-2. Previously, with benzonase-treated extractions and either our optimized gene-specific (HIV-SMART) or standard random priming approaches for cDNA synthesis, we were still challenged by sensitivity. While we obtained much greater coverage for samples with titers between log 4 and log 5 copies/ml, genomes were still incomplete (Berg et al., 2016; Rodgers et al., 2017a). Now, with HIV-xGen, we can routinely obtain full genomes at a lower limit of log 3.5 copies/ml, whereas previously without enrichment, samples in the log 3.5–4.5 range would only yield 20–50% coverage. Thus, it was clear that these HIV reads were actually present in libraries, but we were not sequencing to a sufficient read depth to observe them. Below this log 3.5 threshold, the ability to adequately sequence samples is likely a limitation of cDNA synthesis, and not xGen; it cannot capture and amplify material that was never reverse transcribed. With specimens each having inherent differences in host background, detection is not linear in this range. Some may obtain >50% coverage whereas others of similar titer recover no sequence at all. Overall, our results are consistent with what others have reported for probe-mediated positive selection of viral sequences (Bonsall et al., 2015; Briese et al., 2015). Simply sequencing deeper, on a higher throughput instrument (e.g., HiSeq vs. MiSeq), or multiplexing less could certainly provide improved detection and coverage of low titer samples, but the overall read proportions would likely remain the same. Now with xGen, far more samples can be processed and sequenced at once as a greater percentage of reads are viral, saving time and resources. Throughput and NGS capacity are further increased by virtually eliminating the sequencing of host background.

As with HIV-SMART, the objective with HIV-xGen was to fully characterize strains for the purpose of surveillance. With the sensitivity we now demonstrate, the number of specimens previously deemed too challenging to sequence by NGS due to low titer has markedly declined. As a greater proportion of patients are on therapy and able to suppress viral loads, it is imperative that our methods can adequately characterize low titer specimens. While we only attempted to multiplex a maximum of 26 samples at a time, the high percentage of HIV reads from the total per barcode suggests many more libraries (e.g., an entire 96-well plate) could be pooled without detriment. Considering the depth of coverage now possible, assessing levels of minor variants and quasi-species in samples could be an attractive application for HIV-xGen. For example, detection of minor variants at clinically relevant levels (e.g., >10%) to predict the emergence of drug resistance and treatment failure should be readily achievable with this method (Li and Kuritzkes, 2013; Obermeier et al., 2014; Noguera-Julian et al., 2017). However, primer IDs controlling for starting cDNA populations and potential PCR bias were not used in this study and the original proportions of minor variants after Nextera amplification might be expected to drift further after additional rounds of HIV-xGen amplification (Jabara et al., 2011; Boltz et al., 2016). Indeed, we showed that while consensus sequences do not change with enrichment or inclusion of duplicate reads, minor variant populations are affected by xGen (Supplemental Table S2). Nevertheless, our primary goal was to not limit interrogation to sub-genomic regions via amplicon sequencing, but rather to better comprehend the complete extent of diversity in entire genomes found in different geographic regions at different times. As an example, our prior surveillance efforts in Cameroon have consistently observed a predominance of CRF02, CRF06, CRF13, and CRF37 (Rodgers et al., 2017b). Now it appears that the URFs we are detecting in this region are recombinants and contain genome segments that do not phylogenetically cluster with homologous sequences derived from any of the classified HIV-1M (Figure 4).

With the batching of samples during hybridization and amplification steps, the inability to control for the resulting proportion of reads/barcode is one shortcoming of the method. Generally, the resulting read numbers were a reflection of the starting viral loads. Thus, we recommend, if possible, that samples be grouped by titer to avoid an imbalance. Nevertheless, given the potential for one or more samples to predominate in a run relative to the others in the pool, cross-contamination of reads between barcodes on the MiSeq was of concern (Lee et al., 2016). Therefore, raw data from each sample was mapped to each individual HIV xGen-derived consensus sequence of other samples in the same run to identify regions of perfect identity which might indicate the incorrect binning of reads. Fortunately, this was infrequently observed, with most errant reads derived from high read depth samples, and particularly in those samples where a barcode may have been shared. Thus, we further recommend that unique dual barcode pairs be chosen for each library in a pool. Here, reads clearly originating from another sample were removed from the dataset and the mapping was repeated to derive an accurate consensus. In most cases, we had prior Sanger data to compare the NGS consensus sequences against to verify that the final sequence obtained was correct.

Another shortcoming of the method is that the full scope of HIV diversity cannot be known, therefore probes comprehensive of all sequences cannot be designed, and yet this is an argument in favor of continued surveillance. As an example in Figure 3A, PBS1342 exhibits a gap in the *env* region using either cDNA synthesis approach. The HIV-SMART reverse primer binding site is considerably downstream of the gap and fails to explain the absence of sequence, irrespective of enrichment. In contrast, randomly primed libraries did sequence successfully across this region, for which only a small portion (nt 7,196–7,455) was not recovered by xGen. Comparison of the 120 nt probes spanning this region to the PBS1342 consensus sequence revealed an overall lower identity (78–85%), but presumably the concentration of mismatches and indels we observed were the major factor. In the first probe, 14 of the total 18 mismatches were focused in the 3′ 50 nucleotides, the middle probe had two insertions (6, 3 nt), one deletion (3 nt), and 13 mismatches all situated in the 5′ 53 nucleotides, and in the third there were 19 mismatches over a stretch of 33 nucleotides, preceded by two insertions (6, 27 nt) and a deletion (15 nt). Adequate coverage of envelope will likely remain a challenge, particularly as we explore geographies with high sequence diversity such as the DRC, yet the tolerance for mismatches displayed throughout the genomes of numerous strains from HIV-1 and HIV-2 groups speaks to the robustness of the method.

It is essential that diagnostic tests keep pace with HIV and other rapidly mutating viruses by proactively seeking out strains in circulation that may escape detection with current assays (Brennan et al., 2006). The ability to fully characterize multiple samples simultaneously without regard for subtype, group, or titer opens up greater opportunities for future surveillance. At the same time, using HIV-xGen to retrospectively examine archived specimens and understand the origins of the HIV epidemics is an equally compelling application (Rodgers et al., 2017a). Metagenomic NGS combined with selective viral sequencing promise a new era in diagnostic virology (Barzon et al., 2011; Quiñones-Mateu et al., 2014; Kumar et al., 2017). As sensitivity and throughput increases with methods like HIV-xGen, we are one step closer to realizing this potential.

## Author contributions

JY and MB conceived of methodology and designed HIV-xGen probes. JY, AO, and KF performed experiments and data analysis. MR performed data analysis, assisted with experimental design, and reviewed manuscript. MB performed data analysis, directed experimental design, wrote manuscript and made figures. GC assisted experimental design and reviewed manuscript. TQ and OL collected and characterized DRC samples and reviewed manuscript. NN, DM, and LK collected and characterized Cameroon samples and reviewed manuscript.

### Conflict of interest statement

JY, MR, AO, KF, GC, and MB are all Abbott employees and shareholders. The remaining authors declare that the research was conducted in the absence of any commercial or financial relationships that could be construed as a potential conflict of interest.
