# Supplementary material for: Universal Target Capture of HIV Sequences From NGS Libraries
Source: Front Microbiol. 2018 Sep 13;9:2150. doi: 10.3389/fmicb.2018.02150 (PMC6146096; doi:10.3389/fmicb.2018.02150)
Supplement: Supplementary file 1 [file Data_Sheet_1.PDF]

Yamaguchi, et al. Online Supplemental Information

Materials and Methods

Genome sequence completion Gaps in NGS-generated genomes were filled by RT-PCR and Sanger sequencing for the following 6 samples. Values in Tables 1 and 2 of the main text reflect coverage before addition of these sequences. Those shown below are indicated accordingly: "†Genome completed (100%) with Sanger; ‡ Genome coverage increased with Sanger but still incomplete." Primers and their sequences are listed below.

Sample ID	gap positon	gap size	primer pair
PBS1342	6643-6649	7 bp	M6577F-JH45R
PBS1342	7410-7481	72 bp	M6823F-JH35m
AB260	5155-5168	14 bp	Opoli12F-env3R
AB260	5322-5324	3 bp	Opoli12F-env3R
AB260	5396-5425	30 bp	Opoli12F-env3R
AB260	5756-5782	27 bp	Opoli12F-env3R
AB260	6685-6717	33 bp	env1F-Oligo dT
AB260	7326-7420	95 bp	env1F-Oligo dT
144-26	3511-3545	35 bp	M3345F-M3772R
144-26	3916-3954	39 bp	55RT3214F-MHApol2R
144-26	6253-6389	137 bp	MN6217F-55EN7052R
144-26	6643-6798	156 bp	MN6217F-55EN7052R
144-26	6977-7432	456 bp	MN6217F-55EN7052R
5056135	6626-6690	65 bp	M6577F-M6895R
814-43	2181-2195	15 bp	gagseq1027F-M2353R
814-43	6649-7342	694 bp	M6577F-JH45R
363-24	3532-3678	147 bp	M3345F-MHApol-6R
363-24	3943-4119	177 bp	M3345F-MHApol-6R
363-24	4263-4275	13 bp	M3345F-MHApol-6R
363-24	5239-5666	428 bp	pol4565F-55TA5518R
363-24	6020-6866	847 bp	M5967F-J6203R
363-24	7129-7474	346 bp	J6265F-JH35m
PBS5635	6288-6397	109 bp	CGgapFWD5-CGgapREV5
PBS1189	868-1024	156 bp	5LTRps46f-LPgag6R
PBS1189	5349-5412	63 bp	pol4565f-55env272r
PBS1189	6246-6752	506 bp	JH09-M6895R

PBS1189	7081-7123	42 bp	M6874F-JH35m
PBS1189	7391-7423	32 bp	M6874F-JH35m
50	1013-1294	281 bp	5LTRps44f-Mp24-1r
50	3518-3651	133 bp	M3345F-Opol25r
50	5490-5814	324 bp	CGgapFWD25-CGgapREV17
50	6120-6786	666 bp	CGgapFWD10-CGgapREV10
50	6120-6786	666 bp	CGgapFWD10-CGgapREV10
50	7768-7925	157 bp	JH35F-IDRps38r
50	8336-8385	49 bp	CGgapFWD11-CGgapREV11
50	8612-8642	30 bp	CGgapFWD12-CGgapREV12
87-2580	6754-6829	75 bp	CGgapFWD17-CGgapREV16
87-2580	7115-7315	200 bp	CGgapFWD17-CGgapREV16
87-2580	8378-8518	140 bp	CGgapFWD4-CGgapREV4
2049	3749-3923	174 bp	M3345F-rt-inseqR2
PBS0724	6233-6875	642 bp	CGgapFWD13-CGgapREV13
PBS0724	7019-7617	598 bp	CGgapFWD13-CGgapREV13

Name	Length	Sequence	Location B-HXB2 or O-ANT70
55EN7052R	28	TCCTGCRATGGGAGGGGCATAYATTGCT	7520<7547
55RT3214F	24	CAGARAGCATARTAATATGGGGAA	3679>3702
55TA5518R	26	GCTTCTTCCTGCCATAGGAGATGCCT	5959<5984
env1F	19	CCARAGCAGTAAGTAACGC	6089>6107 (ANT70)
env3R	18	GTTARCATCTGARGCACA	6449<6431 (ANT70)
gagseq1027F	21	GCTWCAYTAGAAGAAATGATG	1816>1836
J6203R	26	GGRGCACAATAATGTATRGGAATTGG	6858<6883
J6265F	29	AATGTCAGYACAGTACAATGYACACAYGG	6945>6973
JH35m	21	CACTTCTCCAATTGTCCITCA	7648<7668
JH45R	22	CARTAGAAAAATTCYCCTCYAC	7357<7378
M2353R	25	TCTAATACTGTATCATCTGCTCCTG	2329<2353
M3345F	23	TGGGCAAGTCAGATTTATCCAGG	3345>3367
M3772R	22	GCTTGCCAATAGTCTGTCCACC	3751<3772
M5967F	21	CCTATGGCAGGAAGAAGCGGA	5967>5987
M6577F	25	CATGTGTAAAGTTAACCCCWCTCTG	6577>6601
M6823F	24	CCATTAAACAGGCTTGTCCAAAGG	6823>6846
M6895R	22	GCAAAACCAGCTGGRGCACAAT	6874<6895
MHApol-2R	26	TGCCATATYCCTGGACTRCAGTCTAC	4389<4414
MHApol-6R	26	TTTGCTACTATAGGTGGCAGATTAAA	4305<4330
MN6217F	28	GAAAGAGCAGAAGACAGTGGCAATGAG	6203>6229

Opoli12F	21	AGGGAGCAGTAGTCATACAAG	5021>5041 (ANT70)
pol4565F	24	TATGGAAAACAGATGGCAGGTGAT	5040>5063
55env272r	24	TCTGGGGCTTGTTCCATCTATCTT	5552<5575
5LTRps44f	20	AGGCCAGGGGGAAAGAAAAG	853>872
5LTRps46f	20	GCTAGAAGGAGAGAGATGGG	775>794
CGgapFWD10	18	GGAACCAGCCAGGAAGTC	5862>5879
CGgapFWD11	21	ACCTGGCTGCAATGGGACAAA	8103>8123
CGgapFWD12	20	AGCCTGTGGACTTGGTTTGA	8226>8245
CGgapFWD13	22	AAGCGGAAACGACGACGAAAAA	5981>6002
CGgapFWD17	24	ACCCCAACCCACAAGAGATAACTA	6457>6479
CGgapFWD25	26	AGCAACAGACATACAAACTAAAGAAC	4841>4866
CGgapFWD4	18	CTAATGTGCCTTGGAACG	8041>8058
CGgapFWD5	24	TGCTCCTTGGAATGTTGATGACCT	6283>6306
CGgapREV10	24	ATTTAGCAGCAGTTGAGTTGATAC	6987<7010
CGgapREV11	17	CTCTCAAGCGGTGGTAG	8525<8541
CGgapREV12	22	TCCTTTCTGCGCCCTGTCTGAT	8763<8784
CGgapREV13	21	CCCAAGAACCCAAGGAACACA	7775<7795
CGgapREV16	24	CCACTCTCTGCCACATTCTCACAA	7492<7515
CGgapREV17	22	TCTCCGCTTCTTCCTGCCATAG	5968<5989
CGgapREV4	19	GCTACTGCTATTGCTGTGG	8674<8692
CGgapREV5	24	CAGCAGCAGTTGAGTTGACACCAC	6984<7007
IDRps38r	22	TTCTTTTCTTGCTGGTTCTGCG	8170<8191
JH09	30	TTAGGCATNTCCTATGGCAGGAAGAAGCGG	5957>5986
JH35F	22	TGARGGACAATTGGAGAARTGA	7648>7669
LPgag6R	24	AACATGGGTATTACTTCTGGGCTG	1281<1304
M6874F	22	ATTGTGCYCCAGCTGGTTTTGC	6874>6895
Mp24-1r	20	TCTCCYACTGGRAYAGGTGG	1549<1568
Opol25r	20	CATTCWGGAATCCAGGTGGC	3771<3790
rt-inseqR2	26	GAAGTAAAYATAGTAACAGACYCACA	4023<4048

Supplemental Figure Legends

Supplemental Figure S1. Phylogenetic analysis of incomplete genomes. Individual neighbor joining phylogenetic trees of 7 new strains (red) with 75-90% genome coverage. Resulting length of alignment following gap-stripping is indicated for each.

Supplemental Figure S2. Phylogenetic analysis of incomplete genomes. Individual neighbor joining phylogenetic trees of 8 new strains (red) with <75% genome coverage. Resulting length of alignment following gap-stripping is indicated for each.

Supplemental Table Legends

Supplemental Table S1. HIV-xGen probes. Probe IDs and sequences (n=652) are listed along with genomic coordinates relative to consensus group reference or individual subtype consensus sequences.

Supplemental Table S2A. The consensus sequence identity was compared for 5 HIV-SMART datasets from Table 1 with matched xGen results. Exact PCR duplicates were removed from each dataset and a sequence identity matrix was performed. Total mapped reads and percent identity values between resulting consensus sequences are reported.

Supplemental Table S2B. Minor variant (MV) analysis was performed on the same 5 HIV-SMART datasets in **Table S2A**, once again with exact PCR duplicates removed from SMART and SMART-xGen datasets. Percent genome coverage and total MV >10% detected are listed. The datasets were then compared to each other over shared regions of coverage to determine the overlap in exact MV detected (e.g. sample genomic coordinate). Each cell lists the number of identical MV, followed by the percentage of this overlap relative to each dataset.

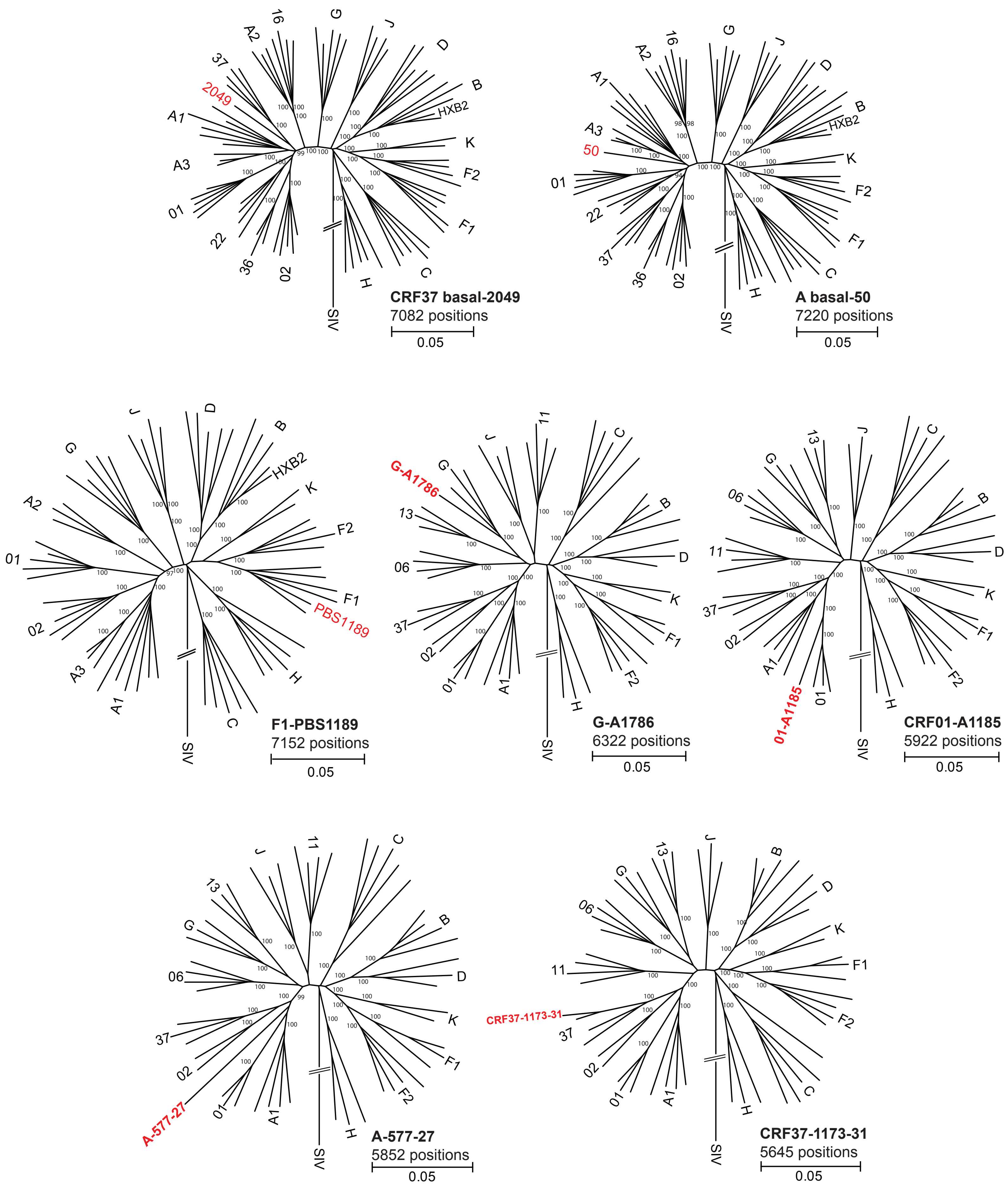

Yamaguchi, et al Supplemental Figure S1

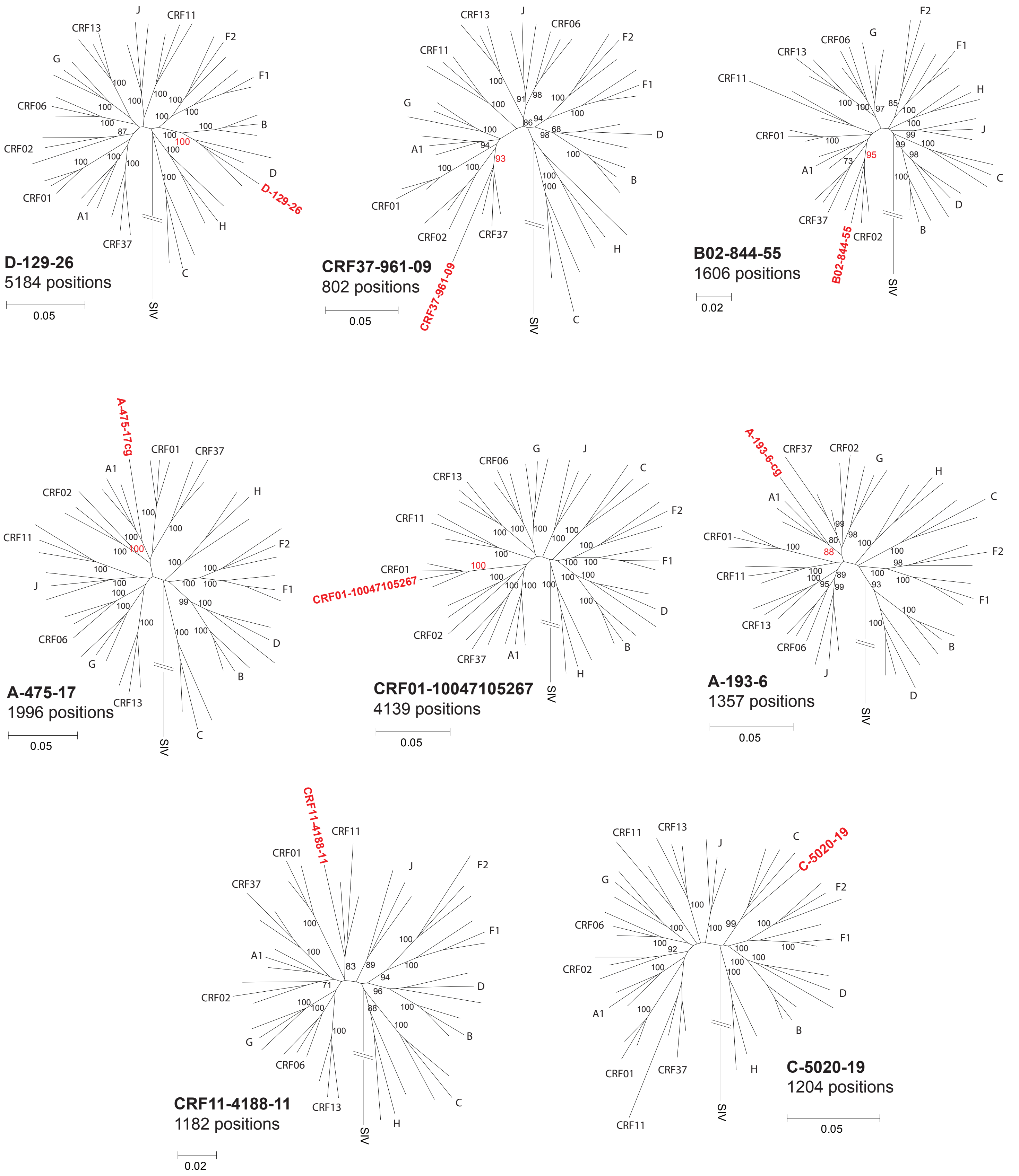

Yamaguchi, et al Supplemental Figure S2

Supplemental Table S1. HIV-xGen Probes

SequenceName	Start	Stop	Sequence
176861_8776076_HIV1-Con_M__1_1	0	120	TGGAAGGGCTAATTTACTCCCAGAAAAGACAAGAGATCCTTGATCTGTGGGTCTACCACACACAAGGCTACTTCCCTGATTGGCAGAACTACACACCAGGGCCAGGGATCAGATATCCAC
176861_8776076_HIV1-Con_M__1_2	119	239	CTGACCTTTGGATGGTGCTTCAAGCTAGTACCAGTTGACCCAGAGGAGATAGAAGAGGCCAATGAAGGAGAGAACAACTGCTTGTTACACCCTATGAGCCAGCATGGAATGGATGACCCG
176861_8776076_HIV1-Con_M__1_3	238	358	GGAGAAAGAAGTGTTAGTGTGGAAGTTTGACAGCCGCCTAGCATTTCATCACATGGCCCGAGAGCTGCATCCGGAGTACTACAAAGACTGCTGACATCGAGCTTTCTACAAGGGACTTTC
176861_8776076_HIV1-Con_M__1_4	357	477	CCGCTGGGGACTTTCCAGGGGAGGCGTGGCCTGGGCGGGACTGGGGAGTGGCTAACCCTCAGATGCTGCATATAAGCAGCTGCTTTTCGCCTGTACTGGGTCTCTCTGGTTAGACCAGAT
176861_8776076_HIV1-Con_M__1_5	476	596	TCTGAGCCTGGGAGCTCTCTGGCTAGCTAGGGAACCCACTGCTTAAGCCTCAATAAAGCTTGCCTTGAGTGCTTCAAGTAGTGTGTGCCCGTCTGTTGTGTGACTCTGGTAACTAGAGAT
176861_8776076_HIV1-Con_M__1_6	595	715	TCCCTCAGACCCTTTTAGTCAGTGTGGAAAATCTCTAGCAGTGGCGCCCGAACAGGGACTTGAAAGCGAAAGTAAAACCAGAGAAGATCTCTCGACGCAGGACTCGGCTTGCTGAAGTGC
176861_8776076_HIV1-Con_M__1_7	714	834	CACACGGCAAGAGGCGAGAGGCGGCGACTGGTGAGTACGCCAAATTTTTGACTAGCGGAGGCTAGAAGGAGAGAGATGGGTGCGAGAGCGTCAGTATTAAGCGGGGGAAAATTAGATGCA
176861_8776076_HIV1-Con_M__1_8	833	953	ATGGGAAAAAATTCGGTTAAGGCCAGGGGGAAAGAAAAAATATAGACTAAAACATCTAGTATGGGCAAGCAGGGAGCTGGAAAGATTTGCACTTAACCCTGGCCTTTTAGAAACATCAGA
176861_8776076_HIV1-Con_M__1_9	952	1072	AAGGCTGTAAACAAATAATAGGACAGCTACAACCAGCTCTTCAGACAGGATCAGAAGAACTTAGATCATTATATAATACAGTAGCAACCCTCTATTGTGTACATCAAAGGATAGAGGTAA
176861_8776076_HIV1-Con_M__1_10	1071	1191	AAAGACACCAAGGAAGCTTTAGATAAGATAGAGGAAGAACAAAACAAAAGTCAGCAAAAAACACAGCAAGCAGCAGCTGACAAAGGAAACAGCAGCCAGGTCAGTCAAAATTACCCTATA
176861_8776076_HIV1-Con_M__1_11	1190	1310	AGTGCAGAATCTACAAGGGCAAATGGTACACCAGGCCATATCACCTAGAACTTTAAATGCATGGGTAAAAGTAATAGAAGAGAAGGCTTTCAGCCCAGAAGTAATACCCATGTTTTCAGC
176861_8776076_HIV1-Con_M__1_12	1309	1429	CATTATCAGAAGGAGCCACCCCACAAGATTTAAACACCATGCTAAACACAGTGGGGGGACATCAAGCAGCCATGCAAATGTTAAAAGATACCATCAATGAGGAAGCTGCAGAATGGGACA
176861_8776076_HIV1-Con_M__1_13	1428	1548	AGGTTACATCCAGTGCATGCAGGGCCTATTGCACCAGGCCAGATGAGAGAACCAAGGGGAAGTGATATAGCAGGAACTACTAGTACCCTTCAGGAACAAATAGCATGGATGACAAGCAAC
176861_8776076_HIV1-Con_M__1_14	1547	1667	CCCACCTATCCCAGTGGGAGAAATCTATAAAAGATGGATAATCCTGGGATTAAATAAAATAGTAAGAATGTATAGCCCTGTCAGCATTTTGGACATAAGACAAGGGCCAAAAGAACCCTT
176861_8776076_HIV1-Con_M__1_15	1666	1786	TTAGAGACTATGTAGACAGGTTCTTTAAAACTCTAAGAGCTGAGCAAGCTACACAGGAAGTAAAAAATTGGATGACAGACACCTTGTTGGTCCAAAATGCGAACCCAGATTGTAAGACCA
176861_8776076_HIV1-Con_M__1_16	1785	1905	ATTTTAAAAGCATTAGGACCAGGGGCTACACTAGAAGAAATGATGACAGCATGTCAGGGAGTGGGAGGACCCGGCCATAAAGCAAGAGTTTTGGCTGAGGCAATGAGCCAAGCAACAAAT
176861_8776076_HIV1-Con_M__1_17	1904	2024	TACAGCTACCATAATGATGCAGAGAGGCAATTTTAAGGGCCAAAGAAGAATTGTTAAGTGTTTCAACTGTGGCAAAGAAGGACACATAGCCAGAAATTGCAGGGCCCCTAGGAAAAAGGG
176861_8776076_HIV1-Con_M__1_18	2023	2143	GCTGTTGGAAATGTGGAAAGGAAGGACACCAAATGAAAGACTGCACTGAGAGACAGGCTAATTTTTTAGGGAAAATTTGGCCTTCCAACAAGGGGAGGCCAGGGAATTTTCTTCAGAGCA
176861_8776076_HIV1-Con_M__1_19	2142	2262	AGACCAGAGCCAACAGCCCCACCAGCAGAGAGCTTCGGGTTCGGGGAGGAGATAACCCCCTCTCCGAAGCAGGAGCAGAAAGACAAGGAACTGTATCCCTTAACTTCCCTCAAATCACTC
176861_8776076_HIV1-Con_M__1_20	2261	2381	CTTTGGCAACGACCCCTTGTCACAATAAAAATAGGGGGACAGCTAAAGGAAGCTCTATTAGATACAGGAGCAGATGATACAGTATTAGAAGACATAAATTTGCCAGGAAAATGGAAACCA
176861_8776076_HIV1-Con_M__1_21	2380	2500	AAAAATGATAGGGGGAATTGGAGGTTTTATCAAAGTAAGACAGTATGATCAAATACTCATAGAAATTTGTGGAAAAAAGGCTATAGGTACAGTATTAGTAGGACCTACACCTGTCAACAT
176861_8776076_HIV1-Con_M__1_22	2499	2619	TAATTGGAAGAAATATGTTGACTCAGATTGGTTGCACTTTAAATTTTCCAATTAGTCCTATTGAAACTGTACCAGTAAAATTAAAGCCAGGAATGGATGGCCCAAAGGTTAAACAATGGC
176861_8776076_HIV1-Con_M__1_23	2618	2738	CCATTGACAGAAGAAAAAATAAAAGCATTAACAGAAATTTGTACAGAAATGGAAAAGGAAGGAAAAATTTCAAAAATTGGGCCTGAAAATCCATACAATACTCCAGTATTTGCCATAAAG
176861_8776076_HIV1-Con_M__1_24	2737	2857	GAAAAAAGACAGTACTAAATGGAGAAAATTAGTAGATTTCAGAGAACTTAATAAAAGAACTCAAGACTTCTGGGAAGTTCAATTAGGAATACCACATCCAGCAGGGTTAAAAAAGAAAAA
176861_8776076_HIV1-Con_M__1_25	2856	2976	AATCAGTAACAGTACTGGATGTGGGGGATGCATATTTTTCAGTTCCCTTAGATGAAGACTTCAGGAAGTATACTGCATTCACCATACCTAGTATAAACAATGAGACACCAGGGATTAGAT
176861_8776076_HIV1-Con_M__1_26	2975	3095	TATCAGTACAATGTGCTTCCACAGGGATGGAAAGGATCACCAGCAATATTCCAAAGTAGCATGACAAAAATCTTAGAGCCCTTTAGAACAAAAAATCCAGAAATAGTTATCTATCAATAC
176861_8776076_HIV1-Con_M__1_27	3094	3214	CATGGATGATTTGTATGTAGGATCTGACTTAGAAATAGGGCAGCATAGAGCAAAAATAGAGGAGTTAAGAGAACATCTATTGAAGTGGGGATTTACCACACCAGACAAAAAACATCAGAA
176861_8776076_HIV1-Con_M__1_28	3213	3333	AAGAACCTCCATTTCTTTGGATGGGGTATGAACTCCATCCTGACAAATGGACAGTACAGCCTATACAGCTGCCAGAAAAGGACAGCTGGACTGTCAATGATATACAGAAGTTAGTGGGAA
176861_8776076_HIV1-Con_M__1_29	3332	3452	AAATTAAATTGGGCAAGTCAGATTTATCCAGGGATTAAAGTAAAGCAATTATGTAAACTCCTTAGGGGAGCCAAAGCACTAACAGACATAGTACCACTGACTGAAGAAGCAGAATTAGAA
176861_8776076_HIV1-Con_M__1_30	3451	3571	ATTGGCAGAGAACAGGGAAATTCTAAAAGAACCAGTACATGGAGTATATTATGACCCATCAAAAGACTTAATAGCAGAAATACAGAAACAAGGGCAAGACCAATGGACATATCAAATTTA
176861_8776076_HIV1-Con_M__1_31	3570	3690	ATCAAGAGCCATTTAAAAATCTGAAAACAGGAAAGTATGCAAAAATGAGGTCTGCCCACACTAATGATGTAAAACAATTAACAGAGGCAGTGCAAAAAATAGCCACAGAAAGCATAGTAA
176861_8776076_HIV1-Con_M__1_32	3689	3809	ATATGGGGAAAGACTCCTAAATTTAGACTACCCATACAAAAAGAAACATGGGAAACATGGTGGACAGAGTATTGGCAAGCCACCTGGATTCCTGAGTGGGAGTTTGTCAATACCCCTCCT
176861_8776076_HIV1-Con_M__1_33	3808	3928	TCTAGTAAAATTATGGTACCAGTTAGAAAAAGAACCCATAGTAGGAGCAGAAACTTTCTATGTAGATGGGGCAGCTAATAGGGAGACTAAAATAGGAAAAGCAGGATATGTTACTGACAG
176861_8776076_HIV1-Con_M__1_34	3927	4047	GAGGAAGACAAAAAGTTGTCTCCCTAACTGAAACAACAAATCAGAAGACTGAATTACAAGCAATTCATCTAGCTTTGCAGGATTCAGGATCAGAAGTAAACATAGTAACAGACTCACAGT
176861_8776076_HIV1-Con_M__1_35	4046	4166	TATGCATTAGGAATCATTCAAGCACAACCAGATAAGAGTGAATCAGAGTTAGTCAATCAAATAATAGAGCAGTTAATAAAAAAGGAAAAGGTCTACCTGTCATGGGTACCAGCACACAAA
176861_8776076_HIV1-Con_M__1_36	4165	4285	AGGAATTGGAGGAAATGAACAAGTAGATAAATTAGTCAGTACTGGAATCAGGAAAGTACTATTTTTAGATGGAATAGATAAGGCTCAAGAAGAACATGAAAAATATCACAGCAATTGGAG
176861_8776076_HIV1-Con_M__1_37	4284	4404	GAGCAATGGCTAGTGATTTTAATCTGCCACCTGTAGTAGCAAAAGAAATAGTAGCTAGCTGTGATAAATGTCAGCTAAAAGGGGAAGCCATGCATGGACAAGTAGACTGTAGTCCAGGAA
176861_8776076_HIV1-Con_M__1_38	4403	4523	ATATGGCAATTAGATTGTACACATTTAGAAGGAAAAATTATCCTGGTAGCAGTCCATGTAGCCAGTGGCTATATAGAAGCAGAAGTTATCCCAGCAGAAACAGGACAGGAAACAGCATAC
176861_8776076_HIV1-Con_M__1_39	4522	4642	CTTTATATTAAAATTAGCAGGAAGATGGCCAGTAAAAGTAATACATACAGACAATGGCAGCAATTTCACCAGTGCTGCAGTTAAGGCAGCCTGTTGGTGGGCAGGTATCAAACAGGAATT
176861_8776076_HIV1-Con_M__1_40	4641	4761	TTGGAATTCCCTACAATCCCCAAAGTCAAGGAGTAGTAGAATCTATGAATAAAGAATTAAAGAAAATCATAGGACAGGTAAGAGATCAAGCTGAACATCTTAAGACAGCAGTACAAATGG
176861_8776076_HIV1-Con_M__1_41	4760	4880	GCAGTATTCATTCACAATTTTAAAAGAAAAGGGGGGATTGGGGGGTACAGTGCAGGGGAAAGAATAATAGACATAATAGCAACAGACATACAAACTAAAGAATTACAAAAACAAATTACA
176861_8776076_HIV1-Con_M__1_42	4879	4999	AAAAATTCAAAATTTTCGGGTTTATTACAGGGACAGCAGAGACCCAATTTGGAAAGGACCAGCAAAACTACTCTGGAAAGGTGAAGGGGCAGTAGTAATACAAGACAATAGTGAAATAAA
176861_8776076_HIV1-Con_M__1_43	4998	5118	AGGTAGTACCAAGAAGAAAAGCAAAGATCATTAGGGATTATGGAAAACAGATGGCAGGTGATGATTGTGTGGCAGGTAGACAGGATGAGGATTAGAACATGGAAAAGTTTAGTAAAACAC
176861_8776076_HIV1-Con_M__1_44	5117	5237	CCATATGTATGTTTCAAAGAAAGCTAAAGGATGGTTTTATAGACATCACTATGAAAGCAGGCATCCAAAAATAAGTTCAGAAGTACACATCCCACTAGGGGATGCTAGATTAGTAGTAAC
176861_8776076_HIV1-Con_M__1_45	5236	5356	CAACATATTGGGGTCTGCATACAGGAGAAAGAGACTGGCATTTGGGTCAGGGAGTCTCCATAGAATGGAGGCAGAGAAGATATAGCACACAAGTAGACCCTGGCCTGGCAGACCAACTAA
176861_8776076_HIV1-Con_M__1_46	5355	5475	ATTCATATGTATTATTTTGATTGTTTTTCAGAATCTGCCATAAGGAAAGCCATATTAGGACATATAGTTAGTCCTAGGTGTGAATATCAAGCAGGACATAACAAGGTAGGATCTCTACAA
176861_8776076_HIV1-Con_M__1_47	5474	5594	ATATTTGGCACTAACAGCATTAATAACACCAAAAAAGATAAAGCCACCTTTGCCTAGTGTTAGGAAACTAGCAGAGGATAGATGGAACAAGCCCCAGAAGACCAGGGGCCACAGAGGGAG
176861_8776076_HIV1-Con_M__1_48	5593	5713	GCCATACAATGAATGGACACTAGAGCTTTTAGAGGAGCTTAAGAATGAAGCTGTTAGACATTTTCCTAGGCCATGGCTCCATGGCTTAGGACAACATATCTATAACACCTATGGGGATAC
176861_8776076_HIV1-Con_M__1_49	5712	5832	CTTGGGAAGGAGTTGAAGCTATAATAAGAATACTGCAACAACTACTGTTTATTCATTTCAGAATTGGGTGCCAACATAGCAGAATAGGCATTATTCGACAGAGAAGAGTAAGAAATGGAG

176861_8776076_HIV1-Con_M__1_50	5831	5951	GCCAGTAGATCCTAACCTAGAGCCCTGGAACCATCCAGGAAGTCAGCCTAAAACTGCTTGTAACAAATGTTATTGTAAAAAGTGTTGCTATCATTGCCAAGTTTGCTTTATAAAGAAAGG
176861_8776076_HIV1-Con_M__1_51	5950	6070	GCTTAGGCATCTCCTATGGCAGGAAGAAGCGGAGACAGCGACGAAGAACTCCTCAAAGCAGTCAGGATCATCAAAATCCTATACCAAAGCAGTAAGTAGTATATGTAATGCAACCTTTAG
176861_8776076_HIV1-Con_M__1_52	6069	6189	GAAATATTAGCAATAGTAGCATTAATAGTAGCACTAATAATAGCAATAGTTGTGTGGACCATAGTATGTATAGAATATAGGAAAATGATAAGACAAAGAAAAATAGACAGGTTAATTGAA
176861_8776076_HIV1-Con_M__1_53	6188	6308	AAGAATAAGAGAAAGAGCAGAAGACAGTGGCAATGAGAGTGAGGGGGATACAGAGGAATTGTCAACACTTGTGGAAATGGGGCATCTTGCTCCTTGGGATGTTAATGATCTGTAGTGCTG
176861_8776076_HIV1-Con_M__1_54	6307	6427	GCAGAAAACTTGTGGGTCACAGTCTATTATGGGGTACCTGTGTGGAAAGAAGCAAACACCACTCTATTTTGTGCATCAGATGCTAAAGCATATGAAACAGAAGTACATAATGTCTGGGCT
176861_8776076_HIV1-Con_M__1_55	6426	6546	TACACATGCCTGTGTACCCACAGACCCCAACCCACAAGAAATAGTTCTGGAAAATGTAACAGAAAATTTTAACATGTGGAAAAATAACATGGTAGAACAGATGCATGAAGATATAATCAG
176861_8776076_HIV1-Con_M__1_56	6545	6665	GTTTATGGGATCAAAGCCTAAAGCCATGTGTAAAGTTAACCCCACTCTGTGTTACTTTAAACTGTACTGATGTTAATAATACTAATAATACTAATAATAATAATGGGAGAATGATGGAAG
176861_8776076_HIV1-Con_M__1_57	6664	6784	GAGAAAGAAGAAATAAAAAACTGCTCTTTCAATATAACCACAGAAATAAGAGATAAGAAGAAGAAAGAATATGCACTTTTTTATAGACTTGATATAGTACCAATTAATAATAATAATAAT
176861_8776076_HIV1-Con_M__1_58	6783	6903	TAATAGTAATTATAGGTTAATAAATTGTAATACCTCAGCCATTACACAGGCTTGTCCAAAGGTATCCTTTGAGCCAATTCCCATACATTATTGTGCTCCAGCTGGTTTTGCGATTCTAAA
176861_8776076_HIV1-Con_M__1_59	6902	7022	AGTGTAATGATAAGAAGTTCAATGGAACAGGACCATGCAAAAATGTCAGCACAGTACAATGTACACATGGAATTAAGCCAGTGGTATCAACTCAACTGCTGTTAAATGGCAGTCTAGCAG
176861_8776076_HIV1-Con_M__1_60	7021	7141	GAAGAAGAGATAATAATTAGATCTGAAAATATCACAGACAATGCCAAAACCATAATAGTACAGCTTAATGAATCTGTAGAAATTAATTGTACAAGACCCAACAACAATACAAGAAAAAGT
176861_8776076_HIV1-Con_M__1_61	7140	7260	TATACGTATAGGACCAGGACAAGCATTCTATGCAACAGGTGAAATAATAGGAGATATAAGACAAGCACATTGTAACATTAGTGGAACAAAATGGAATAAAACTTTACAACAGGTAGCTAA
176861_8776076_HIV1-Con_M__1_62	7259	7379	AAAAATTAAGAGAACACTTTAATAATAAAACAATAATCTTTAAACCATCCTCAGGAGGGGACCTAGAAATTACAACACATAGTTTTAATTGTGGAGGAGAATTTTTCTACTGCAATACAT
176861_8776076_HIV1-Con_M__1_63	7378	7498	TCAGGACTGTTTAATAGTACATGGAATAATAATAGTAGTACTGAAGGGTCAAATAACAGTAAAACAAATGACACTATCACACTCCCATGCAGAATAAAACAAATTATAAACATGTGGCAG
176861_8776076_HIV1-Con_M__1_64	7497	7617	GAGAGTAGGACAAGCAATGTATGCCCCTCCCATTGCAGGAAAAATTACATGTAAATCAAATATTACAGGACTACTATTGACAAGAGATGGTGGTAATAATAATAATAAGAATGAGACCTT
176861_8776076_HIV1-Con_M__1_65	7616	7736	TCAGACCTGGAGGAGGAGATATGAGGGACAATTGGAGAAGTGAATTATATAAATATAAAGTAGTAAAAATTGAACCACTAGGAGTAGCACCCACCAAGGCAAAGAGAAGAGTGGTGGAGA
176861_8776076_HIV1-Con_M__1_66	7735	7855	AGAGAAAAAAGAGCAGTGGGAATAGGAGCTGTGTTCCTTGGGTTCTTGGGAGCAGCAGGAAGCACTATGGGCGCAGCGTCAATAACGCTGACGGTACAGGCCAGACAATTATTGTCTGGT
176861_8776076_HIV1-Con_M__1_67	7854	7974	TATAGTGCAACAGCAAAGCAATTTGCTGAGGGCTATAGAGGCGCAACAGCATCTGTTGCAACTCACAGTCTGGGGCATTAAACAGCTCCAGGCAAGAGTCCTGGCTGTGGAAAGATACCT
176861_8776076_HIV1-Con_M__1_68	7973	8093	TAAAGGATCAACAGCTCCTAGGGATTTGGGGCTGCTCTGGAAAACTCATCTGCACCACTAATGTGCCCTGGAACTCTAGTTGGAGTAATAAATCTCAGGATGAGATTTGGGATAACATGA
176861_8776076_HIV1-Con_M__1_69	8092	8212	ACCTGGATGCAGTGGGAAAGAGAAATTAACAATTACACAGACATAATATACAGCTTAATTGAAGAATCGCAAAACCAGCAGGAAAAGAATGAACAAGAATTATTGGCATTGGACAAGTGG
176861_8776076_HIV1-Con_M__1_70	8211	8331	GGCAAGTCTGTGGAATTGGTTTGACATAACAAATTGGCTGTGGTATATAAAAATATTCATAATGATAGTAGGAGGCTTGATAGGTTTAAGAATAGTTTTTGCTGTGCTTTCTATAGTAAA
176861_8776076_HIV1-Con_M__1_71	8330	8450	ATAGAGTTAGGCAGGGATACTCACCTTTGTCATTTCAGACCCTTATCCCAAACCCGAGGGGACCCGACAGGCCCGAAGGAATCGAAGAAGAAGGTGGAGAGCAAGACAGAGACAGATCCA
176861_8776076_HIV1-Con_M__1_72	8449	8569	ATTCGATTAGTGAACGGATTCTTAGCACTTGTCTGGGACGACCTGCGGAGCCTGTGCCTCTTCAGCTACCACCGCTTGAGAGACTTCATCTTGATTGCAGCGAGGACTGTGGAACTTCTG
176861_8776076_HIV1-Con_M__1_73	8568	8688	GGGACGCAGCAGTCTCAAGGGACTGCGAAGGGGGTGGGAAGCCCTCAAATATCTGTGGAATCTCCTGCAGTATTGGGGTCAGGAACTAAAGAATAGTGCTATTAGTTTGCTTGATACCAC
176861_8776076_HIV1-Con_M__1_74	8687	8807	CAGCAATAGCAGTAGCTGAGGGGACAGATAGGGTTATAGAAGTAGTACAAAGAGCTTGTAGAGCTATTCTCAACATACCTAGAAGAATAAGACAGGGCTTTGAAAGGGCTTTGCTATAAA
176861_8776076_HIV1-Con_M__1_75	8806	8926	AATGGGTGGCAAGTGGTCAAAAAGTAGTATAGTTGGATGGCCTGCTGTAAGGGAAAGAATGAGACGAACTGCTCCAGCAGCAGAAGGGGTAGGAGCAGTATCTCAAGACTTAGATAAACA
176861_8776076_HIV1-Con_M__1_76	8925	9045	ATGGGGCAATCACAAGCAGCAATACAGCAGCTACTAATGCTGACTGTGCCTGGCTGGAAGCACAAGAGGAGGAGGAAGTAGGCTTTCCAGTCAGACCTCAGGTACCTTTAAGACCAATGA
176861_8776076_HIV1-Con_M__1_77	9044	9164	ACTTATAAGGCAGCTTTTGATCTCAGCCACTTTTTAAAAGAAAAGGGGGGACTGGAAGGGTTAATTTACTCCAAGAAAAGACAAGAGATCCTTGATCTGTGGGTCTACCACACACAAGGC
176861_8776076_HIV1-Con_M__1_78	9163	9283	CTACTTCCCTGATTGGCAGAACTACACACCAGGGCCAGGGATCAGATACCCACTGACCTTTGGATGGTGCTTCAAGCTAGTACCAGTTGATCCAGAGGAGGTAGAAGAGGCCAATGAAGG
176861_8776076_HIV1-Con_M__1_79	9282	9402	GAGAGAACAACTGCTTGTTACACCCTATGAGCCAGCATGGAATGGAGGATGAAGACAGAGAAGTGTTAATGTGGAAGTTTGACAGCCGCCTAGCACTGAGACACATAGCCCGAGAGCTGC
176861_8776076_HIV1-Con_M__1_80	9401	9521	CATCCGGAGTTCTACAAAGACTGCTGACACAGAAGTTGCTGACAGGGACTTTCCGCTGGGGACTTTCCAGGGGAGGCGTGGCCTGGGCGGGACTGGGGAGTGGCTAACCCTCAGATGCTG
176861_8776076_HIV1-Con_M__1_81	9520	9640	GCATATAAGCAGCTGCTTTTCGCCTGTACTGGGTCTCTCTGGTTAGACCAGATCTGAGCCTGGGAGCTCTCTGGCTAGCTAGGGAACCCACTGCTTAAGCCTCAATAAAGCTTGCCTTGA
176861_8776076_HIV1-Con_M__1_82	9639	9731	AGTGCTTTAAGTAGTGTGTGCCCGTCTGTTGTGTGACTCTGGTAACTAGAGATCCCTCAGACCACTTTAGTCAGTGTGGAAAATCTCTAGCA
176861_8776077_Con_M_7056-7462-B_1_1	0	120	AAATTGCACTGATGTGAAGAATGCTACTAATACCAATAGTACTAATACCAATAATAGTAGCAGGGAAATGATTGAGGAAGAAGGAGAAATAAAAAACTGCTCTTTCAATATTACCACAAG
176861_8776077_Con_M_7056-7462-B_1_2	119	239	GAATAAGAAATAAGGTGCAGAAAGAATATGCACTTTTTTATAAACTTGATGTAGTACCAATAGATAATGATAGAACTAATAATACCAGCTATAGGTTGGTAAGTTGTAATACCTCAGTCA
176861_8776078_Con_M_2467-2616-B_1_1	1	120	CAGAAGAGAGCTTCAGGTTTGGGGAAGAGACAACAACTCCCTCTCAGAAGCAGGAGCCGATAGACAAGGAACTGTATCCTTTAGCTTCCCTCAGATCACTCTTTGGCAACGACCCCTCG
176861_8776079_Con_M_6368-6796-B_1_1	0	120	AGCGACGAAGAGCTCCTCAAGACAGTCAGACTCATCAAGTTTCTCTATCAAAGCAGTAAGTAGTACATGTAATGCAATCTTTACAAATATTAGCAATAGTAGCATTAGTAGTAGCAGTAA
176861_8776079_Con_M_6368-6796-B_1_2	119	239	ATAATAGCAATAGTTGTGTGGTCCATAGTATTCATAGAATATAGGAAAATATTAAGACAAAGAAAAATAGACAGGTTAATTGATAGAATAAGAGAAAGAGCAGAAGACAGTGGCAATGAG
176861_8776079_Con_M_6368-6796-B_1_3	238	358	GAGTGAAGGGGATCAGGAAGAATTATCAGCACTTGTGGAGATGGGGCACCATGCTCCTTGGGATGTTGATGATCTGTAGTGCTGCAGAAAACTTGTGGGTCACAGTCTATTATGGGGTAC
176861_8776080_Con_M_9296-9761-B_1_1	0	120	GAGACTTACTCTTGATTGTAACGAGGATTGTGGAACTTCTGGGACGCAGGGGGTGGGAAGCCCTCAAATATTGGTGGAATCTCCTACAGTATTGGAGTCAGGAACTAAAGAATAGTGCTG
176861_8776080_Con_M_9296-9761-B_1_2	120	240	TTAGCTTGCTTAATGCCACAGCCATAGCAGTAGCTGAGGGGACAGATAGGGTTATAGAAGTAGTACAAAGAGCTTATAGAGCTATTCTCCACATACCTAGAAGAATAAGACAGGGCTTGG
176861_8776080_Con_M_9296-9761-B_1_3	240	359	AAAGGGCTTTGCTATAAGATGGGTGGCAAGTGGTCAAAACGTAGTGTGGGTGGATGGCCTGCTGTAAGGGAAAGAATGAGACGAGCTGAGCCAGCAGCAGATGGGGTGGGAGCAGTATC
176861_8776081_Con_M_10162-10299-B_1_1	1	120	ACAACTGCTTGTTACACCCTATGAGCCAGCATGGAATGGATGACCCGGAGAAAGAAGTGTTAGTGTGGAAGTTTGACAGCCGCCTAGCATTTCATCACATGGCCCGAGAGCTGCATCCG
176861_8776082_Con_M_9697-9858-BD_1_1	1	120	CAGCAGCAGATGGGGTGGGAGCAGTATCTCGAGACCTGGAAAAACATGGGGCAATCACAAGTAGCAATACAGCAGCTACTAATGCTGACTGTGCCTGGCTAGAAGCACAAGAGGAGGAG
176861_8776083_Con_M_7056-7480-D_1_1	0	120	AAACTGTAGTGATGAATTGAGGAACAATGGCACTATGGGGAACAATGTCACTACAGAGGAGAAAGGAATGAAAAACTGCTCTTTCAATGTAACCACAGTACTAAAAGATAAGAAGCAGCA
176861_8776083_Con_M_7056-7480-D_1_2	119	239	AAGTATATGCACTTTTTTATAGACTTGATATAGTACCAATAGACAATGATAGTAGTACCAATAGTACCAATTATAGGTTAATAAATTGTAATACCTCAGCCATTACACAGGCTTGTCCAA
176861_8776084_Con_M_7722-8005-Dcm_1_1	0	120	GCAGCTTAACAATACTATAAACATTACTTGTATAAGGCCTGGCAACAATACAAGAAAAAGTATACATTTAGGACCAGGGCAGGCATTCTATGCAAAAAATATAATAGGAGACATAAGAAA
176861_8776084_Con_M_7722-8005-Dcm_1_2	119	239	AAGCACATTGTAATATTAGTGGAACAAAATGGAATAACACTCTACAACAGGTAGCTAAAAAATTAGGAAAGCTCTATAACAAAACAATAATTTTTAAACCATCCTCAGGAGGGGACCCAG
176861_8776085_Con_M_180-486-D_1_1	0	120	CCACTGAAGGAGAGAACAACTGCTTGTTACACCCTATGTGCCAGCATGGAATGGAGGACCCGGAGAGAGAAGTGTTAGTGTGGAGATTTAACAGCAGACTAGCATTTGAACACAAGGCCC
176861_8776085_Con_M_180-486-D_1_2	119	239	CGAGAGCAGCATCCGGAGTTCTACAAAGACTGCTGACACCGAGGGAAAGACTGCTGACACCGAGTTTTCTACAAGGGACTTTCCGCTGGGGACTTTCCAGGGAGGCGTGGCCTGGGCGGG
176861_8776086_Con_M_2135-2263-D_1_1	1	120	GAGCCAAGCAACAAGTGCAGCTGCTGCAATAATGATGCAGAGAGGCAATTTTAAGGGCCCAAGGAAAATTGTTAAGTGTTTCAACTGTGGCAAAGAAGGGCACATAGCAAAAAATTGCA
176861_8776087_Con_M_2138-2263-D_1_1	1	120	CCAAGCAACAAGTGCAAATGCTGCTGCAATAATGATGCAGAGAGGCAATTTTAAGGGCCCAAGGAAAATTGTTAAGTGTTTCAACTGTGGCAAAGAAGGGCACATAGCAAAAAATTGCA

176861_8776088_Con_M_180-506-A_1_1	0	120	CCACTGAGGGAGAGAACAACAGCCTATTACACCCTATATGCCAACATGGAATGGATGATGAGGAGAAAGAAGTGTTAATGTGGAAGTTTGACAGCCGCCTGGCACTAAAACACAGAGCCC
176861_8776088_Con_M_180-506-A_1_2	119	239	CGAGAGCTGCATCCGGAGTTCTACAAAGACTGCTGACACAGAAGTTGCTGACAAGGGACTTTCCGCTGGGGACTTTCCAGGGGAGGTGTGGTTTGGGCGGAGTTGGGGAGTGGCTAACCC
176861_8776089_Con_M_2545-2671-D_1_1	1	120	AGAAGGACAAAGACAAGGAACTGTATCCTTTAGCTTCCCTCAAATCACTCTTTGGCAACGACCCCTTGTCACAATAAAGATAGGGGGACAGCTAAAGGAAGCTCTATTAGATACAGGAG
176861_8776090_Con_M_6367-6649-D_1_1	0	120	CAGCGACGAAGACCTCCTCAAGGCGGTCAGACTCATCAAGTTCCTGTACCAAAGCAGTAAGTAGTACATGTAATGCAACCTTTAGAGATATTAGCAATAGTAGCATTAGTAGTAGCGCTA
176861_8776090_Con_M_6367-6649-D_1_2	119	239	AATAATAGCAATAGTTGTGTGGACCATAGTATTCATAGAATATAGGAAAATAAAAAGGCAAAGAAAAATAGACTGGTTAATTGATAGAATAAGAGAAAGAGCAGAAGACAGTGGCAATGA
176861_8776091_Con_M_7722-8002-D_1_1	0	120	ACAGCTTAATGAATCTGTAGAAATTAATTGTACAAGGCCCTACAACAATACAAGACAAAGTACACCTATAGGACCAGGGCAAGCACTCTATACAACAAAGATAATAGGAGATATAAGACA
176861_8776091_Con_M_7722-8002-D_1_2	119	239	AAGCACATTGTAATATTAGTAGAACAGAATGGAATAAAACTTTACAACAGGTAGCTAAAAAATTAGGAAACCTTTTTAACAAGACAACAATAATTTTTAAACCATCCTCAGGAGGGGACC
176861_8776092_Con_M_9296-9436-D_1_1	1	120	AGACTTAATCTTAATTGCAGCGAGGATTGTGGAACTTCTGGGACGCAGGGGGTGGGAAGCCCTCAAATATCTGTGGAATCTCCTGCAGTATTGGAGTCAGGAACTGAAGAATAGTGCTA
176861_8776093_Con_M_10162-10299-D_1_1	1	120	ACAACTGCTTGTTACACCCTATATGCCAGCATGGAATGGAGGACCCGGAGAGAGAAGTGTTAGTGTGGAGATTTAACAGCAGACTAGCATTTGAACACAAGGCCCGAGAGAAGCATCCG
176861_8776094_Con_M_7056-7507-A1_1_1	0	120	ACATTGTACAAATGTCACAAGTGTCAACACCACTGGAGACAGGGAAGGCTTAAAAAACTGCTCTTTCAATATGACCACAGAACTAAGGGATAAGAGACAGAAAGTATATTCACTTTTTTA
176861_8776094_Con_M_7056-7507-A1_1_2	119	239	ATAGACTTGATATAGTACCAATTAATGAAAATCAGGGTAGTGAATATAGATTAATAAATTGTAATACCTCAGCCATTACACAGGCGTGCCCAAAGGTATCCTTTGAGCCAATTCCTATAC
176861_8776095_Con_M_9726-9875-A_1_1	1	120	GGAGTAGGAGCAGTATCTCAAGATTTAGATAAACATGGAGCAGTCACAAGCAGGAATATAAATCACCCTAGTTGCGTCTGGCTGGAAGCGCAAGAGGAAGAGGAGGTAGGCTTTCCAGT
176861_8776096_Con_M_2122-2259-A_1_1	1	120	TGGCTGAGGCAATGAGTCAAGTACAACATACAAACATAATGATGCAGAGAGGCAATTTTAGGGGCCAGAAAAGAATTAAGTGTTTCAACTGTGGCAAAGAAGGACACCTAGCCAGAAAT
176861_8776097_Con_M_2475-2621-A_1_1	1	120	GAGAGCTTTGGGATGGGGGAAGAGATAACCTCCCCTCTGAAGCAGGAGCAGAAAGACAGGGAACAGCACCCTCCTTCAGTTTCCCTCAAATCACTCTTTGGCAACGACCCCTTGTCACA
176861_8776098_Con_M_2545-2674-A_1_1	1	120	AGAAAGACAGGGAACAGCACCCTCCTTCAGGTTCCCTCAAATCACTCTTTGGCAACGACCCCTTGTCACAGTAAAAATAGGAGGACAGCTAAAAGAAGCTCTATTAGATACAGGAGCAG
176861_8776099_Con_M_6362-6629-A_1_1	0	120	GGAGACACCGACGAGGAACTCCTCAGAGCAGTAAGGATCATCAAAATCCTATACCAAAGCAGTAAGTATTAGTAATTAATATATGTAATGACTCCTTTGGAAATCTGTGCAATAGTAGGA
176861_8776099_Con_M_6362-6629-A_1_2	119	239	ACTGATAGTAGCGTTAATCTTAGCAATAGTTGTGTGGACTATAGTAGGTATAGAATATAAGAAATTGCTAAAGCAAAGGAAAATAGACAGGTTAATTGAAAGAATAAGAGAAAGAGCAGA
176861_8776100_Con_M_9726-9869-A_1_1	1	120	GGAGTAGGAGCAGTATCTCAAGATTTAGATAAACATGGAGCAGTCACAAGCAGTAATACAGCAGCTACTAATGCTGATTGCGCCTGGCTGGAAGCGCAAGAGGAAGAGGAGGTAGGCTT
176861_8776101_Con_M_10126-10263-A_1_1	1	120	CAGATGAAGTAGAAGAGGCTACTGAGGGAGAGAACAACAGCCTATTACACCCTATATGCCAACATGGAATGGATGATGATGAGAGAGAAGTGTTAATGTGGAAGTTTGACAGTCGCCTG
176861_8776102_Con_M_7056-7483-02_1_1	0	120	AGAATGTGGTAACGTCACCAAAAACAATTTCACTGACAAAAACATGAAAGAGGAAATAAAAAACTGCTCTTACAATGCAACCACAGAATTAAGAGATAAGACACAGAAAATATATTCACT
176861_8776102_Con_M_7056-7483-02_1_2	119	239	TTTTTTATAGACAGGATGTGGTACAAATTGATGAAAACAATAGTCATGGTAGAAACAGTAGTAATAGGTATAGACTGATAAATTGTAATACCTCAACCATTACACAGGCTTGTCCAAAGG
176861_8776103_Con_M_305-489-crf02_1_1	1	120	TGCATCCGGAGTTCTACAAAGACTGCTGACACAGAAGTTGCTGACAATGCTGACATGAAGTTGCTGACAGGGACTTTCCGCTGGGGACTTTCCGGGGGAGGTGTGGTTTGGGAGGAGTT
176861_8776104_Con_M_716-875-crf02_1_1	1	120	ACTCTAGACGGTGTAAAAAATCTCTAGCAGTGGCGCCCGAACAGGGACCGGAAATTAAAAGGGACTCGAAAGCGAAAGTTCCAGAGAAGTTCTCTCGACGCAGGGACTCGGCTTGCTGA
176861_8776105_Con_M_7056-7480-G_1_1	0	120	AAACTGTACTAATGTCACCACCACCACTAATGGTACTATAGATAATAGCAATAATATCACGGTGGATGGTAAAGAAGAAATAACCAATTGTTCCTTCAATATAACCACAGAAATAAGAGA
176861_8776105_Con_M_7056-7480-G_1_2	119	239	ACAAAATAAGGCAGGAATACGCGCTTTTCTATAAACTTGATGTAGTACCAATTAATAATGATAATAGAAGTTATATGCTAATAAATTGTAATGTCTCATCCATTAAACAGGCTTGTCCAA
176861_8776106_Con_M_297-505-G_1_1	1	120	CCGAGAGCTGCATCCGGAGTACTACAAAGACTGCTGACACAGAAGTTGCTGACAAGGGACTTTCCGCCTGGGACTTTCCGGGGAGGCGCGGCCTGGGAGGGGCTGGGGAGTGGCTAACC
176861_8776107_Con_M_716-870-G_1_1	1	120	ACTCTAGACAGTGTAAAAATCTCTAGCAGTGGCGCCCGAACAGGGACTTGAAAGCGAAAGTTAATAGGGACTCGAAAGCGAAAGTTCCAGAGAAGTTCTCTCGACGCAGGACTCGGCTT
176861_8776108_Con_M_1141-1537-G_1_1	0	120	GAAACAGCAGAAGGTTGTCAACAAATAATGGGACAGTTGCAACCAGCTCTCCAGACAGGAACAGAGGAGATTAGATCATTATTTAATACAGTAGCAACCCTCTATTGTGTACATCAAAAG
176861_8776108_Con_M_1141-1537-G_1_2	119	239	GATAGAGGTAAAAGACACCAAAGAAGCTCTAGAGGAAGTGGAAAAGATACAAAAGAAAAGTCAGCAAAAAATACAGCAGGCAGCAAAGGATGAAGGAAACAGCAGCCAAGTCAGCCAAAA
176861_8776108_Con_M_1141-1537-G_1_3	238	358	ATTATCCTATAGTGCAGAATGCACAAGGGCAAATGGTACACCAGGCCATATCACCTAGAACTTTGAATGCATGGGTAAAAGTAGTAGAAGAAAAGGCCTTCAGCCCAGAAGTAATACCCA
176861_8776109_Con_M_2122-2250-G_1_1	1	120	TAGCTGAGGCAATGAGCCAGGCATCAGGTGCAGCAGCAGCCATAATGATGCAGAAAAGCAATTTTAAGGGCCCAAGAAGAAATATTAAGTGTTTCAACTGTGGCAAGGAAGGACATCTA
176861_8776110_Con_M_6363-6630-G_1_1	0	120	GAAACACCGACGAGGAACTCCTCAGAGCAGTAAGGATCATCAAAATCCTGTACCAAAGCAGTAAGTAGTAATAATTAGTATATGTAATGCAATCATTAGAAATATCTGCAATAGTAGGAC
176861_8776110_Con_M_6363-6630-G_1_2	119	239	CTAATAGTAGCATTCATAGCAGCCATAGTTGTGTGGACCATAGTATTTATAGAATATAGGAAAATAAGAAAACAGAAAAAAATAGAAAAGTTACTTGATAGAATAAGAGAAAGAGCAGAA
176861_8776111_Con_M_9713-9862-G_1_1	1	120	AGAAGGAGTAGGAGCAGTATCTCAAGATTTAGATAAGCATGGAGCAATCACAAGCAGCAATACAGCAGCCAATAATCCTGATTGTGCCTGGCTGGAAGCACAAGAGGAGGATTCAGAGG
176861_8776112_Con_M_10126-10263-G_1_1	1	120	CAGCAGAGGTAGAGGAAGCCAATAAAGGAGAGAACAACAGTTTATTACACCCCATCTGCCAGCATGGAATGGAGGACGAAGACAGAGAAGTGCTGGTATGGAGATTTGACAGGAGCCTA
176861_8776113_Con_M_7056-7435-C_1_1	0	120	AGAATGTGGAAATGTTAGTGTTAGCAATGTTAGTGTTAGCAATACTACCCAGGGGGATGCAAATGTTACCCACAATGAGGGTGCAAATGAAATAAAAAATTGCTCTTTCAAGATAGCCAC
176861_8776113_Con_M_7056-7435-C_1_2	119	239	CAGAATTAAGAGATAAGGAGCAGAAAGTGTATGCGCTTTTTTATAGACTTGATATAGTACAACTTAATGATAGTAGTAACTCTAGTAGTAGTAATAACTCTAGTAAGTATAGTAAGTATA
176861_8776114_Con_M_187-478-C_1_1	0	120	AGGAGAGAACAACTGTTTGCTACACCCTATGAGCCAGCATGGAATGGAGGATGAACACAGAGAAGTATTAAAGTGGAAGTTTGACAGTATCCTAGCACGCAGACACATGGCCCGCGAGCT
176861_8776114_Con_M_187-478-C_1_2	119	239	TACATCCGGAGTATTACAAAGACTGCTGACACAGAAGGGACTTTCCGCTGACACAGAAGGGACTTTCCGCTGGGACTTTCCACTGGGGCGTTCCAGTGGGGCGTTCTAGGAGGAGTGGTC
176861_8776115_Con_M_2390-2554-C_1_1	1	120	GAATTTCCTTCAGAGCAGACCAGAGCCAACAGCCCCACCGCCAGAGCCAACAGCCCCACCAGCAGAGAGCTTCAGGTTCGAGGAGACAACCCCCGCTCCGAAGCAGGAGCCGAAAGACA
176861_8776116_Con_M_6307-6562-C_1_1	0	120	TGTCTAGTTTGCTTTCAGACAAAAGGCTTAGGCATTTCCTATGGCAGGAAGAAGCGGAGACAGCGACGAAGCGCTCCTCCAAGCAGTGAGGATCATCAAAATCCTATATCAAAGCAGTAA
176861_8776116_Con_M_6307-6562-C_1_2	119	239	AGTACTAAATAGTATATGTAATGTTAGATTTAATAGCAAGAGTAGATTATAGATTAGGAGTAGGAGCATTGATAGTAGCACTAATCATAGCAATAGTTGTGTGGACCATAGTATATATAG
176861_8776117_Con_M_7722-7999-C_1_1	0	120	ACATCTTAATGAATCTGTAGAAATTGTGTGTACAAGACCCAACAATAATACAAGAAAAAGTATAAGGATAGGACCAGGACAAGCATTCTATGCAACAGGAGACATAATAGGAGACATAAG
176861_8776117_Con_M_7722-7999-C_1_2	119	239	GACAAGCACATTGTAACATTAGTAAAAATAAATGGAATAAAACTTTACAAAAGGTAAGGGAAAAATTAGAAAAACACTTCCCTAATAAAACAATAAAATTTGAACCACCCTCAGGAGGGG
176861_8776118_Con_M_9712-9864-C_1_1	1	120	CAGAGGGAGTAGGAGCAGCGTCTCAAGACTTAGATAAACATGGGGCACTTACAAGCAGCAACACACCCGCCAATAATGCTGATTGTGCCTGGCTGGAAGCGCAAGAGGAGGAAGAAGAA
176861_8776119_Con_M_10169-10309-C_1_1	1	120	TTTGCTACACCCTATGAGCCAGCATGGAATGGAGGATGAACACAGAGAAGTATTAAAGTGGAAGTTTGACAGTCACCTAGCACGCAGACACATGGCCCGCGAGCTACATCCGGAGTATT
176861_8776120_Con_M_7056-7476-F1_1_1	0	120	AAATTGTACTAATGCCACTACCACCAATGACACTCTCTCTGACCAAAGCAGTACCCTGAAGGAAGAACCAGGGGCAATACAAAACTGTTCTTTCAATATGACCACAGAAGTAGAAGATAA
176861_8776120_Con_M_7056-7476-F1_1_2	119	239	AGAAGCAGAAAGTACATGCGCTTTTTTATAGACTTGATATAGAACCAATCAGCAATAATAATAGCAGGGAAGAATATAGGCTAATAACTTGTAATACCTCAACCATTACACAGGCTTGTC
176861_8776121_Con_M_297-450-F_1_1	1	120	CAGAGAGAAACATCCGGAGTTCTACCAAGACTGAGACTGCTGACACAGAGATTGCTGACATAGAAGATTAGAGATTGCTGACATAGAAGATTCTAAAGGGACTTTCCGCTGGGGACTTT
176861_8776122_Con_M_1104-1363-F_1_1	0	120	GGAGCTAGAACGATTTGCACTTGATCCTGGCCTTCTAGAAACATCAGAAGGCTGTCAGAAAATAATAGGACAGCTACAACCATCCCTTCAGACAGGATCAGAAGAGCTTAGATCATTATA
176861_8776122_Con_M_1104-1363-F_1_2	119	239	ATAATACAGTAGCAGTCCTCTATTATGTACATCAAAAGATAGAGGTAAAGGACACCAAGGAAGCTTTAGATAAGCTAGAGGAAGAACAAAACAAAAGTCAGCAAAAGACACAGCAAGCGG
176861_8776123_Con_M_2545-2674-F_1_1	1	120	AGAAAGACGAGGGACAGTATCCTCCCTTAGCTTCCCTCAAATCACTCTTTGGCAACGACCCTTAGTCACAATAAAAGTAGGGGGGCAGCTAAGGGAAGCTCTATTAGATACAGGGGCAG

176861_8776124_Con_M_5536-5658-F2_1_1	1	120	ATCCAAGAGTAAGTTCAGAAGTACACATCCCACTAGAGGATGATTCTAAATTAGTAATAGTAACCTATTGGGGTCTACATACAGGAGAAAGAGATTGGCATTTGGGTCAAGGAGTCTCC
176861_8776125_Con_M_6299-6693-F_1_1	0	120	GCTTTCATTGCTACTGGTGCTTTACAACGAAGGGCTTAGGCATCTCCTATGGCAGGAAGAAGCGGAGACAGCGACGAAGAACTCCTCAAAGCAGTCAGATACATCAAGATCCTGTACCAA
176861_8776125_Con_M_6299-6693-F_1_2	119	239	AAGCAGTAAGTATTGTTAAGTAGTATATGTAATGTCATATTTGTTAGCAATAGCAATAGTAGCATTAATAGTAGCACTAATAATAGCAATAGTTGTGTGGACTATAGTATATATAGAATA
176861_8776125_Con_M_6299-6693-F_1_3	238	358	ATAAGAAACTGTTAAGGCAAAGAAAAATAAATAGGTTATATGAAAGAATAAGAGAAAGAGCAGAAGACAGTGGCAATGAGAGTGAGGGGGATGCAGAGGAATTGGCAGCACTTGGGGAAA
176861_8776126_Con_M_9296-9558-F_1_1	0	120	GAGACTTCATATTAATTGCAGCGAGGACTGTGGACAGGGGACTGAGGAGGGGGTGGGAAGCCCTCAAATATCTGTGGAATCTCACGCAGTATTGGAGTCAGGAACTAAAGAATAGTGCTA
176861_8776126_Con_M_9296-9558-F_1_2	119	239	ATTAGTCTGCTTAATACCACAGCAATAGTAGTAGCTGAGGGGACAGATAGAGTTATAGAAGCTTTGCAAAGAGCTGGTAGAGCTGTTCTCAACATACCTAGAAGAATAAGACAGGGCTTT
176861_8776127_Con_M_9700-9858-F_1_1	1	120	CAGCAGAAGGGGTGGGAGCAGTGTCTCAAGACTTAGATAGACGGGGGGCAATTACAAGCAGCAATACTGGAGCTACTAATCCTGACTTGGCCTGGCTGGAAGCACAAGAGGAAGAGGAA
176861_8776128_Con_M_180-299-02FGHJK_1_[illegible]	1	120	CAATGAAGGAGAGAACAACAGCTTATTACACCCCATGTGCCAACATGGAATGGAGGATGAAGACAGAGAAGTACTGATGTGGAAGTTTGACAGCAGCCTGGCACTGAGACACATAGCCC
176861_8776129_Con_M_892-1032-H_1_1	1	120	AGGCGAGGGGCGGCGCGACTGGTGAGTACGCCGAAAAATTTTTATTTTGACTAGCGGAGGCTAGAAGGAGAGAGATGGGTGCGAGAGCGTCAGTATTAAGCGGGGGAAAATTAGATGCT
176861_8776130_Con_M_6305-6587-H_1_1	0	120	ATTGCCAATTGTGCTTTTTAAAGAAAGGCTTAGGCATTTCCTATGGCAGGAAGAAGCGGAGACAGCGACGAGGAACTCCTGCAAGTTTGCAAGATCATCAAAATCCTATACCAAAGCAGT
176861_8776130_Con_M_6305-6587-H_1_2	119	239	TAAGTATCATAAATGTATATATTAGGAATAGGAATAGGAGCACTAGTAGTAGCATTTATCATAGCCATAATTGTGTGGACCATAGTATATATAGAATATAGGAAACTGGTAAAGCAAAAG
176861_8776131_Con_M_7722-7999-H_1_1	0	120	ACAGCTTAATGAACCTGTAGAAATTAACTGTACCAGACCTAACAATAATACAAGAAAAAGTATACATATAGGGCCAGGACAAGCATTCTATGCAACAGGTGACATCATAGGAGATATAAG
176861_8776131_Con_M_7722-7999-H_1_2	119	239	GACAAGCACATTGTAATATTAGTGGAAAAAAATGGAATAAGACTTTACACAAGGTAGTTACACAATTAAGAAAACACTTTAACAATAAAACAATAATTTTTAAACCATCCTCAGGAGGGG
176861_8776132_Con_M_9300-9440-H_1_1	1	120	TTACTCTTGATTGTAGCGAGGACTGTGGAACTTCTGGGGAGAAGGGGGTGGGAAGCCCTCAAATATCTGTGGAATCTTCTGCAGTATTGGGGACAGGAGCTAAAGAATAGTGCTATTAA
176861_8776133_Con_M_9712-9861-H_1_1	1	120	CAGAAGGGGTAGGAGCAGTGTCTCGAGATTTGGATAGACGTGGGGCAGTCACAATCAATAATACAGCAGCTACTAATCCTGATTCCGCCTGGCTGGAAGCACAAGAGGAGGAAGAGGAA
176861_8776134_Con_M_304-488-HJ_1_1	1	120	ATGCATCCGGAGTTCTACAAAGACTGCTGACACAGAAGATGCTGACAATGCTGACACGGAGGATTCTACAGGGACTTTCCGCTGGGGACTTTCCAGGGGAGGTGTGGTCTGGGCGGGGT
176861_8776135_Con_M_1134-1411-J_1_1	0	120	CCTTCTAGAGTCAGCAAAAGGCTGTCAACAAATACTAGTACAGCTCCAACCAGCTCTTCAGACAGGAACAGAAGAAATTAAATCATTATATAATACAGTAGCAACCCTCTATTGCGTACA
176861_8776135_Con_M_1134-1411-J_1_2	119	239	ATCAGAGGATAGAGATAAAAGACACCAAGGAAGCTTTAGACAAGATAGAGGAACTTCAAAACAAAAACAAGCAGCAGGCACAGAAAGCAGAAACTGACAAAAAAGACAACAGTCAGGTCA
176861_8776136_Con_M_6374-6793-J_1_1	0	120	GATCAGCTCCTCCTGGCAGTAAGAATCATCAAGATCTTATACCAGAGCAGTAAGTAAATCAATTAGCATATGTAATGATACCTTTACAAATAGCAGCAATAGTAGCATTAATAGTAGCAT
176861_8776136_Con_M_6374-6793-J_1_2	119	239	TTTTTCCTTGCAATAGGTGTGTGGACTATAGTGTATATAGAATATAGGAAACTGTTAAGACAAAGGAAAATAGATAAGTTAATTGATAGAATAAGAGAAAGAGCAGAAGACAGTGGTAAC
176861_8776136_Con_M_6374-6793-J_1_3	238	358	CGAGAGTGATGGAGACACAGATGAGTTGGCAAAACTTGTGGAGATGGGGCCTCATGATCTTTGGAATGTTAATGATTTGTAATGCTACAGAAAACTTGTGGGTCACAGTCTATTATGGGG
176861_8776137_Con_M_7722-7999-J_1_1	0	120	ACAACTTAATGAAACTGTAGAAATTATGTGTACCAGACCTAACAACAATACAAGAAAAGGTATACACATGGGACCAGGACAAGTGCTCTATGCAACAGGGGAAATAATAGGAGATATAAG
176861_8776137_Con_M_7722-7999-J_1_2	119	239	GAAAAGCACATTGTAACATTAGTGGAAAAGATTGGAATAACACTTTACGTAGAGTAGCTACAAAACTAAGAGAACACTTTAATAATAAAACAATAAACTTTACATCACCCTCAGGAGGGG
176861_8776138_Con_M_9296-9559-J_1_1	0	120	GAGACTTCGTCTTGATTGCAGCGAGGACTGTGGAAACTCTGGGACTCAGGGGGTGGGAGATCCTCAAATATCTGGGGAACCTTGTATGGTATTGGGGGCAGGAACTAAAGAATAGTGCTA
176861_8776138_Con_M_9296-9559-J_1_2	119	239	ATTAGTTTGCTTAATACCACAGCAATAGCAGTAGCTGAAGGAACAGATAGAATCATAGAAATAGTACAAAGAGCTTTTAGAGCTATTCTTCACATACCTAGAAGAATAAGACAGGGTTTA
176861_8776139_Con_M_10126-10263-J_1_1	1	120	CAAGCGAAGTAGAGGAAGCTAATGAAGGAGAGAACAACTGCCTGTTACACCCCATATGCCAGCATGGAATAGAGGATGAAGAAAGAGAAGTGTTAATGTGGAAGTTTGACAGCTCCCTA
176861_8776140_Con_M_9296-9436-K_1_1	1	120	AGACTTAATCTTAATTGCGACGAGGATTCTGGAAAGGGGACTGAGGGGGGGTTGGGAAGCCCTCAAATATCTGTGGAACCTGGTACAGTATTGGGGTCAGGAACTAAAGAATAGTGCCA
176861_8776141_Con_M_6931-7050all_1_1	1	120	ATGTAACAGAAAATTTTAACATGTGGAAAAATAACATGGTAGAACAGATGCATGAAGATATAATCAGTTTATGGGATCAAAGCCTAAAGCCATGTGTAAAGTTAACCCCACTCTGTGTT
176861_8776142_Con_M_7470-7589-all_1_1	1	120	GCTTGTCCAAAGGTATCCTTTGAGCCAATTCCCATACATTATTGTGCTCCAGCTGGTTTTGCGATTCTAAAGTGTAATGATAAGAAGTTCAATGGAACAGGACCATGCAAAAATGTCAG
176861_8776143_Con_M_8341-8460-all_1_1	1	120	AGACCTTCAGACCTGGAGGAGGAGATATGAGGGACAATTGGAGAAGTGAATTATATAAATATAAAGTAGTAAAAATTGAACCACTAGGAGTAGCACCCACCAAGGCAAAGAGAAGAGTG
176861_8776144_HIV1-ConN_1_1	0	120	TTCTCGCTTGTACTGGGTCTCTCTTGCTGGACCAGATTAGAGCCTGGGAGCTCTCTGGCTAGCAGGGAACCCACTGCTTAAGCCTCAATAAAGCTTGCCTTGAGTGCTAAAGTGGTGTGT
176861_8776144_HIV1-ConN_1_2	119	239	TGCCCATCCATTCGGTAACTCTGGTACCTAGAGATCCCTCAGACCACCTAGACTGAGTGAAAAATCTCTAGCAGTGGCGCCCGAACAGGGACTTGAAAACGAAAGTAGAACCGGAGGCTG
176861_8776144_HIV1-ConN_1_3	238	358	GAATCTCTCGACGCAGGACTCGGCTCGTTGGTGCACACAGCGAGAGGCGAGGCGGCGGAAGTGTGAGTACGCAATTTTGACTGGCGGTGGCCAGAAAGTAGGAGAGAGGATGGGTGCGAG
176861_8776144_HIV1-ConN_1_4	357	477	GAGCGTCAGTGTTAACAGGGGGAAAATTAGATCAATGGGAAGCAATTTATTTGAGACCAGGGGGAAAGAAAAAATACAGAATGAAACATTTAGTATGGGCAAGCAGGGAGCTGGAAAGAT
176861_8776144_HIV1-ConN_1_5	476	596	TTCGCTTGTAACCCAGGTCTCATGGACACAGCGGACGGCTGTGCCCAGTTACTAAACCAATTAGAACCAGCTCTCAAGACAGGGTCAGAAGGACTGCGCTCTTTATATAACACCCTAGCA
176861_8776144_HIV1-ConN_1_6	595	715	AGTTCTTTATTGTGTCCATAGTAGAATACAGGTACACAATACACAAGAAGCTTTGGACAAGATAAAAGAGAAAAAGGAACAGCACAAGCCCGAGCCAAAAAACCCAGAAGCAGGGGCAGC
176861_8776144_HIV1-ConN_1_7	714	834	CGGCAGCAGCTGATAGCAATATCAGTAGGAATTATCCTCTAGTCCAGAATGCTCAAGGACAAATGGCACATCAGCCGCTGACACCCAGAACTTTAAATGCTTGGGTAAAAGTGATAGAGG
176861_8776144_HIV1-ConN_1_8	833	953	GAGAAGGCCTTTAATCCAGAAGTAATACCAATGTTTATGGCCTTGTCAGAAGGGGCAACACCCTCAGATCTAAATACTATGTTAAATACAGTAGGAGGACATCAGGCAGCTATGCAGATG
176861_8776144_HIV1-ConN_1_9	952	1072	GCTAAAGGAAGTCATCAATGAGGAAGCAGCAGAATGGGACAGGACGCATCCAGTCCCTGTGGGACCACTACCCCCAGGGCAACTGAGAGAACCTAGAGGAAGTGATATAGCAGGGACAAC
176861_8776144_HIV1-ConN_1_10	1071	1191	CTAGTACTCTGGCAGAACAGGTGGCATGGATGACTGCTAATCCTCCTGTTCCAGTAGGAGATATTTATAGAAGATGGATAGTTCTGGGGTTAAACAGAATTGTGAGAATGTATAGTCCTG
176861_8776144_HIV1-ConN_1_11	1190	1310	GTCAGCATTCTAGAGATCAAACAAGGACCAAAAGAACCCTTTAGAGACTATGTAGACAGGTTCTACAAAACTTTAAGAGCAGAGCAGGCAACACAGGAAGTAAAGAATTGGATGACAGAA
176861_8776144_HIV1-ConN_1_12	1309	1429	AACACTCTTAGTACAAAATGCAAACCCAGATTGTAAACAGATCCTAAAAGCATTAGGGCCAGGAGCTACCTTAGAAGAGATGATGACGGCCTGCCAGGGAGTGGGGGGACCAGCGCATAA
176861_8776144_HIV1-ConN_1_13	1428	1548	AGGCAAGAGTGCTAGCAGAGGCTATGTCACAGGTACAGGCAACAACTAGTGTCTTTGCACAAAGGGGAAACTTTAAAGGCATAAGGAAAACCATTAAATGTTTCAATTGTGGCAAAGAGG
176861_8776144_HIV1-ConN_1_14	1547	1667	GGCCATTTGGCAAGAAACTGTAAGGCCCCTAGAAGAGGGGGCTGTTGGAAGTGTGGGCAAGAAGGACATCAAATGAAAGATTGTAAAAATGAAGGAAGACAGGCTAATTTTTTAGGAAAG
176861_8776144_HIV1-ConN_1_15	1666	1786	GGGCTGGTCTCCCTTCAAAGGGAGACCAGGAAACTTCCCCCAGACAACAACAAGGAAAGAGCCCACAGCCCCGCCACTAGAGAATTATGGGTCTCAGGAGGAGAGGAGCACACAGGGGAA
176861_8776144_HIV1-ConN_1_16	1785	1905	AGGAGATGCAGGAGAACCAGGAGAAGACAGAGAACTCTCTGTACCCACCCTTAACTTCCCTCAGATCACTCTTTGGCAACGACCCGTCATCACAGTAAAAATAGGAAAGAAGTAAGAGA
176861_8776144_HIV1-ConN_1_17	1904	2024	AAGCTCTTTTAGATACAGGAGCTGATGATACAGTAATAGAAGAAATACAATTAGAAGGAAAATGGAAACCAAAAATGATAGGAGGAATTGGAGGATTTATCAAAGTAAGACAATATGATA
176861_8776144_HIV1-ConN_1_18	2023	2143	AATATAACAGTAGACATACAAGGAAGAAAAGCAGTTGGTACAGTGTTAGTAGGACCAACACCTGTTAATATTATAGGAAGAAACCTTTTAACACAGATTGGCTGTACTTTAAATTTTCCA
176861_8776144_HIV1-ConN_1_19	2142	2262	AATAAGTCCTATTGAAACTGTACCAGTAAAATTAAAACCAGGAATGGATGGCCCAAAGGTAAAACAATGGCCTTTGACAGCAGAAAAAATAGAGGCATTAAGAGAAATTTGTACAGAAAT
176861_8776144_HIV1-ConN_1_20	2261	2381	TGGAAAAGGAAGGAAAAATTTCTAGAATAGGGCCTGAGAATCCATATAACACTCCAATTTTTGCTATAAAAAAGAAAGATAGCACTAAATGGAGAAAATTAGTAGATTTCAGGGAATTAA
176861_8776144_HIV1-ConN_1_21	2380	2500	AATAAAAGGACCCAAGATTTTTGGGAAGTGCAGCTAGGAATTCCACATCCAGCAGGATTAAAGCAGAAAAAATCAGTGACAGTTTTAGATGTAGGAGATGCTTATTTTTCATGTCCCTTA
176861_8776144_HIV1-ConN_1_22	2499	2619	AGATGAAGATTTTAGAAAGTATACAGCTTTTACCATACCTAGTATAAATAATGAGACACCTGGTATTAGATACCAGTATAATGTGTTGCCACAGGGATGGAAGGGGTCACCAGCAATTTT

176861_8776144_HIV1-ConN_1_23	2618	2738	TTCAAAGTTCAATGACAAAAATTCTAGAACCATTTAGAAAGAAACATCCAGAGATAATCATTTATCAGTACATGGATGACCTCTATGTGGGATCTGACTTAGAACTAGCACAACATAGAG
176861_8776144_HIV1-ConN_1_24	2737	2857	GAGACAGTAGAAGAACTTAGAGGTCATCTTTTGAAGTGGGGCTTTACGACCCCTGACAAAAAACATCAAAAGGAGCCACCGTTCCTCTGGATGGGATATGAGCTCCATCCAGATAAATGG
176861_8776144_HIV1-ConN_1_25	2856	2976	GACGGTCCAGCCAATAAAGTTACCAGAAAAGGATGTATGGACTGTCAATGATATACAAAAATTAGTAGGAAAGTTAAATTGGGCAAGTCAGATCTATCCAGGAATCAAAGTAAAACAGCT
176861_8776144_HIV1-ConN_1_26	2975	3095	TTTGTAGATTAATCAGAGGAACCAAAGCTTTGACAGAAGTAGTCAACTTTACAAAAGAAGCAGAATTAGAACTAGCAGAAAACAGGGAGATATTAAAAGAACCCCTACATGGAGTCTATT
176861_8776144_HIV1-ConN_1_27	3094	3214	TATGACCCAGGAAAAGAATTAATAGCAGAAATTCAAAAACAAGGACAAGGTCAGTGGACATATCAGATTTATCAGGAGTCATATAAAAATTTAAAAACAGGAAAGTATGCAAAAATGAGA
176861_8776144_HIV1-ConN_1_28	3213	3333	ATCTGCCCATACTAATGATATAAAACAGTTAGTTGAAGTGGTACAAAAAGTGGCAACAGAAAGTATAGTAATTTGGGGAAAGACTCCTAAGTTTAAATTACCAGTACAAAAGGAAGTGTG
176861_8776144_HIV1-ConN_1_29	3332	3452	GGGAGGCATGGTGGACTGAGCATTGGCAAGCAACTTGGATTCCTGAGTGGGAATTCGTCAACACTCCTCCCCTTGTAAAATTATGGTATCAGTTAGAAACAGAGCCAATCAGTGGGGCAG
176861_8776144_HIV1-ConN_1_30	3451	3571	GAAACATACTATGTAGATGGAGCAGCTAATAGAGAAACAAAATTGGGAAAAGCAGGTTTTGTGACAGATAGAGGAAGACAGAAAGTGGTCTCTATTGAAGACACCACCAATCAAAAGGCT
176861_8776144_HIV1-ConN_1_31	3570	3690	TGAGTTACAAGCTATCCTTATGGCCTTACAAGAGTCAGGACAGGAAGTAAACATAGTCACTGACTCTCAGTATGCTATGGGAATAATTCACTCACAACCAGATAAAAGTGAATCAGAATT
176861_8776144_HIV1-ConN_1_32	3689	3809	TGGTGAGCCAAATAATAGAAGAACTCATAAAAAAGGAAAGAGTTTATCTCTCTTGGGTACCTGCACATAAAGGTATTGGAGGAAATGAGCAGGTAGACAAATTAGTTAGCTCAGGAATTA
176861_8776144_HIV1-ConN_1_33	3808	3928	AGAAAAGTATTATTCCTAGATGGTATAGAAAAAGCCCAAGAAGATCATGAAAGATATCACAGTAATTGGAGAGCAATGGCCAGTGATTTTAACTTACCCCCCATAGTGGCAAAAGAAATA
176861_8776144_HIV1-ConN_1_34	3927	4047	AGTAGCCAGCTGTGACAAATGCCAGCTAAAAGGGGAAGCCATGCATGGACAGGTCAATTGTAGTCCAGGAGTGTGGCAGTTAGATTGTACACACTTAGAAGGAAAAATCATTCTTGTAGC
176861_8776144_HIV1-ConN_1_35	4046	4166	CAGTCCATGTGGCCAGTGGCTACTTAGAAGCAGAAGTTATTCCTGCAGAAACAGGACAGGAAACAGCATATTTTATTTTAAAGTTAGCTGGAAGATGGCCAGTAAAAGTTATACACACTG
176861_8776144_HIV1-ConN_1_36	4165	4285	GATAATGGACCCAATTTCACTAGTGCCACTGTAAAAGCAGCCTGTTGGTGGGCAAATATCAAACAGGAATTCGGGATACCCTACAATCCTCAAAGTCAGGGAGCAGTAGAGTCCATGAAT
176861_8776144_HIV1-ConN_1_37	4284	4404	TAAAGAATTAAAGAAAATTATAGGACAAATCAGAGATCAAGCAGAACATCTAAAAACAGCAGTGCAAATGGCGGTTTTCATTCACAATTTTAAAAGAAAAGGGGGGATTGGGGGGTACAC
176861_8776144_HIV1-ConN_1_38	4403	4523	CTGCAGGGGAAAGAATAATAGACATAATAGCAACAGACATACAGACAACAAAATTACAAACACAAATTTTAAAAGTTCAAAATTTTCGGGTTTATTACAGAGACAGCAGAGATCCCATTT
176861_8776144_HIV1-ConN_1_39	4522	4642	TGGAAAGGACCAGCCAAACTTCTGTGGAAAGGAGAAGGGGCAGTGGTAATCCAAGATAACGGGGATATAAAAGTAGTCCCACGTAGGAAAGCAAAAATAATTAGGGATTATGGAAAACAG
176861_8776144_HIV1-ConN_1_40	4641	4761	GATGGCAGGTGATGGTTGTGTGGCAAGTGGACAGGATGAAAATCAGGACATGGAATAGCTTAGTAAAACATCATATGTATGTGTCAAAAAAGGCAAAAGGGTGGTATTATAGACATCATT
176861_8776144_HIV1-ConN_1_41	4760	4880	TATGAAACAAATCATCCAAAAATAAGTTCAGAAGTACATATCCCAGTAGGTCAGGCAAGATTAGTGATAATCACTTATTGGGGACTAACAACAGGAGAACAGCCTTGGCATCTAGGACAT
176861_8776144_HIV1-ConN_1_42	4879	4999	TGGAGTATCCATAGAATGGAGACTAAGAAAATACAAGACACAAGTTGATCCTGAAATGGCAGACAAGCTAATACATCTTTATTATTTTAATTGTTTTACAGCCTCTGCCATAAGGCAAGC
176861_8776144_HIV1-ConN_1_43	4998	5118	CGGTCTTAGGGAGACCAGTATTACCTAGGTGTGAATATCCAGCAGGGCACAAACAGGTAGGCACCTTACAATATCTAGCACTAACAGCCTGGGTGGGAGTAAAGAAGAGAAAGCCACCCT
176861_8776144_HIV1-ConN_1_44	5117	5237	TTACCTAGTGTGACTAAGTTAACAGAAGATAGATGGAACGAGCACCAGAAGACGCAGGGCCACAGAGGGAACCCTATAATGAATGGGCACTAGAATTATTAGAAGAATTAAAAAATGAAG
176861_8776144_HIV1-ConN_1_45	5236	5356	GCTGTGCGCCATTTTCCAAGGATTTGGCTACATGGGTTAGGACAACACATCTATAACACATATGGAGACACCTGGGAGGGGGTAGAGGCAATTATCAGGATACTACAACAATTACTGTTT
176861_8776144_HIV1-ConN_1_46	5355	5475	TATCCATTATAGGATTGGCTGCCAGCACAGCAGAATAGGGATTACTCCTCAAAGGAGAAGGAATGGAGCCAGTAGATCCTAGATTAGAGCCCTGGAATCATCCAGGAAGCCAACCCAAGA
176861_8776144_HIV1-ConN_1_47	5474	5594	ACAGCTTGCAATAATTGCTATTGTAAAAGATGTTGCTATCACTGTATGTGTTGCTTCACAAAGAAAGGCTTAGGCATCTCATATGGCAGGAAGAAGCGGAGTCAACGACGAAGAACTCCT
176861_8776144_HIV1-ConN_1_48	5593	5713	TAAGAGCAGTAAGAATCATCAAGATCCTATACCAGAGCAGTAAGTAAAAGCTGTATATATGCTGTCATTGGGATTCATAGCGTTAGGAGTAGCAATTATAATAGCAGTAATAATCTGGGT
176861_8776144_HIV1-ConN_1_49	5712	5832	TATTACTATATAAAGAATATAAGAAAATAAAATTGCAGGAAAAAATAAAACAAATAAGACAGAGAATAAGAGATAGAGCAGAAGATAGTGGCAATGAGAGTGATGGGGATGCAGAGTGGT
176861_8776144_HIV1-ConN_1_50	5831	5951	TTGGCCACTCTTCTGTCTCCTGATAAGCTTGATAATTGGGTCTGAACAACATTGGGTAACAGTGTACTATGGAGTACCAGTATGGAGAGACGCAGAGACAGTTCTTTTCTGCGCTTCAGA
176861_8776144_HIV1-ConN_1_51	5950	6070	ATGCTAAAGCCCATAGTACAGAGGCTCACAACATCTGGGCCACACAAGCATGTGTTCCCACTGATCCCAATCCACAAGAAGTGCTATTACCCAATGTAACTGAAAATTTTAATATGTGG
176861_8776144_HIV1-ConN_1_52	6069	6189	GAAAATAAAATGGCAGAACAAATGCAAGAGGACATTATCAGTTTATGGGAACAGAGCTTAAAGCCCTGTGTTAAATTAACCCCATTATGTGTAACTATGCTTTGTAACAATAGCAATGGG
176861_8776144_HIV1-ConN_1_53	6188	6308	GAATAACAATGAGACAGTTACAACAAGGAATTTAGACATAGGAGACAAACAAATGAAAAATTGCTCATTCAATGCAACCACTGAGATAAAAGATAAAAAGAAGCAAGTTTACTCTCTGTT
176861_8776144_HIV1-ConN_1_54	6307	6427	TTTATGCAGAAGATGTAGTACCAATCAATGATGCAACTAATAATAAAACATATAGGCTAATAAATTGTAATACCACAGCTGTGACACAAGCTTGTCCTAAGACCTCCTTTGAGCCAATTC
176861_8776144_HIV1-ConN_1_55	6426	6546	CCAATACATTACTGTGCACCACCAGGCTTTGCCATTATGAAATGTAATGAAGGAAACTTTAGTGGAAATGGAAAATGTAAAAATGTGAGTACTGTACAATGCACACATGGAATAAAGCCA
176861_8776144_HIV1-ConN_1_56	6545	6665	AGTGATATCCACTCAGTTAATCCTAAATGGAAGCTTAGATACAGATGATATTGTTATTAGAAATGATAGTGGTGGAAATCTGTTGGTGCAATGGAATGAGACAGTGTCAATAAATTGTAC
176861_8776144_HIV1-ConN_1_57	6664	6784	CAAGGCCAGGAAATAATACAGGAGGACAGGTGCAGATAGGACCTGCTATGACATTTTATAACATAGAGAAAATAGTAGGAGACATTAGACAAGCACACTGTAATGTCTCTAAAGAAGATT
176861_8776144_HIV1-ConN_1_58	6783	6903	TGGGAATCAATGTGGAATAGAACAAAAGAGAAAATAAAGAACATCCTGGGAAACAACATAACCTTCAAGGCTCGGGAGAAGAATGAAGGAGACCCAGAAGTAACACACTTAATGTTCAAT
176861_8776144_HIV1-ConN_1_59	6902	7022	TTGTGGAGGAGAGTTTTTCTATTGTAACACTTCCAAATTATTTAATGAGAGCAAGAACAAGACTACAAATGAGACTATTACTCTGCCCTGTAGAATAAAACAAATTGTAAATTTGTGGAC
176861_8776144_HIV1-ConN_1_60	7021	7141	CAAGAGTAGGAAAAGGAATTTATGCACCACCAATTCGGGGAAATCTTACCTGTAACTCCAATATTACTGGACTGATTCTAGAATATAGTGGTGGCAGTAATAATAGTACAGTATATCCCT
176861_8776144_HIV1-ConN_1_61	7140	7260	TCAGGAGGAAACATGGTTAATCTCTGGAGACAAGAGTTGTATAAGTACAAAGTAGTTAGTATAGAACCCATAGGAGTAGCACCAGGTAAAGCTAAAAGACGCACAGTGAGTAGAGAAAAG
176861_8776144_HIV1-ConN_1_62	7259	7379	GAGAGCAGCCTTTGGACTAGGTGCGCTGTTTCTTGGGTTTCTTGGAGCAGCAGGGAGCACTATGGGCGCAGCGTCAATAACGCTGACGGTACAGGCCCGGACATTATTATCTGGGATAGT
176861_8776144_HIV1-ConN_1_63	7378	7498	TGCAACAGCAGAATAATCTGTTGAGAGCAATAGAGGCGCAACAACATTTGTTGCAACTCTCAATCTGGGGCATTAAACAGCTCCAAGCAAAAGTCCTTGCTATAGAAAGATACCTTAGGG
176861_8776144_HIV1-ConN_1_64	7497	7617	GATCAGCAAATCCTAAGTCTATGGGGCTGCTCAGGAAAAACTATATGCTATACCACTGTGCCTTGGAATGAGACTTGGAGCAGTAATACCTCTTATGATACAATCTGGAATAATTTAACC
176861_8776144_HIV1-ConN_1_65	7616	7736	CTGGCAACAATGGGATAAGAAAGTAAGAAACTATTCAGGTGTCATTTTTGACCTTATAGAGCAGGCACAAGAACAACAGAACACAAATGAGAAATCACTCTTGGAATTGGATCAGTGGC
176861_8776144_HIV1-ConN_1_66	7735	7855	CAAGTCTGTGGAACTGGTTTGATATTACAAACTGGCTGTGGTACATAAAAATAGCTATAATGATAGTAGCAGGCATTATAGGCATAAGAATCATAAGTGTAATAATAACTATAATAGCAA
176861_8776144_HIV1-ConN_1_67	7854	7974	AGAGTTAGGCAGGGATATTCTCCCCTCTCGTTGCAGACCCTTATCCCAACAACAAGGGGACCAGACAGGCCAGAAGAAACAGAAGAAGGCGTTGGAGAGCAAGACAGAGGCAGATCCGTG
176861_8776144_HIV1-ConN_1_68	7973	8093	GCGATTAGTGAGCGGATTCTTAGCACTTGTCTGGGAGGACCTCAGGAACCTGTTGATCTTCCTCTACCACCGCTTGACAGACTCACTCTTGATCCTAAGGAGGACTCTGGAACTCCTGGG
176861_8776144_HIV1-ConN_1_69	8092	8212	GACAGAGTCTCAGCAGGGGACTGCAACTACTGAATGAACTCAGAATACACTTGTGGGGAATAATAGCATATTGGGGAAAAGAGTTAAAAGATAGTGCTATCAGCTTGCTTAATACAACAG
176861_8776144_HIV1-ConN_1_70	8211	8331	GCTATTGTAGTAGCAGAGGGAACAGATAGGCTTATAGAATTAGCACAAAGAATAGGAAGGGGAATATTACACATACCTAGAAGAATCAGACAGGGCCTAGAAAGAGCACTTGTATAAGAT
176861_8776144_HIV1-ConN_1_71	8330	8450	TGGGAAAGATTTGGTCAAAGAGCAGCATAGTAGGATGGCCAGAAATCAGAGAAAGAATGAGAAGACAAACTCAAGAGCCAGCAGTAGAACCAGCAGTAGGAGTAGGAGCAGCTTCTCAAG
176861_8776144_HIV1-ConN_1_72	8449	8569	GACCTAGCTAATCGAGGGGCCATCACCACAAGCAATACTAGAGACAATAATGAAACTGTAGCTTGGCTAGAAGCACAAGAAGAAGAAGAGGAAGTAGGCTTTCCAGTACGCCCTCAGGTA
176861_8776144_HIV1-ConN_1_73	8568	8688	ACCATTGAGGCCAATGACCTATAAAGCAGCTTTTGATCTTTCCTTCTTTTTAAAAGAAAAGGGGGGACTGGAAGGGCTAGTTTGGTCCAGAAAAAGGCAAGAGATTCTAGACCTCTGGGT
176861_8776144_HIV1-ConN_1_74	8687	8807	TTTATCACACACAAGGCTTCTTCCCTGACTGGCAGAACTACACACCAGGGCCAGGAGTTAGATACCCCCTGACCTTTGGATGGTGCTTCAAACTAGTACCATTGTCAGCTGAAGCAGTAG

176861_8776144_HIV1-ConN_1_75	8806	8926	GAAGAAGCTAATGAAGGAGACAACAATGCCCTCTTGCATCCCATATGTCAACATGGAGTAGATGATGATCACAAAGAAGTGCTGGTGTGGCGGTTTGACAGCTCCCTGGCAAGAAGACAC
176861_8776144_HIV1-ConN_1_76	8925	9045	CGTAGCAAGAGAGCTGCATCCGGACTTTTACAAGAACTGCTGACAAGGGACTTTACTGCTGACAAGGGACTTTACACTTGGGGACTTTCCGCCAGGGACTTTCCAGGGAGGTGTGGTTGG
176861_8776144_HIV1-ConN_1_77	9044	9164	GGGGAGTGGCTTGCCCTCAGAGCTGCATAAAAGCAGCCGCTTCTCGCTTGTACTGGGTCTCTCTTGCTGGACCAGATTAGAGTCTGGGAGCTCTCTGGCTAGCAGGGAACCCACTGCTTA
176861_8776144_HIV1-ConN_1_78	9163	9276	AAGCCTCAATAAAGCTTGCCTTGAGTGCTAAAGTGGTGTGTGCCCATCCATTCGGTAACTCTGGTACCTAGAGATCCCTCAGACCATCTAGACTGAGTGAAAAATCTCTAGCA
176861_8776145_N-5843-5988_1_1	1	120	AGAAGATAGTGGCAATGAGAGTGATGGGGATGCAGAGTGGTTGGGTGACAAGGAGAGTGTTGGGGATGATGAGTGGTTGGCCATTCTTCTGTCTCCTGATAAGCTCGATAATTGGGTCT
176861_8776146_N-5872-6021_1_1	1	120	TGGGGATGCAGAGTGGTTGGATGGGGATGAAGAGTGGTTGGTTACTCTTCTATCTTCTAGTAAGCTTGATCAAGGTAATTGGGTCTGAACAACATTGGGTAACAGTGTACTATGGGGTA
176861_8776147_N-6001-6120_1_1	1	120	TAACAGTGTACTATGGAGTACCAGTATGGAGAGACGCAGAGACAGTTCTTTTCTGCGCTTCAGATGCTAAAGCCCATAGTACAGAGGCTCACAACATCTGGGCCACACAAGCATGTGTT
176861_8776148_N-6161-6280_1_1	1	120	AACTGAAAATTTTAATATGTGGGAAAATAAAATGGCAGAACAAATGCAAGAGGACATTATCAGTTTATGGGAACAGAGCTTAAAGCCCTGTGTTAAATTAACCCCATTATGTGTAACTA
176861_8776149_N-6284-6528_1_1	0	120	TTTGTAACAATAGCAATGGGAATGTGACAGAGGGTAATGGAACTAACAGTAGCAGTGAAAATGTGGCAAGGAATAGTGAGACAAGGAAAACAAATAGCCTAGACATAGGAGACAAACAAA
176861_8776149_N-6284-6528_1_2	119	239	ATGAAAAATTGCTCATTCAATGCAACCACTGAGATAAAAGATAAAAAGAAGCAAGTTTACTCTCTGTTTTATGCAGAAGATGTAGTACCAATCAATGACAGTGACAGTGACAATAAAACA
176861_8776150_N-6601-6720_1_1	1	120	CAATTCCAATACATTACTGTGCACCACCAGGCTTTGCCATTATGAAATGTAATGAAGGAAACTTTAGTGGAAATGGAAAATGTAAAAATGTGAGTACTGTACAATGCACACATGGAATA
176861_8776151_N-6747-6866_1_1	1	120	CTAAATGGAAGCCTAGATACAGATGATATTGTTATTAGGAATAGTAGTGATAGCAATACGAGTGGTGATCTGTTGGTGCAATGGAATAAGACAGTGTCAATAAATTGTACAAGGCCAGG
176861_8776152_N-7277-7396_1_1	1	120	CAACATTACTGGACTGATTCTAGAATATAGTGAAGATAGTAAAGATGGCACTGGCACAAACACGACAGTATATCCCTCAGGAGGAAACATGGTTGATCTCTGGAGACAAGAGTTGCACA
176861_8776153_N-8581-8703_1_1	1	120	TAGTAGGATGGCCAGCAGTCAGAGAAAGAATGAGAAGGGCCAGACAAACTCAAGAAGCAGCAGCAGGAGCAGAAGCAGCAGTAGGAGTAGGAGCAGTTTCTCAAGACCTAGCTAATCGA
176861_8776154_N-8791-8910_1_1	1	120	GCTTTCCAGTACGCCCTCAGGTACCATTGAGGCCAATGACCTATAAAGCAGCTTTTGATCTTTCCTTCTTTTTAAAAGAAAAGGGGGGACTGGAAGGGCTAGTTTGGTCCAGAAAAAGG
176861_8776155_HIV1-Con_O_1_1	0	120	TGGATGGGTTAATTTACTCCCATAAGAGAGCAGAGATCCTGGATCTTTGGGTGTATCACACTCAGGGATTCTTCCCTGATTGGCAGAACTACACACCAGGACCAGGAACCAGATTCCCAC
176861_8776155_HIV1-Con_O_1_2	119	239	CTGACATTTGGATGGTTATTTAAGCTAGTACCAGTGTCAGAAGATGAGGCAGAAAGACTAGGAAATAAGTGTGAGAGGGCTAGTCTCCTGCATCCAGCTTGTAACCATGGCTTTGAAGAT
176861_8776155_HIV1-Con_O_1_3	238	358	TCAACACGGGGAAATACTGAAATGGCAGTTTGATAGATCACTAGGCAACACCCATGTTGCTATGATAACTCACCCAGAGCTCTTCCCCAAGGACTAAAACTGCTGACCTGAAGATTGCTG
176861_8776155_HIV1-Con_O_1_4	357	477	GACACTGCGGGACTTTCCAGCAAAGACTGCTGACACTGCGGGGACTTTCCAGCGTGGGAGGGATAGGGGCGGTTCGGGGAGTGGCTAACCCTCAGATGCTGCATATAAGCAGCCGCTTTC
176861_8776155_HIV1-Con_O_1_5	476	596	CTGCTTGTACCGGGTCTCGGTTAGAGGACCAGGTCTGAGCCCGGGAGCTCCCTGGCCTCTAGCTGAACCCGCTGCTTAACGCTCAATAAAGCTTGCCTTGAGTGAGAAGCAGTGTGTGCT
176861_8776155_HIV1-Con_O_1_6	595	715	TCATCTGTTCAACCCTGGTATCTAGAGATCCCTCAGATCACGTAGACTGAAGCAGAAAATCTCTAGCAGTGGCGCCCGAACAGGGACTTGAAAGAGAAAGTGAAACCAGGGAAGAAAACC
176861_8776155_HIV1-Con_O_1_7	714	834	CTCCGACGCAACGGGCTCGGCTTAGCGGAGTGCACCCGCTAAGAGGCGAGAGGAACTCACAGAGGGTGAGTAAATTTGCTGGCGGTGGCCAGACCTAGGGGAAGGGCGAAGTCTCTAGGG
176861_8776155_HIV1-Con_O_1_8	833	953	GGAGGAAGATGGGTGCGAGAGCGTCTGTGTTGACAGGGAGCAAATTGGATGCATGGGAACAAATTAGGTTAAAGCCAGGATCTAAAAAGAAATATAGGCTAAAACATTTAGTATGGGCAA
176861_8776155_HIV1-Con_O_1_9	952	1072	AGCAGGGAGCTGGAAAGATTCGCATGTAATCCTGAGCTACTAGAAACTGCAGAGGGTAATGAGAAACTGTTACAGCAGTTAGAGCCAGCTCTCAAGACAGGGTCAGAAAGCCTGCAGTCA
176861_8776155_HIV1-Con_O_1_10	1071	1191	ACTCTGGAACACAATAGCAGTGCTCTGGTGTGTTCACAACAGATATAAAGTTGAAGATACGCAGCAGGCAATACAAAAGTTAAAGGAAGTAATGGGGAGCAGGAAGTCTGCAGGTGCCGC
176861_8776155_HIV1-Con_O_1_11	1190	1310	CTAAGGAAGACACAAGCGCAAGGCAGACAGGTCAAAATTACCCTGTAGTACCAAATGCGCAGGGACAAATGGTACATCAGGCCCTCTCCCCCAGGACTTTAAATGCATGGGTAAAGGCAG
176861_8776155_HIV1-Con_O_1_12	1309	1429	GTAGAAGAAAAGGCCTTTAACCCTGAAATCATTCCTATGTTCATGGCATTGTCAGAGGGAGCTATTCCCTATGATATTAATACTATGCTAAATGCCATAGGAGGACATCAAGGGGCTTTA
176861_8776155_HIV1-Con_O_1_13	1428	1548	ACAAGTGCTAAAGGAAGTAATCAATGAGGAAGCAGCAGATTGGGATAGAACTCACCCACCAGCGGTAGGGCCGTTACCACCAGGGCAGATAAGGGAACCAACAGGAAGTGACATTGCTGG
176861_8776155_HIV1-Con_O_1_14	1547	1667	GAACAACTAGCACCCAGCAAGAGCAAGTTCACTGGATTACCAGGGCCAACAACCCTATCCCAGTAGGAGACATCTATAGAAAATGGATAGTGTTAGGACTAAACAAAATGGTAAAAATGT
176861_8776155_HIV1-Con_O_1_15	1666	1786	TACAGTCCAGTGAGCATCTTAGATATTAAACAGGGACCAAAAGAACCATTTAGAGACTATGTAGATCGGTTCTACAAAACATTAAGAGCTGAGCAAGCAACTCAAGAAGTAAAAAATTGG
176861_8776155_HIV1-Con_O_1_16	1785	1905	GATGACAGAAACCCTGCTTGTTCAAAATGCCAACCCAGATTGCAAACAGATTTTGAAATCATTAGGGCCAGGAGCTACCTTAGAAGAGATGATGGTAGCCTGTCAGGGAGTAGGAGGACC
176861_8776155_HIV1-Con_O_1_17	1904	2024	CAACTCATAAGGCCAGAGTACTAGCAGAAGCAATGGCTACAGCCCAGCAAGATCTAAAAGGAGGATACACAGCAGTATTCATGCAAAGAGGGCAAAACCCAATTAGGAAAGGGCCTATAA
176861_8776155_HIV1-Con_O_1_18	2023	2143	AAATGTTTCAACTGTGGAAAAGAGGGACATATAGCAAAAAATTGTCGAGCACCTAGAAAGAAAGGTTGCTGGAAATGTGGACAGGAAGGTCACCAAATGAAAGATTGCAGAAATGGAAAA
176861_8776155_HIV1-Con_O_1_19	2142	2262	ACAGGCAAATTTTTTAGGCAAATACTGGCCTCCGGGGGGCACGAGGCCAGGCAATTATGTGCAGAAACAAGTGCCCCCATCAGCCCCACCAATGGAGGAGGTAATGAAGGGACAAGAGAA
176861_8776155_HIV1-Con_O_1_20	2261	2381	ATCAGGAACAGAAGGGGGACCCGAACGAACTCTATCCGTTTGCCTCCCTCAAATCCCTCTTTGGGACAGACCAGTAGTCACAGCAAAGGTTGGGGGCCATCTATGTGAAGTTTTACTGGA
176861_8776155_HIV1-Con_O_1_21	2380	2500	ATACAGGGGCAGATGATACAGTACTAAACAACATACAATTGGAAGGAAAATGGACACCAAAAATGATAGGGGGTATAGGAGGTTTTATAAAAGTAAAAGAATATAATAATGTGACAGTAG
176861_8776155_HIV1-Con_O_1_22	2499	2619	GAAATAGAAGGAAGGGAAGTACAGGGAACAGTATTGGTGGGACCTACTCCTGTTAATATTATTGGAAGAAATATATTGACAGGATTAGGTTGTACACTAAATTTCCCTATAAGCCCCATA
176861_8776155_HIV1-Con_O_1_23	2618	2738	AGCCCCAGTACCAGTAAAACTAAAACCAGGAATGGATGGACCAAAAGTAAAACAATGGCCCCTATCTAAAGAAAAAATAGAAGCCTTGACAGCAATATGTCAGGAAATGGAACAAGAAGG
176861_8776155_HIV1-Con_O_1_24	2737	2857	GAAAAATTTCAAGAATAGGACCTGAAAATCCTTATAATACACCTATCTTTGCTATAAAAAAGAAAGATAGTACTAAGTGGAGAAAATTGGTAGACTTTAGAGAATTAAACAAGAGAACAC
176861_8776155_HIV1-Con_O_1_25	2856	2976	CAAGATTTCTGGGAGGTACAGTTAGGTATCCCACATCCGGGGGGTTTAAAGCAAAAGCAATCTGTTACAGTCTTAGATGTAGGAGATGCTTATTTCTCATGCCCTTTAGACCCAGATTTC
176861_8776155_HIV1-Con_O_1_26	2975	3095	CAGAAAATATACTGCTTTCACTATTCCTAGTGTGAACAATGAGACCCCAGGAATAAGATACCAGTACAATGTCCTCCCGCAAGGATGGAAAGGTTCACCAGCTATATTCCAAAGTTCAAT
176861_8776155_HIV1-Con_O_1_27	3094	3214	TGACAAAAATTCTAGATCCATTTAGGAAAGACAACCCAGAATTAGAAATTTGTCAGTACATGGATGACTTATATGTAGGATCAGATTTACCCTTGACAGAACATAGAAAAAGGGTTGAAT
176861_8776155_HIV1-Con_O_1_28	3213	3333	TTGCTTAGAGAACATTTATATCAGTGGGGATTCACTACCCCTGATAAAAAGCATCAAAAGGAACCTCCCTTTCTGTGGATGGGGTATGAGCTCCATCCAGACAAATGGACAGTACAGCCC
176861_8776155_HIV1-Con_O_1_29	3332	3452	CATCCAATTGCCTAACAAGGATGTGTGGACAGTAAATGATATACAAAAACTAGTAGGAAAGTTAAATTGGGCAAGTCAAATCTATCAAGGAATTAGAGTAAAAGAATTGTGTAAGTTAAT
176861_8776155_HIV1-Con_O_1_30	3451	3571	TTAGAGGCACCAAGTCATTGACAGAAGTAGTACCTTTAAGTAAAGAGGCAGAGCTAGAATTAGAAGAAAACAGAGAAAAGCTAAAAGAACCAGTGCATGGTGTATACTATCAACCTGACA
176861_8776155_HIV1-Con_O_1_31	3570	3690	AAAGACTTATGGGTTAATATTCAGAAGCAAGGAGAAGGGCAATGGACTTACCAGATATATCAGGATGAACATAAGAACCTCAAAACAGGGAAATATACTAGGCAAAAGGCCTCCCACACA
176861_8776155_HIV1-Con_O_1_32	3689	3809	AAATGATATAAGACAATTAGCAGAAGTAGTCCAGAAGGTGTCTCAAGAAGCTATAGTTATCTGGGGAAAATTGCCTAAATTTAAGCTGCCAGTCACTAGAGAAACTTGGGAAACATGGTG
176861_8776155_HIV1-Con_O_1_33	3808	3928	GGGCAGACTATTGGCAAGCCACCTGGATTCCAGAATGGGAATTTGTCAGCACACCCCCATTGATCAAATTATGGTACCAGTTAGAAAGTGAACCTATTATGGGGGCAGAAACCTATTATG
176861_8776155_HIV1-Con_O_1_34	3927	4047	GTAGATGGAGCAGCTAATAGAGATACAAAACTAGGAAAGGCAGGATATGTTACAGAACAAGGGAAACAGAAAATAATAAAATTAGAGGAGACCACCAATCAAAAGGCTGAATTAATGGCA
176861_8776155_HIV1-Con_O_1_35	4046	4166	AGTATTATTAGCCTTACAGGATTCCAAAGAAAAAGTAAATATAGTAACAGATTCACAATATGCATTGGGCATCATCTCCTCCCAACCTACACAGAGTGAATCCCCTATAGTTCAGCAGAT
176861_8776155_HIV1-Con_O_1_36	4165	4285	TAATAGAGGAACTAACAAAAAAGGAACAGGTGTATCTTACATGGGTTCCTGCTCATAAAGGCATAGGAGGAAATGAAAAAATAGATAAATTAGTAAGCAAGGATATTAGAAGAGTCCTAT
176861_8776155_HIV1-Con_O_1_37	4284	4404	TTCCTAGAAGGAATAGACCAGGCACAAGAAGATCATGAAAAATATCATAGCAATTGGAGAGCATTAGCTAGTGACTTTGGACTACCACCAGTAGTGGCCAAGGAAATCATTGCTAGTTGT

176861_8776155_HIV1-Con_O_1_38	4403	4523	TCCTAAATGTCATATAAAAGGGGAAGCAATTCATGGTCAGGTAGACTGCAGTCCAGAAGTATGGCAAATGGATTGCACACATTTAGAAGGCAAAGTCATAATAGTTGCTGTCCATGTGGC
176861_8776155_HIV1-Con_O_1_39	4522	4642	CAAGTGGATTCATAGAAGCAGAAGTGATACCAGCAGAAACAGGACAAGAAACTGCCTACTTCCTGTTAAAATTAGCAGCAAGATGGCCTGTTAAAGTAATACATACAGACAATGGGCCTA
176861_8776155_HIV1-Con_O_1_40	4641	4761	AATTTTACAAGTGCAACCATGAAGGCTGCATGTTGGTGGACCAACATAAAACATGAGTTTGGAATACCATATAATCCACAAAGTCAAGGAGTAGTAGAAGCCATGAATAAGGAATTAAAA
176861_8776155_HIV1-Con_O_1_41	4760	4880	ATCAATTATACAGCAGGTGAGGGACCAAGCAGAACACTTAAAAACAGCAGTACAAATGGCAGTATTTGTTCACAATTTTAAAAGAAAAGGGGGGATTGGGGGGTACACTGCAGGAGAAAG
176861_8776155_HIV1-Con_O_1_42	4879	4999	GGATAATAGACATATTAGCATCACAAATACAAACAACAGAACTACAAAAACAAATTTTAAAAATTCAAAATTTTCGGGTCTATTACAGAGACAGCAGAGACCCTATTTGGAAAGGACCGG
176861_8776155_HIV1-Con_O_1_43	4998	5118	GCACAGCTCCTGTGGAAAGGTGAGGGAGCAGTAGTCATACAAGATAAAGGAGACATTAAGGTAGTACCAAGAAGAAAGGCAAAAATAATCAGAGATTATGGAAAACAGATGGCAGGTACT
176861_8776155_HIV1-Con_O_1_44	5117	5237	TGATAGTATGGCAAGTGGACAGACAGAAAGTGAAAGCGTGGAACAGCCTGGTGAAATACCATAAGTACAGGTCTAGGAAGACCAAGGACTGGTATTACAGACATCATTATGAATCTAGAA
176861_8776155_HIV1-Con_O_1_45	5236	5356	AATCCAAGAGTCAGTTCAGGTGTATATATTCCAGTAGGGGCAGCTTGTATAGTAGTGACCACATATTGGGGATTGATGCCAGGGGAAAGAGATGAACATTTGGGACATGGGGTTAGTATA
176861_8776155_HIV1-Con_O_1_46	5355	5475	AGAATGGCAGTACAAGAAGTATAAAACACAGATTGACCCTGAAACAGCAGACAGGATGATACATCTGTATTATTTCACCTGTTTTACAGAGTCAGCAATCAGGAAAGCCATCCTAGGGCA
176861_8776155_HIV1-Con_O_1_47	5474	5594	AGAGAGTACTGACCAAGTGTGAATACCCTGCAGGACATAGTCAGGTAGGGACACTGCAACTACTAGCTCTAAGAGCAGTAGTAAAAGAAAAAAGAAATAAGCCTCCCCTACCCAGTGTCC
176861_8776155_HIV1-Con_O_1_48	5593	5713	CAGAAATTAACAGAAGATAGATGGAACAGGCACCTGAGGATCAGGGACCAGCTAGAGAGCCATTCAATGAATGGGCACTAGAGCTCCTAGAAGAGCTAAAAGCAGAAGCAGTAAGACATT
176861_8776155_HIV1-Con_O_1_49	5712	5832	TTCCCTAGGCCTTGGCTACAAGCCTTGGGACAATACATTTATGAGACTTATGGAGACACTTGGGTAGGAGTTATGGCAATTATAAGAATCTTACAACAACTGCTATTTACCCATTTTAGA
176861_8776155_HIV1-Con_O_1_50	5831	5951	AATTGGATGCCAACATAGTAGAATAGGAATTAACCCATCTAACACAAGAGGAAGAGGAAGAAGAAATGGATCCAGTAGATCCTGAGATGCCCCCTTGGCATCACCCTGGAAGTCAGCCCC
176861_8776155_HIV1-Con_O_1_51	5950	6070	CAGACCCCTTGTAATAATTGCTATTGCAAAAAATGCTGCTATCATTGCTATGTTTGTTTCACAAGCAAGGGTTTGGGAATCTCCTATGGCAGGAAGAAGCGACGACGACCAGCAGCTGCT
176861_8776155_HIV1-Con_O_1_52	6069	6189	TGCAAGCCATCCAGATAATAAAGATCCTGTACCAGAGCAGTAAGTAACGCTAATGCATCATAGGGACCTGCTAATATTAATAATTATTAGTGCTTTGCTGCTTATAAATGTAATTCTATG
176861_8776155_HIV1-Con_O_1_53	6188	6308	GGATGTTTATTCTTAGAAAATATTTAGAACAAAAGAAACAAGACAGAAGGGAAAGAGAAATACTTGAAAGGTTAAGAAGAATAAGAGAAATTAGGGATGACAGTGACTATGAAAGCAATG
176861_8776155_HIV1-Con_O_1_54	6307	6427	GGAGAAGAAGAACAAGAAGTTATGGATCTTGTGCATAGTCATGGCTTTGATAATCCCATGTTTGAGCTCTAACCAGCTGTATGCAACAGTCTATGCTGGGGTGCCTGTATGGGAAGATGC
176861_8776155_HIV1-Con_O_1_55	6426	6546	CAACCCCAGTATTATTCTGTGCTTCAGATGCTAACCTAACAAGCACTGAGCAGCATAATATTTGGGCATCACAAGCCTGTGTTCCCACAGACCCCACTCCACATGAATATCCATTAAACA
176861_8776155_HIV1-Con_O_1_56	6545	6665	AATGTGACAGATAACTTTAATATATGGAAAAATTACATGGTAGAACAAATGCAGGAAGACATTATTAGTTTATGGGACAGAGTTTAAAACCTTGTGTTCAAATGACTTTCCTGTGTGTA
176861_8776155_HIV1-Con_O_1_57	6664	6784	ACAAATGAATTGTACAGACGTAGCTGGAACAACAAATGAAAACCCTATGAAAAAGTGTGAGTTTAATGTAACCACAGTTCTCAAAGACAAAAAGGAGAAAAAACAGGCTCTATTCTATGT
176861_8776155_HIV1-Con_O_1_58	6783	6903	TATCAGATTTGATTAAAATGAATGAGACAAGCAGCACAAATAAGACAAACAGCAAAATGTATACATTAATTAATTGTAACTCCACAACCATCAAGCAAGCCTGTCCAAAGGTATCTTTTG
176861_8776155_HIV1-Con_O_1_59	6902	7022	GAGCCAATTCCAATACACTATTGTGCTCCAGCAGGATATGCCATCTTTAAGTGTAACAACACAGAATTTAATGGAACAGGCACATGCAACAACATTACAGTAGTTACTTGTACACATGGC
176861_8776155_HIV1-Con_O_1_60	7021	7141	CATCAAGCCAACAGTAAGTACTCAACTAATATTAAATGGGACACTCTCTAAAGGAAAAATAAGAATTATGGGAAAAAATATTTCAGACAGTGGAAAAAATATCATAGTGACCCTAAATTC
176861_8776155_HIV1-Con_O_1_61	7140	7260	CTACTATAAACATGACCTGTGAGAGACCAGGAAATCAGACAGTACAAGAGATAAAAATAGGTCCAATGGCCTGGTACAGCATGGGCCTAGAGAAAGACACAAACACATCCAACTCAAGGA
176861_8776155_HIV1-Con_O_1_62	7259	7379	ATAGCTTATTGCAAGTATAATACCACTGATTGGGAAAAAGCCTTAAAACAAACAGCTGAAAGGTATTTAGAACTTGTAAACAATACAGATAATGTTAGCATAATATTCAATAGCAGCACT
176861_8776155_HIV1-Con_O_1_63	7378	7498	TGGTGGAGATCCAGAGATAACCCATTTGCATTTTAACTGTCATGGAGAATTCTTTTATTGTAACACATCTAAGATGTTTAATTATACCTTTACATGTAAAGGAACCAACTGTAATAGTAA
176861_8776155_HIV1-Con_O_1_64	7497	7617	ATGTTAGTCAAGGTAACAATGGCAATAATAAGATACCTTGCAGGATGAAACAGGTAGTAAGGTCATGGATGAGGGGAGGGTCAGGACTCTATGCACCTCCCATCAAAGGTAACCTAACAT
176861_8776155_HIV1-Con_O_1_65	7616	7736	TGTAGATCAAACATAACTGGAATGATTCTACAAATGGATAAGCCATGGAACAACAGCAACAACAACAATGCCACATTTAGACCAATAGGGGGAGATATGAAAGATATATGGAGAACTGAATTG
176861_8776155_HIV1-Con_O_1_66	7735	7855	GTTCAACTACAAAGTAGTAAGGGTAAAACCTTTTAGTGTGGCACCTACAAAAATTGCAAGGCCAGTCATAGGCACTGGCACTCATAGAGAAAAAAGAGCAGTAGGATTGGGAATGCTATT
176861_8776155_HIV1-Con_O_1_67	7854	7974	TCTTGGGGGTTCTAAGTGCAGCAGGTAGCACTATGGGCGCAGCGGCAACAGCGCTGACGGTACAGACCCACACTTTGATAAAGGGTATAGTGCAACAGCAGGACAACCTGCTAAGAGCAA
176861_8776155_HIV1-Con_O_1_68	7973	8093	ATACAGGCCCAGCAGCAATTGCTGAGGCTATCTGTATGGGGTATCAGACAACTCCGAGCTCGCCTGCTAGCCTTAGAAACCTTAATACAGAATCAGCAACTCCTAAACCTATGGGGCTGT
176861_8776155_HIV1-Con_O_1_69	8092	8212	TAAGGGAAGGCTAATCTGCTACACATCAGTAAAATGGAACAAAACATGGATAGGAAATGAAACAATTTGGGACAACCTAACATGGCAGGAATGGGATCAGCAGATAAACAACATAAGCTC
176861_8776155_HIV1-Con_O_1_70	8211	8331	CCATCATATATGATGAAATACAAAAGGCACAAGTACAGCAGGAACAAAATGAGAAAAAGTTGCTGGAGTTAGATGAATGGGCTTCTATTTGGAATTGGCTTGACATAACTAAATGGTTGT
176861_8776155_HIV1-Con_O_1_71	8330	8450	TGGTATATAAAAATAGCAATAATCATAGTAGGAGCACTAATAGGTGTGAGAATTGTCATGATAGTACTTAATCTAGTGAGAAACATTAGGCAGGGATATCAACCCCTCTCGTTACAGATC
176861_8776155_HIV1-Con_O_1_72	8449	8569	CCCCATCCATCACCAACCGGAAGCAGAAACGCCAGGAGGAACAGGAGAAGGAGGTGGAGAAGAAGGCAGGCCCAGGTTGATACCCTCGCCGCAAGGATTCTTGCCACTGTTGTACACGGA
176861_8776155_HIV1-Con_O_1_73	8568	8688	ACCTCAGGACAATAATCTTGTGGAGTTACCACCTCTTGAGCAACTTAGCATCAGGGATCCAGAAGGTGATCAGCCATCTGGGACTTGGACTGTGGATCCTAGGGCAGAAGATAATTGATG
176861_8776155_HIV1-Con_O_1_74	8687	8807	GCTTGCAGAATTTGTGCAGCTGTAATACAATACTGGCTACAAGAATTGCAGAATAGTGCTACAAGTCTACTAGATACCATTGCAGTGGCAGTTGCCAATTGGACTGACAGCATAATCTTA
176861_8776155_HIV1-Con_O_1_75	8806	8926	AGGGATACAAAGAATAGGAAGAGGAATTCTTAACATCCCAAGAAGAATTAGACAGGGCTTAGAAAGAAGTTTATTGTAACATGGGAAACGCATTGAGAAAAAGTAAATTTGCAGGATGGT
176861_8776155_HIV1-Con_O_1_76	8925	9045	TCAGCAGTAAGAGAAAGAATGAGAAAAACTTCCCCTGATCCTGAACCATGTGCACCTGGAGTAGGAGAAGTCTCCAGGGAATTAGCAGCTAGAGGAGGGATACCAAGTTCCTATACTCCT
176861_8776155_HIV1-Con_O_1_77	9044	9164	TCAAAACAATGCAGCCCTTGCATTCCTAGAAAGTCACCAAGATGAAGAAGTAGGTTTCCCAGTAAGACCTCAAGTGCCTCTAAGGCCAATGACCTATAAAGGAGCATTTGACCTCAGCTT
176861_8776155_HIV1-Con_O_1_78	9163	9283	TCTTTTTAAAAGAAAAGGGAGGACTGGATGGGTTAATTTACTCCCATAAAAGAGCAGAGATCCTGGATCTTTGGGTGTATCACACTCAGGGATTCTTCCCTGATTGGCAGAACTACACAC
176861_8776155_HIV1-Con_O_1_79	9282	9402	CCAGGACCAGGAACTAGATTCCCACTGACATTTGGATGGCTATTTAAGCTAGTACCAGTGTCAGAAGATGAGGCAGAAAGACTAGGAAATAAGTGTGAGAGGGCTAGTCTCCTGCATCCA
176861_8776155_HIV1-Con_O_1_80	9401	9521	AGCTTGTAACCATGGCTTTGAAGATCCACACGGGGAAATACTGAAATGGCAGTTTGATAGATCACTAGGCAACACCCATGTTGCTATGATAACTCACCCAGAGCTCTTCCACAAGGACTA
176861_8776155_HIV1-Con_O_1_81	9520	9640	AAAACTGCTGACCTGAAGATTGCTGACACTGCGGGACTTTCCAGCAGAGACTGCTGACACTGCGGGGACTTTCCAGCGTGGGAGGGATAAGGGGCGGTTCGGGGAGTGGCTAACCCTCAG
176861_8776155_HIV1-Con_O_1_82	9639	9759	GATGCTGCATATAAGCAGCCGCTTTCTGCTTGTACCGGGTCTCGGTTAGAGGACCAGGTCTGAGCCCGGGAGCTCCCTGGCCTCTAGCTGAACCCGCTGCTTAACGCTCAATAAAGCTTG
176861_8776155_HIV1-Con_O_1_83	9758	9851	GCCTTGAGTGAGAAGCAGTGTGTGCTCATCTGTTCAACCCTGGTATCTAGAGATCCCTCAGATCACGTAGACTGAAGCAGAAAATCTCTAGCA
176861_8776156_ConO_6740-6859_1_1	1	120	TGTGACAGATAACTTTAATATATGGAAAAATTACATGGTAGAACAAATGCAGGAAGACATTATTAGTTTATGGGACAGAGTTTAAAACCTTGTGTTCAAATGACTTTCCTGTGTGTAC
176861_8776157_ConO_6856-7154_1_1	0	120	GTACAAATGAACTGTACAGATCCGAAAATTGATAGTACAGGGAATAATAGTACAGGGAAGAATAGTACAGGGAATAATAGTACAGTAGATAGCACGACAGAACCAGGAACACCTATGAAA
176861_8776157_ConO_6856-7154_1_2	119	239	AAACTGTAGTTTTAATATAACTACATTCCTCAAAGACAAAAAGGAGCAAAAACAGGCTCTATTCTATATATCAGATCTGACTAGGGTTAATGGAACTAATGGAACAAATGACACAATATA
176861_8776158_ConO_6856-7217_1_1	0	120	GTACAAATGAATTGTACAGACATAGAAAACAAAAACAGTAGCTCAGAAAACCCTATGAAAAAGTGTGAGTTTAATATAACCACAGTTCTCAAGGACAAAAAGGAGAAAAAACAGGCTTTA
176861_8776158_ConO_6856-7217_1_2	119	239	ATTCTATAGAGCAGATTTGACTGAAATAAGAGACAACAGCACAAACAGCACAATATATACATTAATTAATTGTAATTCCACAACCATTAAGCAAGCCTGTCCAAAGGTATCTTTTGAGCC
176861_8776159_ConO_6859-7151_1_1	1	121	AAATGAATTGCACAGATTATAACATAACCATCAATGCAACCAACTCTAACTCCAATGACAGCTCAAGGGTAGCCACCTCCAATGTAACCACCGTCAATGTAACCACCGATCTAAAACAGT

176861_8776159_ConO_6859-7151_1_2	121	240	GTGACTTTAATGTAACCACAGTTGTCAAAGACAAAAAGGAGCAAAAACAGGCTCTATTCTATAAATCAGATTTGATGGAAATAACAGATAAAAATAAGAAAAACAATACACAGAAAATC
176861_8776160_ConO_6864-7153_1_1	0	120	GAATTGTACAGACTTTAAAACAAACGACCCCTCTACCTCCCCACCTACCTCGCTCCCTAGCGGTTCAGAGAACCCTATGAAAAAATGTGAGTTTAATGTAACCACAGTTCTCAAAGACAA
176861_8776160_ConO_6864-7153_1_2	119	239	AAAAGGAGAAAAAACAGGCTCTATTCTATATATCAGATTTGGTTAAAGTGAATGATAGCGAAACAAGTAAAGGGAATAATAGCGAAACAAGTGGCAATAACACAACACCATCAGGAATAT
176861_8776161_ConO_7161-7280_1_1	1	120	ATTAATTGTAACTCCACAACCATCAAGCAAGCCTGTCCAAAGGTATCTTTTGAGCCAATTCCAATACACTATTGTGCTCCAGCAGGATATGCCATCTTTAAGTGTAACAACACAGAATT
176861_8776162_ConO_7280-7399_1_1	1	120	TAATGGAACAGGCACATGCAACAACATTACAGTAGTTACTTGTACACATGGCATCAAGCCAACAGTAAGTACTCAACTAATATTAAATGGGACACTCTCTAAAGGAAAAATAAGAATTA
176861_8776163_ConO_7534-7790_1_1	0	120	TGGGTCAGTATGGCAGCTAAAACAGAGTCACAGAACACAGGGTCAAGGATAGCTTATTGTATGTATAATAACACTGAATGGATAAATACCTTAAAACAAACAGCTGAAAGATATTTAGAA
176861_8776163_ConO_7534-7790_1_2	119	239	ACTCGTAAACAAGACAGGAAATTTAAGCAAGACAGGAAATTTTAGCATAATATTCAACCACAGTATAAGTGGTGGAGACATAGAAGCAAGCTCTTTACATTTTAACTGTCATGGAGAATT
176861_8776164_ConO_7534-7799_1_1	0	120	TGGCTCAGCATGGGCATAAGGGAAGAAAGTAAAAATACAAATGCCTCAAGGAGGGCTTATTGCAAATTTAATACCAGTGCCTGGATAGAAACCTTAAAAGGAACAGCTGAAAGGTATCTA
176861_8776164_ConO_7534-7799_1_2	119	239	AGAGCTTGTAGAAGCAAATAAAACAGAGAATATTAGCATGATGTTTGATAGAAGTGATCTTGGTGGAGATCCAGAGATAACCCATTTGCATTTTAACTGTCATGGAGAGTTCTTTTATTG
176861_8776165_ConO_7825-8099_1_1	0	120	ACCTTTACATGTAACAAAACCAACTGCACTAACAATAGGACTGATAATAGCACTAATGACACAATACCTTGCAAGTTGAGACAGGTAGTGAGGTCATGGATACAGGGGGGGTCAGGACTC
176861_8776165_ConO_7825-8099_1_2	119	239	CTATGCCCCTCCCATCAAAGGTACCCTAACATGTATGTCAAACATAACTGGGATGATTCTAGAAATGGAACGGTCATGGAACGGGAGTGCTACAAAGGACATTAATGCTACATGGAACAT
176861_8776166_ConO_7825-8102_1_1	0	120	AACTTTTTATGTAACAAAACCAACTGTACAAATAGCCAAAATAACAGTCAAAGTTACAATAGCACTGAGAGACTGAAATTGGGTTTGATACCTTGCAAAATAAAACAGGTAGTAAGGTCA
176861_8776166_ConO_7825-8102_1_2	119	239	ATGGATGAAGGGAGAGTCAGGACTCTATGCACCTCCCATCCCAGGTAATCTAACATGTCAGTCAAACATAACTGGAATGATTCTACAAATAGATGTGCCATGGAATAAAAGCCATGGCAA
176861_8776167_ConO_7831-8099_1_1	0	120	TCTTGTAACAATTCCACCTGTCAGAACATGTCTGGCAGTGACACTTCTAGCAATGACACTATACCTTGCAGGTTGAAACAGATAGTAAGGTCATGGATAGGGGGAGAGTCGGGATTCTAT
176861_8776167_ConO_7831-8099_1_2	119	239	TGCACCTCCCATCAAGGGTGCCCTAAATTGCACCTCACAAATAACTGGAATGATTTTACAACTGGACTATCCATGGAACAACACAGAGGGAAAGGCCACAAATAACACAAAAAACAATAC
176861_8776168_ConO_8270-8389_1_1	1	120	GGGAATGCTATTCTTGGGGGTTCTAAGTGCAGCAGGTAGCACTATGGGCGCAGCGGCAACAGCGCTGACGGTACAGACCCACACTTTGATAAAGGGTATAGTGCAACAGCAGGACAACC
176861_8776169_ConO_8524-8658_1_1	1	120	GAAAGCTCATCTGCTACACATCAGTACAATGGAACACAACATGGACACCAGGTAGATGGAAAACAGAGGAGGGGATGGACGCAATCTGGGGCAACCTGACATGGCAGGAATGGGATCAG
176861_8776170_ConO_8524-8673_1_1	1	120	GAAACCTAATTTGCTACACATCAGTACAATGGAATAAGACATGGAGCCCTGGTACTAATGAAGAAAGCATTTGGGAGAACCTGACATGGCAGGAATGGGATCAACAGATAAGCAACATA
176861_8776171_ConO_8524-8661_1_1	1	120	GACAATTAGTCTGCTACACATCAGTAAAATGGAACACAACATGGGGAAGCAATATTCCTAATATTACTAATCTGGAGGAAATTTGGGGCAAACTAACCTGGCAGGAATGGGATCAGCAG
176861_8776172_ConO_8770-8889_1_1	1	120	ATTGGCTTGACATAACTAAATGGTTGTGGTATATAAAAATAGCAATAATCATAGTAGGAGCACTAATAGGTGTGAGAATTGTCATGATAGTACTTAATCTAGTGAGAAACATTAGGCAG
176861_8776173_ConO_9397-9522_1_1	1	120	GGGCATCAGTGAGAGAAAGAATGAGAAAAACTAGAACCATTAGAACTAGAACTTCCCCTGAGTCTGAACCAGCAGCACCTGGAGTAGGAGCAGTCTCCAGAGCATTAGCAGCTAGAGGA
176861_8776174_ConO_9397-9537_1_1	1	120	GGGCAGCAGTAAGAGAAAGAATGAGAAGAACTAGAACTTTCCCTGAGTCTGAACCATGCGCACCTGGAGTAGGACAGATCTCCAGGGAATTAGCAGCTAGAGGAGGGATACCAAGTTCC
176861_8776175_ConO_9397-9534_1_1	1	120	GGTCAGAAGTAAGAGAAAGAATAAGAAGAGCTCACAGTGATCACAGTGATCCTGAACCATGTGCACCTGGAGTAGGAGAACTCTCCAGGGAACTAGCAGCTAGAGGAGGAATACCAAGT
176861_8776176_ConO_9901-10021a_1_1	1	120	TCCAGCTTGTAATCATGGATTTGAGGATGCACACGGGGAGATACTAAAATGGCAGTTTGATAGATCATTAGGCAGCACCCATGTAGCTATGATAACCCACCCAGAGCTCTTCCACAAGG
176861_8776177_ConO_9901-10021b_1_1	1	120	TCCAGCTTGTAATCATGGTTTTGAGGATGAACATGGAGAAGTACTAAAGTGGCAGTTTGATAGATCATTAGGCTTAAATCATGTAGCCTTACAACTGCACCCAGAGCTCTTCCCCAAAG
176861_8776178_ConP_1_1	0	120	TGGATGGGTTAATTTATTCACCTGGGAGAGCAGAAATACTAGATCTCTGGATCTATCACACTCAGGGGTTCTTCCCTGACTGGCAGCATTACACTCCAGGGCCAGGAGAAAGATTCCCAC
176861_8776178_ConP_1_2	119	239	CTGACCTTTGGGTGGCTGTTTAAACTAGTACCAGTCTCTGAGGTAGAAGTTGAGGAAATGGGAGATGAGCAGGAGAAAGCTAAGCTGCTACATCCAGCCTGCACTTATGGGGATTCAGAT
176861_8776178_ConP_1_3	238	358	TCATCATAGGGAGATCTTAATGTGGAAGTTTGACAGATCACTGGGAGAAAGACATGTTGCCTTACAAAAGCACCCGGAACTGTTTACTAAAGACTAAAGTGCTGACGCTACGCAGCTACT
176861_8776178_ConP_1_4	357	477	TAAGTTGCTGACACTGCAGGGACTTTCCGGGGACGGAAAGTCCCGAGGGCGGAACAAGGGGAGGAGCAGGGGAGTGGTTTCACCCTCAGAGCTGCATATAAGCAGCTGCTTCACGCTTGT
176861_8776178_ConP_1_5	476	596	TACTGGGTCTCTGTGACAGACCAGATTAGAGCCTGGGAGCTCTCTGGCCTTAAGCAGAACCCACTGCTTGGAAGCTCAATAAAGCTTGCCTTGAGTGAGTAAAGCAGTGTGTGCTCATCT
176861_8776178_ConP_1_6	595	715	TGTTCAGAACTCTGGTGTCTAGAGATCCCTCAGAGCACTTCTAGCCGAGTGGAAAATCTCTAGCAGTGGCGCCCGAACAGGGACTTGAAAGTGAAACCAGTTTCTGAATCCTCCGACGCA
176861_8776178_ConP_1_7	714	834	ACGGGCTCGGCTCAGCGGAGTGCACCTGCTGAGAGGCGAGAGGAGCTCACAGCGGTGAGTACATTTTGTCAGTGGTGACTGACCCTAGAGGAAGAGGCGAAGTCTCTAGGGGAGGAGATG
176861_8776178_ConP_1_8	833	953	GGGTGCGAGAGCGTCAGTGTTGACAGGGGGCCGATTGGATGCATGGGAAAGAATTAGGCTTAGGCCAGGAAGTAAGAAAAAATATAAGCTAAAACATATAGTATGGGCAAGCAGAGAGCT
176861_8776178_ConP_1_9	952	1072	TGGAAAGATTTGCATGTAATCCTGGGCTTATGGAAACAGCAGAAGGCTGCGAAAAATTATTAGAGCAGTTAGAACCAGCTCTCAGAACAGGTTCTGATGGCCTGCAGTCTCTTTGGAACA
176861_8776178_ConP_1_10	1071	1191	ACCCTAGTAGTTTTATGGTGTGTTCACAAAAGAGTAGATATAAGTGACACACAGCAGGCCATTACAAAATGGAAGGAGGAAATGCAGAAAAGAAAGAAAGCATCAGAGGGCAGCACTGGA
176861_8776178_ConP_1_11	1190	1310	AACAAGTCAAAACTATCCTATTGTGCAGAATGCCCAGGGGCAAATGACCCATATGCCGCTGTCTCCCAGGACGCTGAATGCCTGGGTGAAGGCAGTAGAAGAAAAGGCCTTCAACCCTGA
176861_8776178_ConP_1_12	1309	1429	AAATTATCCCTATGTTTATGGCCTTGTCAGAAGGGGCTATACCTGATGACATCAATACTATGCTTAATGCAGTAGGCGGACATCAGGGAGCGTTGCAGGTGTTGAAAGAGGTAATTAATG
176861_8776178_ConP_1_13	1428	1548	GAAGAGGCTGCAGAATGGGATAGAACACATCCTGTTCCAGTAGGACCATTACCACCAGGGCAGCTTAGAGACCCAACAGGAGGGGATATTGCAGGAACCACTAGTACCAAGCAGGAACAG
176861_8776178_ConP_1_14	1547	1667	GATAACCTGGATAACAAGGAACAATCCTATACCAGTAGGAGACATCTATAGAAAGTGGATAGTGTTGGGGCTCAATAAAGTGGTAAAAATGTACAGCCCCATTAGCATTCTGGACATAAA
176861_8776178_ConP_1_15	1666	1786	AGCAGGGACCTAAGGAACCATTTAGAGATTATGTAGATAGATTCTACAAAACCCTCAGAGCAGAGCAAGCTAGTCAGGAAGTAAAGAGTTGGATGACAGACACCTTACTAATACAAAATG
176861_8776178_ConP_1_16	1785	1905	GCTAATCCAGATTGTAAGCAGATTTTGAAAGCTCTGGGACCAGGTGCCACCTTAGAAGAAATGATGAATGCCTGTCAAGGAGTAGGGGGACCAACACATAAGGCCAGGGTCTTGGCAGAA
176861_8776178_ConP_1_17	1904	2024	AGCTATGGCAGCAGCTAATCAAGCTAACCAAGAATTAAAAGGAGGGTATACAACAGTTTTCATGCAGAGTGGACAGAGAAAGCCAGTTAAGTGCTTTAACTGTGGAAAAGTAGGACACAT
176861_8776178_ConP_1_18	2023	2143	TAGCAAAGAACTGTAAGGCACCTAGGAGAAGAGGATGTTGGAAGTGTGGACAGGAAGGTCATCAAATGAAAGACTGCAAATCAGGAAGACAGGCAAATTTTTTAGGGAAGATCTGGCCTC
176861_8776178_ConP_1_19	2142	2262	CCTGGGGGCAAGAGGCCAGGCAACTATGTACAGAAACAAGTACAACCGACAGCCCCACCTATGGAGGAGGACGAGATGACTCGGAACAAGCAGAGGGAGGAAAAGGAGGACGAGAAAGAG
176861_8776178_ConP_1_20	2261	2381	GTACCCTTTAGCCTCCCTCAGATCACTCTTTGGGACAGACCAGTAATACCAGTAAGAATAGGGGGGCATATTTGTGAGGCTCTATTAGATACAGGAGCTGATGACACAGTAGTAGATAAC
176861_8776178_ConP_1_21	2380	2500	CTTACCTTTAGAAGGAAGATGGAAACCAAAAATGATAGGAGGAATAGGGGGTTTTATAAAAGTAAAAGAATTTGAAGAAGTTAAGGTAGAAATAGAAGGAAGAGAAGTTTATGGAACTGT
176861_8776178_ConP_1_22	2499	2619	TATTAGTGGGCCCAACCCCAGTAAATATAATAGGAAGAAATATTTTGACATTAATTGGATGTACTCTGAGTTTCCCCATCAGCTCCATAGAAATAGTACCAGTAAAATTAAAGGCAGGAA
176861_8776178_ConP_1_23	2618	2738	ATGGATGGGCCTAGGGTAAAGCAATGGCCCTTGTCAAAGGAAAAAATAGAAGCTTTAAGAGCCATTTGTCAAGAGATGGAACAGGAAGGAAAAATAACAAAAATTGGGCCTGAAAATCCA
176861_8776178_ConP_1_24	2737	2857	ATATAACACCCCCATCTTTGCAATAAAAAAGAAAGATGGCAGCAAGTGGAGGAAATTAGTAGACTTCAGAGAACTAAATAAGAGAACACAAGATTTCTGGGAAGTACAATTGGGAATCCC
176861_8776178_ConP_1_25	2856	2976	CTCATCCAGGAGGTCTTAAGAAAAGGAAATCAGTGACCGTATTAGATGTAGGGGATGCTTATTTCTCCTGTCCTTTAGATCCAGATTTTAGAAAATATACAGCTTTCACTATACCTAGTG
176861_8776178_ConP_1_26	2975	3095	GTAAATAATGAAACACCAGGAATTAGATATGAGTATAATGTTCTGCCTCAGGGATGGAAGGGATCACCAGCCATCTTCCAGCATTCAATGACTAAGATCTTAGAACCCTTTAGGGAGAGT
176861_8776178_ConP_1_27	3094	3214	TAATCCAGAAGTAGAAATCTATCAATACATGGATGATCTATATGTAGGGTCAGATTTGCCTTTGTCTGAACATAGACAGCGGGTAGAGAAACTTAGGGAACACCTCTATGTATGGGGTT

176861_8776178_ConP_1_28	3213	3333	TCACAACTCCTGACAAAAAACATCAAAAGGAGCCTCCTTTCCTCTGGATGGGATATGAGCTCCATCCTGACAAGTGGACGGTGCAACCCATCATATTACCAGAAAAGGAAGGTTGGACAG
176861_8776178_ConP_1_29	3332	3452	GTAAATGACATCCAGAAGCTAGTAGGAAAGTTAAATTGGGCAAGTCAAATTTATCCAGGAATAAGGGTAAAAGAGTTATGCAAACTAATTAGAGGAACTAAATCCTTAACAGAAGTAGTT
176861_8776178_ConP_1_30	3451	3571	TGCTCTTACTAGAGAAGCAGAATTAGAACTAGAGGAAAATAAAGAAATTTTAAAAGAACCAGTGCATGGAGTTTATTACCAACCAGAAAAGGAATTAATAGTAGACATACAGAAACAGGG
176861_8776178_ConP_1_31	3570	3690	GAGCGGGACAATGGACTTATCAGATATTCCAGGAAGAACATAAAAACTTGAAAACAGGGAAATATGCCAGGCAAAAGGCTACTCACACCAATGATATAAGGCAACTGGCAGAAGTAATAC
176861_8776178_ConP_1_32	3689	3809	CAGAAGGCATCACAAGAAAGCATAGTGATATGGGGAAAACTACCCAAGTTTAGACTACCAATAAATAGAAACGTGTGGGAGACTTGGTGGTCAGACTATTGGCAAGCCACCTGGATACCT
176861_8776178_ConP_1_33	3808	3928	TGAATGGGAATTTGTTAGCACACCCCCTCTTATTAAGCTCTGGTATCAGCTAGAAAAAGACCCCATACCAGGAATAGAAACCTTTTATGTAGATGGGGCAGCAAACAGAGAGACCAAGTT
176861_8776178_ConP_1_34	3927	4047	TAGGCAAAGCTGGATATGTAACAGATAGGGGTAGGCAGAAGATAATCAAACTAGAAGGAACAACTAATCAAAAAGCAGAATTAGAAGCAGTGTTGTTAGCCTTAAAAGAATCAGGAAAAC
176861_8776178_ConP_1_35	4046	4166	CAGGCTAACATAGTAACAGACTCCCAATATGTGTTAGGGATCATCTCAGCAACTCCAGATCAAAGTGACTCCCCCCTAGTGCAAAAAATAATAGAAGAAATGACAAAAAAGGAAAAGGTA
176861_8776178_ConP_1_36	4165	4285	ATACCTATCATGGGTACCAGCACACAAAGGCATAGGGGGTAATGAAAATATAGATAAATTAGTCAGTAAAGACATTAGAAGAGTGTTATTCCTGGAAGGCATAGACCAAGCACAGGAGGA
176861_8776178_ConP_1_37	4284	4404	ATCATGAAAAATATCACAGTAATTGGAGAGCCCTAGCTAGTGACTTTGGCCTTCCACCAATAGTGGCAAAAGAGATTATCAACAATTGCCCCAAATGTCATGTAAAAGGGGAAGCCATGC
176861_8776178_ConP_1_38	4403	4523	CATGGACAGGTAGACTGCAGTCCAGGAATTTGGCAACTGGACTGTACCCACACGGAAGGAAAAATTATCCTTGTGGCAGTCCATGTAGCCAGTGGCTTCATGGAAGCAGAAGTAATTCCA
176861_8776178_ConP_1_39	4522	4642	AGCAGAAACAGGACAAGAAGCTGCATATTTTGTGCTCAAATTGGCATCAAGATGGCCTGTAAAAGTAATACACACAGACAATGGGCCTAACTTTACTAGCGCTGCAGTAAAGGCAGCCTG
176861_8776178_ConP_1_40	4641	4761	GTTGGTGGCTTAATATAACTCATGAGTTTGGGATACCCTACAATCCCCAAAGTCAGGGAGTAGTGGAATCAATGAATAAAGAATTAAAGAAAATCATACATCAAGTACGAGATCAAGCTG
176861_8776178_ConP_1_41	4760	4880	GAGCACTTAAAGACAGCAGTTCAAATGGCAGTATTTGTTCACAATTTTAAAAGAAAAGGGGGGATTGGGGGGTACACTGCTGGAGACAGGATCATAGATATTCTGGCTACACAAATACAA
176861_8776178_ConP_1_42	4879	4999	AACAACAGAATTACAAAAACAAATTTTAAAAATTCAAAATTTTCGGGTCTATTACAGAGACAGCAGAGACCCTATTTGGAAAGGACCAGCGACGCTCCTGTGGAAAGGTGAAGGGGCAGT
176861_8776178_ConP_1_43	4998	5118	TAGTCATACAAGACAAAGGAGATATTAAAGTAGTCCCTAGGAGAAAAGCAAAAATAATTAGAGATTATGGAAAACAGATGGCAGGTGATGATTGTGTGGCAGATACCCAGAGAGAAAGTG
176861_8776178_ConP_1_44	5117	5237	GAAAGCCTGGAACAGTCTGGTCAAATATCACAAATATAGGTCTAAAAAGACAAGGAATTGGTTTTATAGGCATCATTATGAGACAATCCATCCTAGGATTAGTTCAGGAGTTTACATTCC
176861_8776178_ConP_1_45	5236	5356	CAGTAGGAACAGCAACCATTATTGTGACTACTTATTGGGGGCTTATGCCTGGGGAAAGAGAAGAACAATTAGGACATGGAGCAAGTGTGGAGTGGAGACAAGGTAAATACACCACACAGA
176861_8776178_ConP_1_46	5355	5475	ATAGATCCAGAAACAGCAGATAGGCTAATTCATCTCCACTACTTTCAATGTTTTTCAGATTCGGCTGTGAGGAGGGCAATACTAGGGGACAGGGTATTGAACAAGTGTGAATACTCAGCA
176861_8776178_ConP_1_47	5474	5594	AGGACATAGTCAGGTAGGCTCCTTGCAGTATTTAGCCTTAAAAGTGGTAGTAGGGAAAGTAAAAAGGAAGCCACCCCTCCCTAGTGTCCAGATATTGACACAAGACATATGGAGCAAGCC
176861_8776178_ConP_1_48	5593	5713	CCCAGAGGACCAAGGGCCGCCAAGAGAGCCATTCAATGAGTGGATGCTACAAACCTTAGAGGAACTCAAGGAGGAAGCAGTAAGACACTTCCCTATGCATTGCTTACACTCATTAGGACA
176861_8776178_ConP_1_49	5712	5832	AATACATCTATAATACCTATGGGGACACCTGGGAGGGAGTAACTGCAATTATTAGGATCCTACAACAATTAATCTTTATCCATTATAGAATTGGATGCCACCATAGTAGAATAGGTATCT
176861_8776178_ConP_1_50	5831	5951	TTGCCACCCTCTCGAAGAGGAAGGAGACATGGACCCAGTAGATCCTGACCTGCCTCCATGGCAACAGCCAGGGAGTCAGCCCTCAAGCCCATGTAACAATTGCTACTGCAAAGCCTGCTG
176861_8776178_ConP_1_51	5950	6070	GCTATCATTGCTATGTTTGTTTCACAAAGAAGGGTTTGGGAATCTCCTATGGCAGGAAGAAGCGACGAAGACCTGCGAGAGCTGATAACAACAATCAGAATCATCAAGATCCTGTATCAA
176861_8776178_ConP_1_52	6069	6189	AAGTAGTAAGTAGAAATAATAAGTTGTATTAATGCATCCTAGGGATGAGGCAGTATTAATAATAGCTGGGGTATTATTAATTTGCAGTATAGTAGTTTGGGGGAAGGTCCTGCTATTAGT
176861_8776178_ConP_1_53	6188	6308	TGCTAAAAGAGAGAGAAAGGGATAAGTTTGTACAAAGGCTAGCAAGGTGGAGAGAAAGGCAAGAAGATGAGGGGTATGAGAGTAATGAAGAAGAAGAAGAACAGCTTAGGGAACTTGGGA
176861_8776178_ConP_1_54	6307	6427	AATCTGCTTGGCTTTGGTCATATACTTTAATGCTATTAGCTATGCTAGTGGAACACGCTATGTTACTGTATATTATGGGGTTCCAGTTTGGAGAGATGCTAAAGTTACCTTATTTTGTGC
176861_8776178_ConP_1_55	6426	6546	CAGCAGATGCATCGCTAACTAGCAAAGAGCAGCATAATATTTGGGCCACCCAGGCCTGTGTGCCCACAGACCCTACACCAATAGAGGTCCGGATAAATGTAACAGAGTCTTTTAATATTT
176861_8776178_ConP_1_56	6545	6665	TGGGAAAATTATATGGTGACACAAATGCAGGAAGACATCATTAGCTTATGGGATCAGAGCCTTAAACCTTGTGTAAAATTGACAGTCCTATGTGTTACTATGAATTGTAGTGAATGCCAC
176861_8776178_ConP_1_57	6664	6784	CAAGGAGGCTAACTGTAATAATAACAGTAGCTCCTCTGAGAATAGCACCGCCGCCAATCCATTAGATCTATTTAAGTGCAGTTTTAATACAACCACAGTATTGAAAGATAAAAAGCAGGA
176861_8776178_ConP_1_58	6783	6903	AACAGCAAGCACTCTTCTATAGAACAGACCTAATAGCACTGAATGAGACAGCAAATAATACACTATATAGATTAATTAATTGCAACACCACTACCATTACACAAGCATGCCCAAAAGTGA
176861_8776178_ConP_1_59	6902	7022	ACTTTTGAGCCACTACCCATACAGTATTGTGCACCAGCAGGGTATGCACTAATGAAATGCAATCAGAAAGGATTTAATGGTACAGGACCTTGTAATCAGACAGTCATAACACACTGTACA
176861_8776178_ConP_1_60	7021	7141	ACATGGAATTAAGCCAACAGTGTCAACACAATTGATACTCAATGGAACTCTAGCAGAGGGAGAGCCCTTAGTAATTACTCAAAATGTGTCAGATACAAGAAATGTCATCATAGTAAAATT
176861_8776178_ConP_1_61	7140	7260	TAAATGAGAATGTGTCACTCACCTGTGTAAGACCAGGTAATAACACAAGAGGACAAGTGCAACTAGGAGCCATGACATGGTATAATATGAAACTCTACGCAGGTGATATCAGGGAAGCTC
176861_8776178_ConP_1_62	7259	7379	CATTGTAACGTTTCTATGGAAAATTGGACAGGAACCTTAGAATGTGTCAGTGAGGCCATCTGGAAGGCTTATCCTCAACCTCCCAACCATAACCATACATTTGTCTTTAGAAACAGTACA
176861_8776178_ConP_1_63	7378	7498	AGGGGGAGACCCAGAGGTTTCTTCTCTGCACTTCAGTTGTCATGGGGAATTCTTTTATTGTAACACCAGCAGCCTGTTTAATTATAGCTATACTTGTAATGAGAAGGGTGTATGCAGTAT
176861_8776178_ConP_1_64	7497	7617	TCAATAATCATACAGGTAATCACACAGGTGAGAACATAATAACACTGCCTTGTAGACTAAAGCAAGTAGTTAATTCATGGATGAGAGTGGGATCAGGCTTGTTTGCGCCACCTATCGAGG
176861_8776178_ConP_1_65	7616	7736	GGACAATTGCAGTGCCACTCCAACATTACAGGTTTAATATTAGACAGAGCATCACCTTATAATGCCAACAGCAGCAGCAACACCACTTTAAGCCCTACAGGGGGAGAAATGAGACATATC
176861_8776178_ConP_1_66	7735	7855	CTGGAGAAGTGAGTTATATCCCTACAAGGTAGTTCAAGTAAAAGCTCTGGCTGTGGCACCTACAAGAGTCTCCAGACCTACAATCATGGCTCATGATGCTCATAGGAAAAAAAGGGGAGC
176861_8776178_ConP_1_67	7854	7974	CAGGCCTAGGAATGCTATTCCTTGGGTTTCTGAGTGCAGCAGGAAGCACTATGGGCGCAGCGGGAGTCACGCTGACGGTACAGGCCAGGCAATTATTGCATGGTATAGTGCAGCAGCAGA
176861_8776178_ConP_1_68	7973	8093	AACAATATGCTAAGAGCTATAGAGGCACAGCAAGAATTGCTGAGACTCTCTGTGTGGGGCATAAGACAGCTCCGAGCTCGCCTGCTTGCCATTGAAACCTACTTAAGGGATCAGCAACTC
176861_8776178_ConP_1_69	8092	8212	CCTAGGACTGTGGGGATGCTCAGGACAAATAGTATGTTATACTAATGTGCCATGGAATAGGACCTGGACCAACAAAAGTGAAACTGAATTAGATGGCATTTGGAGTAATCTAACATGGCA
176861_8776178_ConP_1_70	8211	8331	AGGAATGGGACAAACTGGTGGATAATTACACTGACACAATTTATTTGGAAATACAAAGAGCACAAGAGCAACAGAAGGCAAATGAAAGAAAGTTATTAGAATTAGACCAATGGGCACAAT
176861_8776178_ConP_1_71	8330	8450	TTGTGGAGCTGGTTGGACATAACACAATGGTTGTGGTACATAAAAATTTTCATTATGATAGTAGGAGGAATTATAGGACTAAGAATCTTATTGGCTATAGTTAATGTAGTCAGGAGAGTC
176861_8776178_ConP_1_72	8449	8569	CAGGCAGGGATACTCACCTGTATCGTTGCAGACCCTTGGCCTGCACGGGGACCCAGCAGGAATAGCACCAGGAACAGACGAAGAAGGTGGAGAAGCAGGCAACGGCAGATCGATCAGATT
176861_8776178_ConP_1_73	8568	8688	TGCTGGACGGATTATTGCCTCTCGTCTGGGACGACCTGAGGAACCTGGTGGTGCAGATCTACCAGATATTAGTCGGCTGCATATTGAGGATCAAGGACCTCCTAACAATTCTGTGGATAC
176861_8776178_ConP_1_74	8687	8807	CACCTGGGCCAACTGCTGAACAGAGGGCTTAACTGCCTGCGAGACTGCTTTGCTGCGTGTGGCTATTGGACTCAAGAATTAAAACAAAGTGCAACCAGCTTGTTGGACACTGTTGCTATA
176861_8776178_ConP_1_75	8806	8926	ATCAGTTGCAGGGTGGACTGATCAAGTAATAGCAGTAGGGCAACAAATAGGAAGAGGCTTCTTGAACATACCAAGAAGGTTAAGACAAGGGATAGAAAGAAGCTTACTATAATGGGGAAT
176861_8776178_ConP_1_76	8925	9045	TGCATGGAAGAAAAGTAGCTTAGTGGGCTGGCCAGCAGTCAGGGAAAGAATAAATCAGACTACCCCAGTAACACCTGCATCCGGGGTTGGGGAGATTTCCAAAGAATTAGCACAAGGAAA
176861_8776178_ConP_1_77	9044	9164	AAGGAATACCCAGTAAATTTAATTCAAAGAACAATGCAGCATTAGCCTTCTTGGAAGCTCATGAGGACGAAGAAGTAGGATTCCCAGTTAGGCCTCAAGTACCCTTAAGACCCATGACAT
176861_8776178_ConP_1_78	9163	9283	TACAAGGCAGCATTTGACCTCAGCTTCTTTTTAAAAGAAAAGGGAGGACTGGATGGGTTAATTTATTCACCTGAGAGAGCAGAAATCCTAGATCTCTGGATCTATCACACTCAGGGATTC
176861_8776178_ConP_1_79	9282	9402	CTTCCCTGACTGGCAGCATTACACTCCAGGGCCAGGAGAAAGATACCCCCTGACCTTTGGGTGGCTGTTTAAACTAGTACCAGTCTCTGAGGTAGAAGCTGAGGAAATGGGAGATGAGCA

176861_8776178_ConP_1_80	9401	9521	AGGAGAAAGCTAAGCTGCTACATCCAGCCTGCACTTATGGGTTTTCAGATCATCATAAGGAGATCTTAGTGTGGAAGTTTGACAGATCACTTGGAGGAAAACATGTTGCCTTACAAAAGC
176861_8776178_ConP_1_81	9520	9640	CACCCGGAACTGTTTATTAAAGACTAAAGTGCTGACGCCACGCAGCTGCTAAAGTTGCTGACACTGCAGGGACTTTCCGGGGACGGAAAGTCCCGAGGGCGGAACAAGGGGAGGAGCAGG
176861_8776178_ConP_1_82	9639	9759	GGGAGTGGTTTCACCCTCAGAGCTGCATATAAGCAGCTGCTTCACGCTTGTACTGGGTCTCTGTGACAGACCAGATTAGAGCCTGGGAGCTCTCTGGTCTTAAGCAGAACCCACTGCTTG
176861_8776178_ConP_1_83	9758	9873	GGAAGCTCAATAAAGCTTGCCTTGAGTGAGTAAAGCAGTGTGTGCTCATCTGTTCAGAACTCTGGTGTCTAGAGATCCCTCAGAGCACTTCTAGCCGAGTGGAAAATCTCTAGCA
176861_8776179_P-6671-7034_1_1	0	120	AGCGACTGCAGCACGGTTGACTGTACTAATAACTCCTCCAGGGTTAATAACAGCACCGACAGCACCAACACCAGCAAAACCAATCCATTAGAATTATTTCAGTGCAGTTTTAATACAACC
176861_8776179_P-6671-7034_1_2	119	239	CACAATAGTAAAAGATAAACAGCAGACACAGCAAGCACTCTTTTATAGAGCAGACCTAACAAAATTGGATAATGACAATAGTACATATAGATTAATTAATTGCAACACCACTACCATTAC
176861_8776179_P-6671-7034_1_3	238	358	CACAGGCATGCCCAAAAGTGAACTTTGAGCCGCTACCCATACAGTATTGTGCACCAGCAGGGTATGCACTAATGAAATGCAATCAGACAGGATTTAATGGTACAGGACCTTGTAATAAGA
176861_8776180_P-7498-7874_1_1	0	120	GTTTAACTTTAGTTATACTTGGAATGACACCACCTGGCGGGCTGCAGGCAATCATAATACAAGTGAAAATATAACACTGTCTTGTAGACTGAAGCAAGTAGTTAATTCATGGATGAGAGT
176861_8776180_P-7498-7874_1_2	119	239	TGGGATCAGGCTTGTTTGCGACACCTATTGAGGGGGAATTAAAGTGTCAATCCAACATTACAGGTTTAATGTTAGAAAGAGAACTACCTTACAATGACAGCAGCAAGAACACCACTTTAA
176861_8776180_P-7498-7874_1_3	238	358	AGTCCTATAGGGGGAGACATGACAAATATCTGGAGAAGTGAGTTATATCCCTACAAAGTAGTTCAAGTAAAAGCTCTGGCCGTGGCACCTACAAAAATTTCCAGACCTACAATTATGGCT
176861_8776181_HIV2-Con2A_1_1	0	120	TGGAAGGGATGTTTTACAGTGAGAGAAGACATAGAATCCTAGACATATACTTAGAAAAGGAAGAAGGGATAATTCCAGATTGGCAGAATTATACTCATGGGCCAGGAGTAAGATACCCAA
176861_8776181_HIV2-Con2A_1_2	119	239	ATGTTCTTTGGGTGGCTGTGGAAGCTAGTACCAGTAGACGTCCCACAAGAGGGAGAGGACGATGAGACCCACTGCTTAATACACCCAGCACAAACAAGCAGGTATGATGACCCGCATGGG
176861_8776181_HIV2-Con2A_1_3	238	358	GGAAACACTAGTGTGGAGGTTTGACCCCATGCTGGCCTATGAGTACAAGGCCTTTAATCTATACCCAGAGGAATTTGGGCACAAGTCAGGCTTGCCAGAAGAAGAGTGGAAGGCAAAACT
176861_8776181_HIV2-Con2A_1_4	357	477	TGAAAGCAAGAGGGATACCATTTAGTTAAAGACAGGAACAGCTATACTTGGTCAGGGCAGGAAGTAGCTACTGAAAACAGCTGAGACTGCAGGGACTTTCCAGAAGGGGCTGTAACCAGG
176861_8776181_HIV2-Con2A_1_5	476	596	GGGAGGGACATGGGAGGAGCTGGTGGGGAACGCCCTCATACTTTCTGTATAAATGTACCCGCTGCTTGCATTGTACTTCAGTCGCTCTGCGGAGAGGCTGGCAGATTGAGCCCTGGGAGG
176861_8776181_HIV2-Con2A_1_6	595	715	GTTCTCTCCAGCACTAGCAGGTAGAGCCTGGGTGTTCCCTGCTAGACTCTCACCAGTGCTTGGCCGGCACTGGGCAGACGGCTCCACGCTTGCTTGCTTAAAGACCTCTTAATAAAGCTG
176861_8776181_HIV2-Con2A_1_7	714	834	GCCAGTTAGAAGCAAGTTAAGTGTGTGTTCCCATCTCTCCTAGTCGCCGCCTGGTCATTCGGTGTTCACCTGAGTAACAAGACCCTGGTCTGTTAGGACCCTTCTTGCTTTGGGAAACCG
176861_8776181_HIV2-Con2A_1_8	833	953	GAGGCAGGAAAATCCCTAGCAGGTTGGCGCCCGAACAGGGACTTGAAGAAGACTGAGAAGCCTTGGAACACGGCTGAGTGAAGGCAGTAAGGGCGGCAGGAACAAACCACGACGGAGTGC
176861_8776181_HIV2-Con2A_1_9	952	1072	CTCCTAGAAAGGCGCGGGCCGAGGTACCAAAGGCGGCGTGTGGAGCGGGAGTGAAAGAGGCCTCCGGGTGAAGGTAAGTACCTACACCAAAAACTGTAGCCAGAAAGGGCTTGTTATCCT
176861_8776181_HIV2-Con2A_1_10	1071	1191	TACCTTTAGACAGGTAGAAGATTGTGGGAGATGGGCGCGAGAAACTCCGTCTTGAGAGGGAAAAAAGCAGATGAATTAGAAAAAATTAGGTTACGGCCCGGCGGAAAGAAAAAATACAGG
176861_8776181_HIV2-Con2A_1_11	1190	1310	GTTAAAACATATTGTGTGGGCAGCGAATGAATTGGACAGATTCGGATTAGCAGAGAGCCTGTTGGAGTCAAAAGAAGGTTGCCAAAAAATTCTTACAGTTTTAGATCCATTAGTACCGAC
176861_8776181_HIV2-Con2A_1_12	1309	1429	CAGGGTCAGAAAATTTAAAAAGCCTTTTTAATACTGTCTGCGTCATTTGGTGCATACACGCAGAAGAGAAAGTGAAAGATACTGAAGAAGCAAAAAAAATAGTACAGAGACATCTAGTGG
176861_8776181_HIV2-Con2A_1_13	1428	1548	GCAGAAACAGGAACTGCAGAGAAAATGCCAAATACAAGTAGACCAACAGCACCACCTAGCGGGAAAGGAGGAAACTTCCCCGTACAACAAGTAGGCGGCAACTATACCCATGTGCCGCTG
176861_8776181_HIV2-Con2A_1_14	1547	1667	GAGTCCCCGAACCCTAAATGCTTGGGTAAAATTAGTAGAGGAAAAGAAGTTCGGGGCAGAAGTAGTGCCAGGATTTCAGGCACTCTCAGAAGGCTGCACGCCCTATGATATTAATCAAAT
176861_8776181_HIV2-Con2A_1_15	1666	1786	TGCTTAATTGTGTGGGCGACCATCAAGCAGCTATGCAAATAATCAGGGAGATTATTAATGAAGAAGCAGCAGATTGGGATGCGCAACATCCAATACCAGGCCCCTTACCAGCGGGGCAGC
176861_8776181_HIV2-Con2A_1_16	1785	1905	CTTAGAGAACCAAGAGGGTCTGACATAGCAGGGACAACAAGCACAGTAGAAGAACAGATCCAGTGGATGTTTAGGCCACAAAATCCTGTACCAGTAGGAAACATCTATAGAAGATGGATC
176861_8776181_HIV2-Con2A_1_17	1904	2024	CCAGATAGGGCTGCAGAAGTGTGTCAGGATGTACAACCCAACCAACATCCTAGACATAAAACAGGGACCAAAGGAGCCGTTCCAAAGCTATGTAGATAGATTCTACAAAAGCTTGAGGGC
176861_8776181_HIV2-Con2A_1_18	2023	2143	CAGAACAAACAGATCCAGCAGTAAAGAATTGGATGACCCAAACACTGCTAGTACAGAATGCCAACCCAGACTGTAAATTAGTACTAAAAGGACTAGGGATGAATCCTACCTTAGAAGAGA
176861_8776181_HIV2-Con2A_1_19	2142	2262	ATGCTAACCGCCTGTCAGGGGGTAGGTGGACCAGGCCAGAAAGCCAGATTAATGGCAGAAGCCTTAAAGGAGGCCATGGCACCAGCCCCTATCCCATTTGCAGCAGCCCAGCAGAGAAGG
176861_8776181_HIV2-Con2A_1_20	2261	2381	GACAATTAAGTGCTGGAATTGTGGAAAGGAAGGACACTCGGCAAGACAATGCCGAGCACCTAGAAGACAGGGCTGCTGGAAATGTGGCAAGCCAGGACATATCATGGCAAACTGCCCAGA
176861_8776181_HIV2-Con2A_1_21	2380	2500	AAAGACAGGCGGGTTTTTTAGGGATGGGCCCATGGGGAAAGAAGCCCCGCAACTTCCCCGTGGCCCAAGTTCCGCAGGGGCTAACACCAACAGCACCCCCAGTAGATCCAGCAGTGGACC
176861_8776181_HIV2-Con2A_1_22	2499	2619	CTACTGGAGAAATATATGCAGCAAGGGAGAAAGCAGAGGGAGCAGAGAGAGAGACCATACAAAGAGGTGACAGAGGACTTACTGCACCTCGAGCAGGGAGAGACACCATGCAGGGAGACG
176861_8776181_HIV2-Con2A_1_23	2618	2738	GACAGAGGACTTGCTGCACCTCAATTCTCTCTTTGGAAAAGACCAGTAGTCACAGCATACATTGAGGGTCAGCCAGTAGAAGTTTTACTAGACACAGGGGCTGACGACTCAATAGTAGCA
176861_8776181_HIV2-Con2A_1_24	2737	2857	AGGAATAGAGTTAGGGAGCAATTATAGTCCAAAAATAGTAGGGGGAATAGGGGGATTCATAAATACCAAAGAATATAAAAATGTAGAAATAGAAGTACTAAATAAAAGGGTAAGAGCCAC
176861_8776181_HIV2-Con2A_1_25	2856	2976	CCATAATGACAGGTGATACCCCAATCAACATTTTTGGCAGAAATATTCTGACAGCCTTAGGCATGTCATTAAATCTACCAGTCGCCAAGATAGAACCAATAAAAGTAATGCTGAAGCCAG
176861_8776181_HIV2-Con2A_1_26	2975	3095	GGAAAAGATGGACCAAAACTGAGACAATGGCCCTTAACAAAAGAAAAAATAGAAGCACTAAAAGAAATCTGTGAAAAAATGGAAAAAGAGGGCCAGCTAGAGGAAGCACCTCCAACTAAT
176861_8776181_HIV2-Con2A_1_27	3094	3214	TCCTTATAATACCCCCACATTTGCAATCAAGAAAAAGGACAAAAACAAATGGAGGATGCTAATAGATTTTAGAGAACTAAACAAGGTAACTCAAGATTTCACAGAAATTCAGTTAGGAAT
176861_8776181_HIV2-Con2A_1_28	3213	3333	TTCCACACCCAGCAGGATTAGCCAAGAAGAGAAGAATTACTGTACTAGATGTAGGGGATGCTTACTTTTCCATACCACTACATGAAGATTTTAGACAGTATACTGCATTTACTCTACCAT
176861_8776181_HIV2-Con2A_1_29	3332	3452	TCAGTAAACAATGCAGAACCAGGAAAAAGATATATATATAAAGTCTTGCCACAGGGATGGAAGGGATCACCAGCAATTTTTCAATACACAATGAGGCAGGTCTTAGAACCATTCAGAAAA
176861_8776181_HIV2-Con2A_1_30	3451	3571	AGCAAACCCAGATGTCATTATCATTCAGTACATGGATGATATCTTAATAGCTAGTGACAGGACAGATTTAGAACATGACAAAGTAGTCCTGCAGCTAAAGGAACTTCTAAATGGCCTAGG
176861_8776181_HIV2-Con2A_1_31	3570	3690	GATTTTCTACCCCAGATGAGAAGTTCCAAAAAGACCCTCCATACCAATGGATGGGCTATGAACTGTGGCCAACTAAATGGAAGCTGCAGAAAATACAATTGCCCCAAAAAGAAGTATGGA
176861_8776181_HIV2-Con2A_1_32	3689	3809	ACAGTCAATGACATCCAAAAGCTAGTGGGTGTCCTAAATTGGGCAGCACAAATCTACCCAGGGATAAAGACCAAACACTTATGTAGGCTAATTAGAGGAAAAATGACACTCACAGAAGAA
176861_8776181_HIV2-Con2A_1_33	3808	3928	AGTACAGTGGACAGAATTAGCAGAAGCAGAGCTAGAAGAAAACAAAATTATCTTAAGCCAGGAACAAGAGGGACACTATTACCAAGAAGAAAAAGAATTAGAAGCAACAGTCCAAAAAGA
176861_8776181_HIV2-Con2A_1_34	3927	4047	ATCAAGACAATCAGTGGACATATAAAATACACCAGGGAGAAAAAATTCTAAAAGTAGGAAAATATGCAAAGATAAAAAATACCCATACCAATGGGGTCAGATTGTTAGCACAGGTAGTTC
176861_8776181_HIV2-Con2A_1_35	4046	4166	CAGAAAATAGGAAAAGAAGCACTAGTCATTTGGGGACGAATACCAAAATTTCACCTACCAGTAGAGAGAGAAACCTGGGAGCAGTGGTGGGATAACTACTGGCAAGTGACATGGATCCCA
176861_8776181_HIV2-Con2A_1_36	4165	4285	AGACTGGGACTTCGTATCTACCCCACCACTGGTCAGGTTAGCATTTAACCTAGTAAAAGATCCTATACCAGGCGCAGAGACCTTCTACACAGATGGATCCTGCAATAGGCAGTCAAAAGA
176861_8776181_HIV2-Con2A_1_37	4284	4404	AAGGAAAAGCAGGATATGTAACAGATAGAGGGAGAGACAAGGTAAAGGTATTAGAGCAAACTACCAATCAGCAAGCAGAATTAGAAGCCTTTGCAATGGCACTAACAGACTCAGGTCCAA
176861_8776181_HIV2-Con2A_1_38	4403	4523	AAAGTCAATATTATAGTAGACTCACAGTATGTAATGGGAATAGTAGCAGGCCAACCAACAGAGTCAGAAAGTAGAATAGTAAATCAAATCATAGAAGAAATGATAAAAAAGGAAGCAATC
176861_8776181_HIV2-Con2A_1_39	4522	4642	CTATGTTGCATGGGTCCCAGCCCATAAAGGCATAGGAGGAAATCAGGAAGTAGATCATTTAGTAAGTCAGGGCATCAGACAAGTATTGTTCCTAGAAAAAATAGAGCCCGCTCAGGAAGA
176861_8776181_HIV2-Con2A_1_40	4641	4761	AACATGAAAAATATCATAGCAATGTAAAAGAACTATCCCATAAATTTGGAATACCCAAACTAGTGGCAAGACAAATAGTAAACACATGTGCCCAATGTCAACAGAAAGGGGAGGCTATAC
176861_8776181_HIV2-Con2A_1_41	4760	4880	CATGGGCAAGTAAATGCAGAATTAGGCACTTGGCAAATGGACTGCACACACTTAGAAGGAAAAATCATTATAGTAGCAGTACATGTTGCAAGTGGATTTATAGAAGCAGAAGTCATCCCA
176861_8776181_HIV2-Con2A_1_42	4879	4999	ACAGGAATCAGGAAGGCAAACAGCACTCTTCCTATTAAAACTGGCCAGTAGGTGGCCAATAACACACTTGCACACAGATAATGGTGCCAACTTCACTTCACAGGAAGTGAAGATGGTAGC

176861_8776181_HIV2-Con2A_1_43	4998	5118	CATGGTGGGTAGGTATAGAACAATCCTTTGGAGTACCTTACAATCCACAAAGCCAAGGAGTAGTAGAAGCAATGAATCACCACCTAAAAAATCAGATAAGTAGAATTAGAGAGCAGGCAA
176861_8776181_HIV2-Con2A_1_44	5117	5237	AATACAGTAGAAACAATAGTACTAATGGCAGTTCATTGCATGAATTTTAAAAGAAGGGGAGGAATAGGGGATATGACCCCAGCAGAAAGACTAATCAATATGATCACCACAGAACAAGAA
176861_8776181_HIV2-Con2A_1_45	5236	5356	AATACAATTCCTCCAAGCAAAAAATTCAAAATTAAAAAATTTTCGGGTCTATTTCAGAGAAGGCAGAGATCAGCTGTGGAAAGGACCTGGGGAACTACTGTGGAAGGGAGACGGAGCAGT
176861_8776181_HIV2-Con2A_1_46	5355	5475	TCATAGTCAAGGTAGGGACAGACATAAAAGTAGTACCAAGAAGGAAAGCCAAGATCATCAGAGACTATGGAGGAAGGCAAGAGCTGGATAGTGGTTCCCACTTGGAGGGTGCCAGGGAGG
176861_8776181_HIV2-Con2A_1_47	5474	5594	GATGGAGAAGTGGCATAGCCTTGTCAAGTATCTAAAATACAGAACAAAAGATCTAGAAAAGGTGTGCTATGTTCCCCACCATAAGGTGGGATGGGCATGGTGGACTTGCAGCAGGGTAAT
176861_8776181_HIV2-Con2A_1_48	5593	5713	TATTCCCATTAAAAGGAAAAAGTCATCTAGAGATACAGGCATATTGGAACCTAACACCAGAAAAAGGATGGCTCTCCTCTTATGCAGTAAGAATAACTTGGTATACAGAAAAGTTCTGGA
176861_8776181_HIV2-Con2A_1_49	5712	5832	ACAGATGTTACCCCAGACTGTGCAGACTCCCTAATACATAGCACTTATTTCTCTTGCTTTACGGCAGGTGAAGTAAGAAGAGCCATCAGAGGGGAAAAGTTATTGTCCTGCTGCAATTAT
176861_8776181_HIV2-Con2A_1_50	5831	5951	TCCCCAAGCCCATAAATCCCAGGTACCGTCACTTCAATTTCTGGCCTTAGTGGTAGTGCAACAAAATGACAGACCCCAGAGAAACAGTACCACCAGGAAACAGTGGCGAAGAGACTATCG
176861_8776181_HIV2-Con2A_1_51	5950	6070	GGAGAGGCCTTCGCGTGGCTAGACAGGACAGTAGAAGCCATAAACAGAGAGGCAGTGAATCACCTGCCCCGGGAGCTTATTTTCCAGGTGTGGCAAAGGTCCTGGAGATACTGGCATGAT
176861_8776181_HIV2-Con2A_1_52	6069	6189	TGAACAAGGGATGTCACCAAGTTACACAAAGTATAGATATTTGTGCTTAATGCAGAAAGCTATGTTCATACATTTTAAGAGAGGGTGCACTTGCCTGGGGGGAGGACATGGGCCAGGAGG
176861_8776181_HIV2-Con2A_1_53	6188	6308	GATGGAGACCAGGACCTCCCCCTCCTCCCCCTCCAGGTCTAGTCTAATGACTGAAGCACCAACAGAGTTTCCCCCGGAGGATGGGACCCCACCGAGGGAGCCAGGGGATGAGTGGGTAAT
176861_8776181_HIV2-Con2A_1_54	6307	6427	TAGAAATCCTGAGAGAAATAAAAGAAGAAGCTTTAAAGCATTTTGACCCTCGCTTGCTAATTGCTCTTGGCAACTATATCTATACTAGACATGGAGACACCCTTGAAGGCGCCAGAGAGC
176861_8776181_HIV2-Con2A_1_55	6426	6546	CTCATTAGAGTCCTACAACGAGCCCTCTTCATGCACTTCAGAGCAGGATGTGGCCACTCAAGAATTGGCCAAACAAGGGGAGGAAATCCTCTCTCAGCTATACCGACCCCTAGAGGCATG
176861_8776181_HIV2-Con2A_1_56	6545	6665	GCAATAACACATGCTATTGTAAGCAATGCTGCTACCATTGCCAGCTGTGTTTTTTAAACAAGGGGCTCGGGATATGTTATGACCGAAAGGGCAGACGAAGAAGGACTCCAAAGAAAACTA
176861_8776181_HIV2-Con2A_1_57	6664	6784	AAGGCTCATTCGTCTTCTGCATCAGACAAGTGAGTATGATGTGTGGTAGGAATCAGCTGCTTGTTGCCATTTTACTAGCTAGTGCTTGCTTAATATATTGCAACCAATATGTGACTGTTT
176861_8776181_HIV2-Con2A_1_58	6783	6903	TTCTATGGCATACCCGCGTGGAGAAATGCATCCATTCCCCTCTTTTGTGCAACCAAAAATAGAGATACTTGGGGAACCATACAGTGCTTGCCAGACAATGATGATTATCAGGAAATAGCT
176861_8776181_HIV2-Con2A_1_59	6902	7022	TTTAAATGTGACAGAGGCTTTTGATGCATGGGATAATACAGTAACAGAACAAGCAATAGAAGATGTCTGGAATCTATTTGAGACATCAATAAAACCATGTGTCAAATTAACACCTTTATG
176861_8776181_HIV2-Con2A_1_60	7021	7141	GTGTAGCAATGAACTGTACAAGGAACATGACCACATGGACAGGGAGGACAGACACCCAAAATATCACAACAATTATAAATGACACTTCTCCATGCATACGCGCAGACAACTGCTCAGGAT
176861_8776181_HIV2-Con2A_1_61	7140	7260	TTAGGGGAGGAAGAGATGGTCAATTGTCAGTTCAATATGACAGGATTAGAGAGAGATAAGAAAAAACAGTATAATGAAACATGGTACTCAAAAGATGTGGTTTGTGAAACAAATAATACC
176861_8776181_HIV2-Con2A_1_62	7259	7379	CACAACAAAAAAGAGCAAATGTTACATGAACCATTGCAACACATCAGTCATCACAGAGTCATGTGATAAGCACTATTGGGATGCTATGAGGTTTAGATACTGTGCACCACCGGGTTATGC
176861_8776181_HIV2-Con2A_1_63	7378	7498	CCCTGCTAAGATGCAATGATACCAATTATTCAGGCTTTGAGCCCAATTGCTCTAAAGTAGTAGCTGCTACATGCACAAGGATGATGGAAACGCAAACTTCTACTTGGTTTGGCTTTAATG
176861_8776181_HIV2-Con2A_1_64	7497	7617	GGCACTAGAGCAGAAAATAGAACATATATCTATTGGCATGGTAGAGATAATAGAACTATCATCAGCTTAAACAAATATTATAATCTCACTATGCATTGTAAGAGGCCAGGAAATAAGACA
176861_8776181_HIV2-Con2A_1_65	7616	7736	AGTTGTACCAATAACACTTATGTCAGGGTTAATGTTTCACTCCCAGCCAGTCATCAATAAAGACCCAGGCAAGCATGGTGCTGGTTCAAAGGCAAATGGAAGGAAGCCATGCAGGAGGT
176861_8776181_HIV2-Con2A_1_66	7735	7855	TGAAGCAAACCCTTGCAAAACATCCCAGGTATAAAGGAACCAATGACACAAAGAAAATTAACTTTACAGCACCAGGAAAAGGCTCAGACCCAGAAGTGGCATACATGTGGACTAACTGCA
176861_8776181_HIV2-Con2A_1_67	7854	7974	AGAGGAGAATTTCTCTACTGCAACATGACTTGGTTCCTCAATTGGGTAGAAAACAGGACGAATCAGACACAGCGCAATTATGCGCCGTGCCATATAAGGCAAATAATTAATACCTGGCAT
176861_8776181_HIV2-Con2A_1_68	7973	8093	TAAGGTAGGGAAAAATGTATATTTGCCTCCTAGGGAAGGAGAGTTGACCTGCAACTCAACAGTGACCAGCATAATTGCTAACATTGACGTGGATGAAAATCAGACAAATATTACCTTTAG
176861_8776181_HIV2-Con2A_1_69	8092	8212	GTGCAGAGGTGGCAGAACTATACCGATTAGAATTGGGGGATTATAAATTAGTAGAAATAACACCAATTGGCTTCGCACCTACATCAGAAAAAAGATACTCCTCTGCTCCAGGGAGACATA
176861_8776181_HIV2-Con2A_1_70	8211	8331	AAAAGAGGTGTGTTCGTGCTAGGGTTCTTGGGTTTTCTCGCAACAGCAGGTTCTGCAATGGGCGCGGCGTCCTTGACGCTGTCGGCTCAGTCCCGGACTTTACTGGCCGGGATAGTGCAG
176861_8776181_HIV2-Con2A_1_71	8330	8450	GCAACAGCAACAGCTGTTGGACGTGGTCAAGAGACAACAAGAAATGTTGCGACTGACCGTCTGGGGAACAAAAAATCTCCAGGCAAGAGTCACTGCTATCGAGAAGTACCTAAAGGACCA
176861_8776181_HIV2-Con2A_1_72	8449	8569	AGGCGCAGCTAAATTCATGGGGATGTGCGTTTAGACAAGTCTGCCACACTACTGTACCATGGGTAAATGATTCCTTAACACCTGATTGGAACAATATGACATGGCAGGAATGGGAACAAA
176861_8776181_HIV2-Con2A_1_73	8568	8688	AAAGTCCGCTACCTAGAGGCAAATATCAGTCAAAGTTTAGAACAGGCACAAATTCAGCAAGAAAAGAATATGTATGAACTACAAAAATTAAATAGCTGGGATGTTTTTGGCAACTGGTTT
176861_8776181_HIV2-Con2A_1_74	8687	8807	TGACTTAACCTCCTGGATCAAGTATATTCAATATGGAGTTTATATAGTAGTAGGAATAATAGCTTTAAGAATAGTAATATATGTAGTACAAATGTTAAGTAGACTTAGAAAGGGCTATAG
176861_8776181_HIV2-Con2A_1_75	8806	8926	GGCCTGTTTTCTCTTCCCCCCCCGGTTATATCCAACAGATCCATATCCACAAGGACCGGGAACAGCCAGCCAGAGAAGAAACAGAAGAAGACGTTGGAGACAACGGTGGAGACAGATCGT
176861_8776181_HIV2-Con2A_1_76	8925	9045	TGGCCTTGGCCGATAGCATATATACATTTCCTGATCCGCCTGCTGATTCGCCTCTTGACCGGACTATACAACATCTGCAGGGACTTACTATCCAGGATCTCCCTGACCCTCCAACCAATC
176861_8776181_HIV2-Con2A_1_77	9044	9164	CTTCCAGAGTCTCCAGAGAACACTGACAGCAATCAGAGACTGGCTGAGACTTAAAGCAGCCTACCTGCAATATGGGTGCGAGTGGATCCAAGAAGCGTTCCAGGCCTTCGCGAGGGCTAC
176861_8776181_HIV2-Con2A_1_78	9163	9283	CGAGAGAGACTCTTGCGAGCGCGTGGAGGGGCTTGTGGGGGGCATTGGGACGAATCGGGAGGGGAATACTCGCAGTCCCAAGAAGGATCAGGCAGGGAGCAGAAATCGCCCTCCTGTGAG
176861_8776181_HIV2-Con2A_1_79	9282	9402	GGGACAGCGGTATCAGCAGGGAGACTTTATGAATACCCCATGGAGAACCCCAGCAGCAGAAGGAGAGAAAAATTTATACAAGCAACAAAATATGGATGATGTAGATTCAGATGATGATGA
176861_8776181_HIV2-Con2A_1_80	9401	9521	ACCTAGTAGGAGTCTCTGTCACACCAAGAGTACCACTAAGAGCAATGACATATAAATTGGCAGTAGATATGTCACATTTTATAAAAGAAAAGGGGGGACTGGAAGGGATGTTTTACAGTG
176861_8776181_HIV2-Con2A_1_81	9520	9640	GAGAGAAGACATAGAATCCTAGACATATACTTAGAAAAGGAAGAAGGGATAATTCCAGATTGGCAGAATTATACTCATGGGCCAGGAGTAAGATACCCAATGTTCTTTGGGTGGCTATGG
176861_8776181_HIV2-Con2A_1_82	9639	9759	GAAGCTAGTACCAGTAGATGTCCCACAAGAGGGGGAGGACGATGAGACTCACTGCTTAATACACCCAGCACAAACAAGCAGGTATGATGACCCGCATGGGGAAACACTAGTCTGGAGGTT
176861_8776181_HIV2-Con2A_1_83	9758	9878	TTGACCCCATGCTGGCCTATGATTACAAGGCCTTTATTCTATACCCAGAGGAATTTGGGCACAAGTCAGGCCTGCCAGAAGAAGAGTGGAAGGCAAAACTGAAAGCAAGAGGGATACCAT
176861_8776181_HIV2-Con2A_1_84	9877	9997	TTTAGTTAAAGACAGGAACAGCTATACTTGGTCAGGGCAGGAAGTAGCTACTGAAAACAGCTGAGACTGCAGGGACTTTCCAGAAGGGGCTGTAACCAGGGGAGGGACATGGGAGGAGCT
176861_8776181_HIV2-Con2A_1_85	9996	10116	TGGTGGGGAACGCCCTCATACTTTCTGTATAAATGTACCCGCTGCTTGCATTGTATTCAGTCGCTCTGCGGAGAGGCTGGCAGATTGAGCCCTGGGAGGTTCTCTCCAGCACTAGCAGGT
176861_8776181_HIV2-Con2A_1_86	10115	10235	TAGAGCCTGGGTGTTCCCTGCTAGACTCTCACCAGTGCTTGGCCGGCACTGGGCAGACGGCTCCACGCTTGCTTGCTTAAAGACCTCTTAATAAAGCTGCCAGTTAGAAGCAAGTTAAGT
176861_8776181_HIV2-Con2A_1_87	10234	10353	TGTGTGTTCCCATCTCTCCTAGTCGCCGCCTGGTCATTCGGTGTTCACCTGAGTAACAAGACCCTGGTCTGTTAGGACCCTTCTTGCTTTGGGAAACCAAGGCAGGAAAATCCCTAGCA
176861_8776182_Con2A_6982-7101_1_1	1	120	ATGTGACAGAGGCTTTTGATGCATGGGATAATACAGTAACAGAACAAGCAATAGAAGATGTCTGGAATCTATTTGAGACATCAATAAAACCATGTGTCAAATTAACACCTTTATGTGTA
176861_8776183_Con2A_7249-7371_1_1	1	120	ACTGCTCAGGATTAGGGGAGGAAGAGATGGTCAATTGTCAGTTCAATATGACAGGATTAGAGAGAGATAAGAAAAAACAGTATAATGAAACATGGTACTCAAAAGATGTGGTTTGTGAA
176861_8776184_Con2A_7451-7570_1_1	1	120	TGATAAGCACTATTGGGATGCTATGAGGTTTAGATACTGTGCACCACCGGGTTATGCCCTGCTAAGATGCAATGATACCAATTATTCAGGCTTTGAGCCCAATTGCTCTAAAGTAGTAG
176861_8776185_HIV2B-cgConsensus_1_1	0	120	TGGAAGGGATTTACTATAGTGAGAGAAGACATAGAATACTAGACACATATCTAGAGAATGAGGAAGGCATTGTGTCTGGATGGCAAAACTATACACATGGGCCAGGGGTAAGGTATCCAA
176861_8776185_HIV2B-cgConsensus_1_2	119	239	AAGTCCTTTGGTTGGCTGTGGAAGCTAGTACCAGTAGATATGCCAGCAGAAGCACAGGAGGGAGAGGAAACCCATTGTCTGGTGCACCCAGCACAGACCTCTTCATGGGATGACCCCCAC
176861_8776185_HIV2B-cgConsensus_1_3	238	358	CGGGGAGACCCTTGTCTGGCAGTTTGATCCCCTCCTGGCATATGACTATGTGGCCTTCAACAGGTTCCCAGAAGAGTTTGGGTATCAGTCAGGACTACCAGAGAAGGAGTGGAAGGCTAG
176861_8776185_HIV2B-cgConsensus_1_4	357	477	GACTAAAAGCAAGAGGAATACCTACAGACTAGGCAGGAAACAGCAGCATAAACAGGAACTAGCTGACACTGCATAAGAAGGAAACTAGCTGACACTGCAGGGACTTTCCAAAAGGGGCTG

176861_8776185_HIV2B-cgConsensus_1_5	476	596	GTAACATGGGAGGGACATGGGAGGAGTTGGTGGGGAACGCCCATTAAATCTCTGTATAAATGTACCCGCTTTTTGCATTGTATTCAGTCGCTCTGCGGAGAGGCTGGCAGATTGAGCCCT
176861_8776185_HIV2B-cgConsensus_1_6	595	715	TGGGAGGTTCTCTCCAGCACTAGCAGGTAGAGCCTGGGTGTTCCCTGCTAGACTCTCACCAGCACTTGGCCGGTGCTGGGCAGACGGCTCCACGCTTGCTTGCTTAAAACCCTCTTAATA
176861_8776185_HIV2B-cgConsensus_1_7	714	834	AAAGCTGCCAATTAGAAGCAAGTTAAGTGTGTGTTCCCATCTCTCCTAGTCGCCGCCTGGTCATTCGGTGCTCCACTGAAATAAAGACCCTGGTCTGTTAGGACCCTTCTTGCTTTGGGA
176861_8776185_HIV2B-cgConsensus_1_8	833	953	AAACCAAAGCAGGAAAATCCCTAGCAGATTGGCGCCCGAACAGGGACTTGAAGAAGAGTGAGAGTCTTCGGAACACGGCTGAGTGAAGGCAGTAAGGGCGGCAGGAATCAACCACGACGG
176861_8776185_HIV2B-cgConsensus_1_9	952	1072	GAGAGCTCCTGTAAAGGCGCAGGCCGGTACCAGGCAGCGTGAGGAGCGGGAGGAGAAGAGGCCTCCGGGAGGAAAGGTAAGTACTCCAAATTTTGAATTGTCTACTCTAAAAAAGGAGTA
176861_8776185_HIV2B-cgConsensus_1_10	1071	1191	AGGGCATAAGTGGGAGATGGGCGCGAGAGGCTCCGTCTTGTCAGGGAAGAAAACAGATGAATTAGAAAAAGTTAGGTTACGGCCCGGCGGAAAGAAAAAGTATATGCTAAAACATGTAGT
176861_8776185_HIV2B-cgConsensus_1_11	1190	1310	TATGGGCAGTGAACGAATTGGACAGATTTGGATTAGCAGAGAGCCTGTTGGAATCAAAAGAAGGATGTCAAAAAATTCTTACGGTCTTAGCTCCATTAGTACCAACAGGATCAGAAAATT
176861_8776185_HIV2B-cgConsensus_1_12	1309	1429	TTAAAAAGCCTTTTTAATACTGTCTGCGTCATTTGGTGCTTGCACGCAGAAGAGAAAGTGAAAGATACTGAGGAAGCAAAAAAGATAGCACAGAGACATCTAGCGGCGGACACAGAAAAA
176861_8776185_HIV2B-cgConsensus_1_13	1428	1548	AATGCCAGCTACAAGTAAACCAACAGCACCACCTAGCGGCGGAAATTACCCAGTGCAGCAAGTAGCTGGCAATTATGTCCACCTGCCGCTAAGCCCCCGAACCTTAAATGCCTGGGTAAA
176861_8776185_HIV2B-cgConsensus_1_14	1547	1667	AATTAGTAGAAGAAAAGAAGTTCGGGGCAGAAGTAGTACCAGGATTTCAGGCACTATCAGAAGGATGCACCCCTTATGATATTAATCAGATGCTAAATTGTGTAGGAGAACATCAGGCAG
176861_8776185_HIV2B-cgConsensus_1_15	1666	1786	GCCATGCAAATTATTAGAGAAATAATCAATGAGGAAGCAGCAGACTGGGACCAGCAGCACCCGATACCAGGCCCACTGCCAGCAGGACAACTTAGGGACCCCAGAGGGTCAGATATAGCA
176861_8776185_HIV2B-cgConsensus_1_16	1785	1905	AGGAACCACCAGCACAGTAGAGGAACAGATACAGTGGATGTACAGGGCCCAAAATCCTGTCCCAGTAGGAAACATTTATAGAAGATGGATTCAATTAGGACTGCAGAAATGTGTCCGGAT
176861_8776185_HIV2B-cgConsensus_1_17	1904	2024	TGTACAATCCTACCAATATACTAGACATAAAGCAAGGGCCAAAAGAACCCTTCCAAAGCTATGTAGATAGATTCTACAAAAGCTTACGGGCAGAACAAACAGACCCAGCAGTGAAAAATT
176861_8776185_HIV2B-cgConsensus_1_18	2023	2143	TGGATGACACAAACACTGCTGATTCAGAATGCTAACCCAGATTGCAAGTTAGTACTTAAGGGCTTGGGAATGAACCCCACCTTAGAGGAAATGCTGACAGCCTGCCAAGGGATAGGAGGC
176861_8776185_HIV2B-cgConsensus_1_19	2142	2262	CCCAGGGCAAAAGGCAAGGCTAATGGCTGAGGCCTTAAAAGAAGCCTTAACACCTGCACCCATACCGTTTGCTGCCGCTCAACAAAAAGCAGGAAAGAGAGGGACAGTGACATGCTGGAA
176861_8776185_HIV2B-cgConsensus_1_20	2261	2381	ACTGTGGCAAAGGGGGACACACAGCCAGGCAATGCAGGGCCCCTAGAAGACAGGGATGCTGGAAATGTGGAAAACCAGGACACATCATGTCAAAATGCCCAGAAAGACAGGCGGGTTTTT
176861_8776185_HIV2B-cgConsensus_1_21	2380	2500	TTAGGGTTTGGACCCTGGGGAAAGAAGCCTCGCAACTTCCCCATGACCCAAGTGCCTCAGGGGGTGACGCCATCTGCACCCCCGATGAACCCAGCAGAGGGCATGACACCTCAGGGGGCG
176861_8776185_HIV2B-cgConsensus_1_22	2499	2619	GACACCATCTGCGCCCCCTGCAGATCCAGCAGTGGAGATGCTGAAAAACTACATGCAGCTGGGGAAGCAGCAGAGAGAGAGCCGAGAGAGACCCTACAAGGAGGTGACAGAGGATTTGCT
176861_8776185_HIV2B-cgConsensus_1_23	2618	2738	TGCACCTCAATTCTCTCTTTGGAGAAGACCAGTAGTCAAAGCATATATCGAGGGTCAGCCAGTAGAAGTATTACTAGACACAGGGGCTGATGACTCAATAGTAGCAGGAATAGAATTAGG
176861_8776185_HIV2B-cgConsensus_1_24	2737	2857	GTAGCAATTACACCCCAAAAATAGTAGGAGGAATAGGAGGATTCATAAATACCAAAGAATACAAAAATGTAGAAATAGAAGTAGTAGGAAAAAGAGTAAGAGCAACTATAATGACAGGAG
176861_8776185_HIV2B-cgConsensus_1_25	2856	2976	GACACCCCAATAAACATTTTTGGCAGAAATATTTTAAATACCTTAGGCATGACTCTAAATTTCCCAGTGGCAAAAATAGAACCAGTAAAAGTCAAGTTAAAACCTGGAAAAGATGGACCA
176861_8776185_HIV2B-cgConsensus_1_26	2975	3095	AAAAATCAGACAATGGCCTCTATCTAAAGAAAAGATACTAGCCCTCAAAGAAATCTGTGAAAAAATGGAAAAGGAGGGACAACTAGAAGAAGCACCCCCTACTAATCCATACAACACACC
176861_8776185_HIV2B-cgConsensus_1_27	3094	3214	CCACCTTTGCCATAAAGAAGAAGGACAAAAACAAATGGAGAATGCTAATAGATTTCAGAGAATTAAACAAGGTAACCCAAGACTTTACAGAAGTCCAACTGGGTATTCCCCACCCGGCAG
176861_8776185_HIV2B-cgConsensus_1_28	3213	3333	GGGCTGGCAGAAAAAAGGAGAATAACAGTACTAGATGTAGGAGATGCCTACTTCAGTATCCCACTAGATCCAGACTTCAGACAATATACAGCATTCACCTTGCCATCAGTAAATAATGCA
176861_8776185_HIV2B-cgConsensus_1_29	3332	3452	AGAGCCAGGAAAAAGATACATTTATAAAGTCCTACCACAAGGATGGAAGGGATCCCCAGCAATTTTTCAATACTCCATGAGGAAGGTACTAGACCCCTTCAGAAAAGCCAACAATGATGT
176861_8776185_HIV2B-cgConsensus_1_30	3451	3571	TCACTATAATCCAGTACATGGATGACATCCTTGTAGCAAGTGACAGAAGTGATCTAGAGCATGACAGGGTAGTGTCTCAACTAAAAGAGCTATTAAATGACATGGGATTCTCTACCCCAG
176861_8776185_HIV2B-cgConsensus_1_31	3570	3690	GAAGAAAAGTTCCAAAAAGACCCTCCATTCAAATGGATGGGTTATGAGCTCTGGCCAAAAAAGTGGAAACTGCAAAAAATACAACTGCCAGAAAAGGAAGTTTGGACAGTAAATGACATT
176861_8776185_HIV2B-cgConsensus_1_32	3689	3809	TCAGAAACTAGTGGGAGTACTAAACTGGGCAGCTCAACTTTTTCCTGGAATTAAGACAAGGCACATATGCAAACTAATCAGGGGAAAGATGACCCTAACAGAAGAAGTACAGTGGACAGA
176861_8776185_HIV2B-cgConsensus_1_33	3808	3928	AATTAGCAGAGGCAGAGTTGCAGGAAAATAAAATCATCCTAGAACAGGAACAGGAAGGATCCTACTACAAAGAAGGGGTACCTCTAGAAGCAACAGTGCAGAAAAACCTAGCAAATCAGT
176861_8776185_HIV2B-cgConsensus_1_34	3927	4047	TGGACATACAAAATTCATCAGGGAGATAGAATCCTAAAAGTAGGAAAATATGCAAAGGTTAAAAACACCCACACCAATGGAGTAAGACTACTGGCACATGTAGTCCAGAAAATAGGAAAA
176861_8776185_HIV2B-cgConsensus_1_35	4046	4166	AGAAGCACTAGTCATCTGGGGAGAAATACCAATGTTCCATCTGCCAGTAGAAAGAGAGACATGGGACCAGTGGTGGACAGATTACTGGCAAGTAACCTGGATCCCAGAATGGGATTTTGT
176861_8776185_HIV2B-cgConsensus_1_36	4165	4285	TCTCAACCCCACCATTAGTAAGACTAGCCTACAACCTAGTCAAAGACCCCCTAGAAGAGGTAGAAACCTACTACACAGATGGGTCCTGCAACAGAGCCTCAAAGGAAGGAAAAGCAGGAT
176861_8776185_HIV2B-cgConsensus_1_37	4284	4404	TATGTCACAGACAGGGGAAAAGATAAGGTTAAAGTGTTAGAGCAGACAACAAATCAACAAGCAGAACTTGAAGCATTTGCACTAGCATTACAGGATTCAGGACCACAAGTCAACATCATA
176861_8776185_HIV2B-cgConsensus_1_38	4403	4523	AGTAGATTCACAATATGTCATGGGAATAATAGCTGGACAGCCAACAGAAACAGAATCACCAATAGTAAATAAAATAATTGAAGAAATGATCAAAAAGGAAGCAATATATGTAGGATGGGT
176861_8776185_HIV2B-cgConsensus_1_39	4522	4642	TGCCAGCTCACAAAGGACTAGGTGGTAATCAGGAAGTAGACCACCTAGTAAGTCAAGGAATTAGACAGGTTCTATTCCTAGAAAAAATAGAACCAGCTCAAGAAGAACATGAAAAATATC
176861_8776185_HIV2B-cgConsensus_1_40	4641	4761	CATGGCAATGTAAAAGAACTAGTTCATAAATTTGGAATTCCACAATTAGTGGCAAAACAAATAGTAAATTCCTGTGATAAATGCCAACAAAAAGGAGAAGCTATTCATGGACAGGTAAAT
176861_8776185_HIV2B-cgConsensus_1_41	4760	4880	TGCAGAACTAGGGACATGGCAGATGGACTGTACACATTTAGAAGGAAAAATCATAATAGTGGCAGTCCATGTAGCCAGTGGGTTTATAGAAGCAGAAGTAATACCCCAAGAAACAGGAAG
176861_8776185_HIV2B-cgConsensus_1_42	4879	4999	GACAGACAGCTCTCTTCCTGTTAAAGTTGGCCAGCAGATGGCCTATCACACACCTGCACACAGACAATGGTGCCAACTTCACCTCACAAGATGTGAAGATGGTAGCCTGGTGGGTAGGAA
176861_8776185_HIV2B-cgConsensus_1_43	4998	5118	ATAGAACAAACCTTTGGAGTACCCTATAACCCACAAAGTCAGGGAGTAGTAGAAGCAATGAACCATCATCTAAAAAATCAAATAGATAGAATCAGAGACCAGGCAGTATCAATAGAGACA
176861_8776185_HIV2B-cgConsensus_1_44	5117	5237	AGTTGTACTAATGGCAGCTCACTGCATGAATTTTAAAAGAAGGGGAGGAATAGGGGATATGACCCCTGCAGAGAGACTAGTTAACATGATAACCACAGAACAAGAAATACAATTCTTCCA
176861_8776185_HIV2B-cgConsensus_1_45	5236	5356	AAGCAAAAAATTTAAAATTTCAAAATTTCCAGGTCTATTACAGAGAAGGCAGAGATCAACTCTGGAAGGGACCTGGTGAGCTATTGTGGAAAGGGGAAGGAGCAGTCATCATAAAGGTAG
176861_8776185_HIV2B-cgConsensus_1_46	5355	5475	GGGACAGAAATCAAAGTAATACCCAGAAGAAAAGCAAAGATCATAAGGCACTATGGAGGAGGAAAAGAATTGGATTGTAGTGCCGACATGGAGGATACCAGGCAGGCTAGAGAGATGGCA
176861_8776185_HIV2B-cgConsensus_1_47	5474	5594	ACAGTCTGGTCAAGTACCTGAAGCATAGAACAAGAGAGTTGCAACAGGTCTCTTATGTCCCCCACCACAAGGTAGGATGGGCTTGGTGGACTTGCAGTAGAGTAATATTCCCCCTAAAAG
176861_8776185_HIV2B-cgConsensus_1_48	5593	5713	GAAGGAGCATATCTAGAGGTCCAAGGATATTGGAATCTGACCCCGGAAAGGGGATTCTTGAGTTCCTATGCTGTAAGACTAACATGGTATAAAAGGAGCTTTTATACAGATGTAACACCT
176861_8776185_HIV2B-cgConsensus_1_49	5712	5832	TGATGTGGCAGACCAGCTACTGCATGGGTCTTATTTCTCTTGCTTTACAGCCAATGAAGTAAGGAGAGCCATCAGGGGAGAAAAGATATTGTCCTACTGCAACTATCCATCAGCTCACGA
176861_8776185_HIV2B-cgConsensus_1_50	5831	5951	AAGGGCAGGTACCAAGTTTACAGTTTCTAGCCTTAAGAGTTGTACAAGAAGGAAAAAATGGATCCCAGGGAGAGAGTGCCACCAGGAAACAGCGACGAAGAAACAATAGGAGAGGCATTC
176861_8776185_HIV2B-cgConsensus_1_51	5950	6070	CGAATGGCTAGAAAGAACAATAACAGAGCTCAACAGGGAAGCAGTCAACCATTTGCCCCGAGAACTCATTTTCCAGGTCTGGCAAAGGTCTTGGGCATATTGGCGTGAGGAACAGGGCAT
176861_8776185_HIV2B-cgConsensus_1_52	6069	6189	TGTCAATTAGCTACACCAAATATAGATACTTGTTTCTAATGCAAAAAGCAATGTTTGTGCATTTTACAAAGGGCTGTACATGCCTGCGGGAGGGCCATGGGCCAGGGGGATGGAGACCAG
176861_8776185_HIV2B-cgConsensus_1_53	6188	6308	GGACCTCCTCCTCCTCCTCCCCCAGGCCTAGCCTAATGGCAGAAGCAGCCCCAGAGACTCCTCCAGAGAATGAGGACCCACAGAGAGAGCCATGGGAAGAGTGGGTAGAGGACGTCCTGG
176861_8776185_HIV2B-cgConsensus_1_54	6307	6427	GAGGAAATAAAGCAAGAAGCCTTAAAGCATTTTGATCCCCGCTTGCTAACTGCGCTTGGTAACTTTATCTACAGTAGGCATGGAGATACCCTTGCAGGAGCAGGAGAACTCATTAAAATC
176861_8776185_HIV2B-cgConsensus_1_55	6426	6546	CCTCCAACGAGCGCTCTTCCTCCACTTCAGAGGCGGTTGTCAACACTCAAGGATTGGACAACCAGGGGGAGGAAATCCTCTCTCAGCTATACCGCCCTCTTGAGGCATGCAATAATAAAT
176861_8776185_HIV2B-cgConsensus_1_56	6545	6665	TGCTACTGTAAGAAATGCTGCTACCATTGCCAGCTTTGTTTTCTTAAAAAGGGCCTTGGGATATGTTATGACCGCTCGAGAAAAAGATCTTCAAAAAGGGCTAAGGCTACTGCATCTTCT

176861_8776185_HIV2B-cgConsensus_1_57	6664	6784	TGCATCAGACAAGTGAGTATGGCACATATTAGCAATCACCTGCTTATTGCGCTCCTGCTTATAAGTGTCTATGGGTTTTTGTGTAAACAATATGTCACTGTCTTCTATGGCATACCCGCA
176861_8776185_HIV2B-cgConsensus_1_58	6783	6903	ATGGAGGAATGCAACAGTTCCCCTCTTTTGTGCAACCAAAAACAGAGATACCTGGGGAACTATACAGTGCCTCCCAGACAATGATGATTATACCGAAATCGCTCTAAATGTAACAGAGGC
176861_8776185_HIV2B-cgConsensus_1_59	6902	7022	CTTTTGATGCATGGAATAATACAGTAACAGAACAGGCAGTAGAGGATGTGTGGAGTCTTTTTGAGACCTCAATAAAACCATGTGTCAAGCTAACACCACTATGTGTGGCAATGAACTGTA
176861_8776185_HIV2B-cgConsensus_1_60	7021	7141	AGTAAAACTGAGACAAACCTAACAGGAAGCAGTAGTACCACCACTACCCCCACTACTACCACTACTCAAAAACTACTCAATGAAACAAACCCATGTATAAAAAATGACAACTGCACAGGA
176861_8776185_HIV2B-cgConsensus_1_61	7140	7260	AATAGGACTAGAAAAAATGGTTGACTGTCAATTTAATATGACAGGACTAAGAAGAGATGAGAAAAAACAATATAATGACACCTGGTACTCACAAGATTTAGTGTGTGAAAATACCAGGAA
176861_8776185_HIV2B-cgConsensus_1_62	7259	7379	AGAATAATGACAGCAGATGCTATATGAAAACCTGTAATACATCAGTTATCCAAGAGTCATGTGACAAACATTATTGGGATAGCTTAAGATTTAGGTACTGTGCTCCCCCAGGATTTGCTT
176861_8776185_HIV2B-cgConsensus_1_63	7378	7498	TTGCTAAGATGTAATGATACCAACTATTCAGGCTTTATGCCCAACTGTAGTAAAGTAGTAGTATCCTCTTGCACAAGAATGATGGAAACACAGACCTCTACATGGTTTGGCTTCAATGGT
176861_8776185_HIV2B-cgConsensus_1_64	7497	7617	TACAAGGGCAGAAAATAGGACATATATATATTGGCATGGTAGAGATAATAGGACTATCATAAGCTTAAATAAGTATTATAATTTAACAATACACTGTAGAAGGCCAGGAAACAAGACAGT
176861_8776185_HIV2B-cgConsensus_1_65	7616	7736	TTGTACCAATAACAATCATGTCAGGGCTAGTGTTCCATTCACAGCCTATCAATAAAAGACCCAGACAAGCTTGGTGCTGGTTTAAAGGAAACTGGACAGAAGCCATAAAGGAGGTGAAGG
176861_8776185_HIV2B-cgConsensus_1_66	7735	7855	GAGACCATTATAAAACATCCCAGGTATAAAGGAACAAAAAATATCACAAAGATAAATTTGGCAGAGCCCGCAGCAGGTTCAGATCCAGAAGTAACATACATGTGGACTAACTGCAGAGGA
176861_8776185_HIV2B-cgConsensus_1_67	7854	7974	AGAATTCTTCTACTGTAATATGACTTGGTTTTTAAACTGGGTAGAAAACAGAACTGGTACAACTAAGAGAAATTATGTGCCGTGCCACATCAGACAGATAATCAACACGTGGCACAAGGT
176861_8776185_HIV2B-cgConsensus_1_68	7973	8093	TAGGAAAAAATGTATATTTGCCTCCAAGGGAAGGTGAGCTCACCTGTAATTCCACTGTCACCAGCCTCATAGCCAACATTGACTGGATTGATAACAGGAACCATACCAATATTACCATGA
176861_8776185_HIV2B-cgConsensus_1_69	8092	8212	AGTGCAGAAGTGGCAGAACTGTATAGATTGGAATTGGGGGACTACAAATTAGTAGAAATAACACCAATTGGCTTTGCACCTACAAATGTAAAAAGATATTCCTCAGCGACACCGAGGAAT
176861_8776185_HIV2B-cgConsensus_1_70	8211	8331	TAAAAGAGGTGTATTTGTGCTAGGGTTCTTGGGATTTCTTGCAACGGCAGGTTCTGCAATGGGCGCAGCGTCCTTGACGCTGTCAGCTCAGTCCCGGACTTTACTGGCTGGGATAGTGCA
176861_8776185_HIV2B-cgConsensus_1_71	8330	8450	AGCAGCAGCAACAGCTGTTGGACGTGGTCAAAAGACAACAGGAACTGTTGCGGCTGACCGTCTGGGGGACGAAAAACCTCCAGACAAGAGTCACTGCCATCGAGAAATACCTAAAGGACC
176861_8776185_HIV2B-cgConsensus_1_72	8449	8569	CAGGCACAACTAAATTCATGGGGATGTGCTTTTAGACAGGTCTGCCACACTACTGTACCATGGCCAAATGAAACTCTCACACCAAACTGGGACAACATGACATGGCAACAATGGGAAAAA
176861_8776185_HIV2B-cgConsensus_1_73	8568	8688	ACAAGTCCACTACTTGGATGCAAATATAACAGAACTATTAGAAGAGGCACAGATACAACAAGAAAAGAACATGTATGAATTACAGAAATTAAATAGCTGGGATGTTTTTGGTAATTGGTT
176861_8776185_HIV2B-cgConsensus_1_74	8687	8807	TTGACTTCACCTCCTGGATAAAATATATCAAGTTAGGACTATATGTAGTAGTAGGATTAATAGTGTTAAGAATAGTAATATACATTGTACAGATGCTAGCAAGACTTAGGAAGGGCTATA
176861_8776185_HIV2B-cgConsensus_1_75	8806	8926	AGGCCAGTATTCTCCTCCCCTCCCTCTTATATTCAACAGATCCCTATCCACAAGGACCGGGGACAGCCAGCCAACGAAGAAACAGAAGAAGGAGGTGGAAACGACGGGGGCTACAGATCT
176861_8776185_HIV2B-cgConsensus_1_76	8925	9045	TTGGCCCTGGCAGATAGAATACATTCATTTCCTGATTCGCCAGCTGAGGAACCTCTTGATTTGGCTATACAACGGCTGCAGAACCTTACTATTGAAGACCTTCCAAATCCTCCAACCAGT
176861_8776185_HIV2B-cgConsensus_1_77	9044	9164	TACTCCAACCACTCAGGCTTCCACTTGCATACCTCCAGTATGGGATCAGCTGGTTCCAAGAAGCAATCCAAGCAGCAGCGAGGGCTGCGGGAGAGACTCTTGCGAGCGCGGGGAGGGCCT
176861_8776185_HIV2B-cgConsensus_1_78	9163	9283	TTATGGGAGGCTCTCAGAAGAGCGGCGAGAGCAATCATCGCGATCCCCAGGAGGATCAGACAAGGACTTGAACTCGCCCTCTTGTGAGGGACAAAATGCCCCAGGAGCAGAAGGGGGAGG
176861_8776185_HIV2B-cgConsensus_1_79	9282	9402	GGCAACAAGATATAGATGATAGTGATGAGGATGATGAAGTGGGAGTCTATGTAAGACCCAATATGCCACTGAGACCAATGACATACAAATTGGCAATAGACATGTCTCATTTTATAAAAG
176861_8776185_HIV2B-cgConsensus_1_80	9401	9521	GAAAAGGGGGGACTGGAAGGGATTTTTTATAGTGAGAGAAGACATAGAATATTAGACACATACTTAGAGAATGAGGAAGGCATTGTATCTGGATGGCAGAACTATACACATGGGCCAGGG
176861_8776185_HIV2B-cgConsensus_1_81	9520	9640	GATAAGATATCCAAAGTCCTTTGGCTGGCTGTGGAAGCTAGTACCAGTAGATGTACCAGAAGAAGAGGAGGAAGAGGAGACCCATTGTCTGGTGCACCCAGCACAGACCTCTTCATGGGA
176861_8776185_HIV2B-cgConsensus_1_82	9639	9759	ATGACCCCCATGGGGAGACCCTTGTCTGGCAGTTTGATTCCCTCCTGGCATATGACTATGTGGCCTTCAACAGGTTCCCAGAAGAGTTTGGGTATCAGTCAGGGCTACCAGAGAAAGAGT
176861_8776185_HIV2B-cgConsensus_1_83	9758	9878	TGGAAGGCTAGACTAAAAGCAAGAGGAATACCTACAGATTAGGCAGGAAACAGCAGCATAAAGAGGAACTAGCTGACACTGCATAAGAAGAAACTAGCTGACACTGCAGGGACTTTCCAA
176861_8776185_HIV2B-cgConsensus_1_84	9877	9997	AAAGGGGCTGTAACATGGGAGGGACATGGGAGGAGTTGGTGGGGAACGCCCATTAAATCTCTGTATAAATGTACCCGCTTTTTGCATTGTATTCAGTCGCTCTGCGGAGAGGCTGGCAGA
176861_8776185_HIV2B-cgConsensus_1_85	9996	10116	ATCGAGCCCTGGGAGGTTCTCTCCAGCACTAGCAGGTAGAGCCTGGGTGTTCCCTGCTAGACTCTCACCAGCACTTGGCCGGTGCTGGGCAGACGGCTCCACGCTTGCTTGCTTAAAACC
176861_8776185_HIV2B-cgConsensus_1_86	10115	10235	CCTCTTAATAAACTGCCAATTAGAAGCAAGTTAAGTGTGTGTTCCCATCTCTCCTAGTCGCCGCCTGGTCACTCGGTGCTCCACTGAAATAAAGACCCTGGTCTGTTAGGACCCTTTCTG

Table S2A. Consensus sequence identity

	HIV Reads	SMART	SMART+xGen	SMART_dup removed	SMART+xGen_dup remov
**PBS5635**					
SMART	12653		99.90%	99.90%	99.90%
SMART+xGen	522043	99.90%		99.80%	99.90%
SMART_dup removed	12155	99.90%	99.80%		99.80%
SMART+xGen_dup removed	242912	99.90%	99.90%	99.80%	
**P406**					
SMART	4676		99.10%	99.90%	99.70%
SMART+xGen	629912	99.10%		99.10%	99.30%
SMART_dup removed	4614	99.90%	99.10%		99.70%
SMART+xGen_dup removed	531814	99.70%	99.30%	99.70%	
**PBS1189**					
SMART	2260		99.80%	99.70%	99.70%
SMART+xGen	463644	99.80%		99.70%	99.80%
SMART_dup removed	2111	99.70%	99.70%		99.80%
SMART+xGen_dup removed	98446	99.70%	99.80%	99.80%	
**50**					
SMART	1731		98.90%	99.40%	99.20%
SMART+xGen	218072	98.90%		99.30%	99.50%
SMART_dup removed	1693	99.40%	99.30%		99.70%
SMART+xGen_dup removed	92015	99.20%	99.50%	99.70%	
**87-2580**					
SMART	5741		99.70%	99.80%	99.70%
SMART+xGen	221335	99.70%		99.70%	99.80%
SMART_dup removed	5565	99.80%	99.70%		99.80%
SMART+xGen_dup removed	190384	99.70%	99.80%	99.80%	

Table S2B. Minor Variant analysis

	% genome coverage	Total # minor variants	SMART	SMART+xGen	SMART_dup removed	SMART+xGen_dup removed
**PBS5635**						
SMART	95	103		71, 74.0% (71/96), 79.8% (71/89)	96, 95.0%(95/101), 97.9% (95/97)	
SMART+xGen	99	89				85, 95.5% (85/89), 92.4% (85/92)
SMART_dup removed	95	97				79, 78.2% (79/101), 84.0% (79/94)
SMART+xGen_dup removed	99	92				
**P406**						
SMART	98	210		122, 54.2% (122/225), 64.2% (122/190)	181, 92.3% (181/196), 98.4% (181/184)	
SMART+xGen	100	190				186, 96.9% (186/192), 87.3% (186/213)
SMART_dup removed	98	184				152, 79.2% (159/192), 71.4% (152/213)
SMART+xGen_dup removed	100	213				
**PBS1189**						
SMART	82	31		10, 28.6% (10/35), 33.3% (10/30)	27, 90.0% (27/30), 100.0% (27/27)	
SMART+xGen	86	30				20, 95.2% (20/21), 90.9% (20/22)
SMART_dup removed	82	27				8, 30.8% (8/26), 38.1% (8/21)
SMART+xGen_dup removed	86	22				
**50**						
SMART	83	116		35, 35% (35/100), 53.8% (35/65)	106, 80.9% (106/131), 95.5% (106/111)	
SMART+xGen	83	65				62, 93.9% (62/66), 91.2% (62/68)
SMART_dup removed	83	111				43, 43% (43/100), 70.5% (43/61)
SMART+xGen_dup removed	83	68				
**87-2580**						
SMART	96	95		68, 55.3% (68/123), 63% (68/108)	61, 70.3% (61/86), 82.4% (61/74)	
SMART+xGen	96	108				71, 87.7% (71/81), 75.5% (71/94)
SMART_dup removed	96	74				8, 44.4% (8/18), 57.1% (8/14)
SMART+xGen_dup removed	96	94				
